# Nucleation and Ostwald Growth of Particles in Fe-O-Al-Ca Melt

**DOI:** 10.1038/s41598-018-19639-w

**Published:** 2018-01-18

**Authors:** Linzhu Wang, Junqi Li, Shufeng Yang, Chaoyi Chen, Huixin Jin, Xiang Li

**Affiliations:** 1 College of Materials and Metallurgy, Guizhou University, Guiyang, 550025 China; 2 School of Metallurgical and Ecological Engineering, University of Science and Technology Beijing, Beijing, 100083 China; 3 College of Materials & Metallurgical Engineering, Guizhou Institute of Technology, Guiyang, 550003 China

## Abstract

Tremendous focus has been put on the control of particle size distribution which effects the grain structure and mechanical properties of resulting metallic materials, and thus nucleation and growth of particles in solution should be clarified. This study uses classical nucleation theory and Ostwald ripening theory to probe the relationship between the compositions of Fe-O-Al-Ca melts and the behavior of particles under the condition of no external stirring. Our experimental data suggest that decreasing the initial Ca addition and Al addition is conductive to the increase of nucleation rate for calcium aluminate particles, which exhibits a same change trend with that predicted from classical nucleation theory. Based on the experimental evidence for particles size distribution in three-dimensional, we demonstrate that Ostwald ripening is the predominate mechanism on the coarsening of particles in Fe-O-Al-Ca melt at early stage of deoxidation under the condition of no external stirring but not at later stage.

## Introduction

Controlling on the characteristics of particles in the metallic materials has been one of the leading subjects in the field of metallurgy which directly effects the progress of melting^1^, mechanical properties and service life of final products^2,3^. In spite of efforts dedicated to the utmost removal of the particles, they still exist in metallic materials^4,5^. In recent years, focus is shifting from the removal of particles to effective utilization of fine particles, aiming at refining the microstructure, and improving the strength and fracture toughness^6–8^. Particle-assisted microstructure control has been frequently used in the metallic materials^6,9–11^. Ma *et al*.^12^ reported a novel Al matrix composite with ultrahigh strength reinforced by a three dimensional network of nano-AlN particles. Hossein^13^ found that the ferrite was grain-refined to about 3 μm due to vitue of augmented nucleation and retarded growth by titanium oxide nanoparticles. Fine MgO-containing particles were found to have a facilitating effect on the formation of equi-axed crystallization and refinement of microstructure^14^. Yiquan Wu^15^ found that as Ca content increased from 2 ppm to 25 ppm in thick plates, particles in submicron scale were 6 times as much as that in conventional steel, which promoted the nucleation of acicular ferrite obviously. Integrated performance of HAZ for steel plates was improved significantly by retarding γ grain growth in the HAZ near a weld fusion line with fine dispersed oxides and/or sulfides containing Ca or Mg^16,17^. It should be noted that the transformation, augmented nucleation and retarded growth of grain by pinning are strongly influenced by the size distribution of fine particles. Many scholars advocate that it is particularly important to obtain the fine particles in sub-micrometer or nanometer scale whose number density is considerably large and volume fraction is small^18–20^. From this point of view, it is critical to investigate the nucleation and growth behavior of particles in the metallic materials.

In spite of extensive studies on the nucleation of inorganic particles in many fields^21–26^, it remains a matter of debates. Jian Zhang *et al*.^27^ gave a numerical analysis of alumina particles by combining thermodynamics, classical homogeneous nucleation theories and dynamics of particles collision and coagulation, and reported that the nucleation process in a Fe-Al-O melt system covers only several tens of microseconds. The nucleus of alumina particles were predicted to be about 10–20 Å in diameter and their nucleation time should be in the range of 1–10 μs based on thermodynamic analysis and numerical simulation by Lifeng Zhang *et al*.^28^. However, Lindberg *et al*.^29^ found that the time for attending the 90% of the equilibrium of particle volume was 0.2 s based on the diffusion model in Si deoxidation. The growth mechanism of deoxidation products can be explained by the following four major processes^18^: diffusion growth; coagulation due to the difference in ascending velocity; the coagulation due to Brown motion and the coarsening by Ostwald ripening. It is reported that the growth of particles in molten steel by diffusion occurs very rapidly and far less than 60 s at 1600 °C^18^. Kluken^30^ and Suzuki *et al*.^31^ concluded that the growth of particles in steel should be explained by Ostwald ripening. In addition, Ohta and Suito^32,33^ have investigated the size distribution of CaO- Al_2_O_3_ particles in Fe-10mass%Ni alloy and found that compared with Al_2_O_3_, the distribution curve of CaO- Al_2_O_3_ was narrower and nucleation rate was higher, indicating that CaO- Al_2_O_3_ particles were fine and in large amount in Fe-10mass%Ni alloy. They also advocated that the supersaturation degree, and interfacial energy between oxide particles and liquid Fe, and the equilibrium deoxidation constant affect the nucleation and growth of particles in early stage of deoxidation under no coagulation of deoxidation particles by collision. However, they just compared the size distribution of deoxidation products of MgO, ZrO_2_, Al_2_O_3_, CaO-Al_2_O_3_ and MnO-SiO_2_ in an Fe-10mass%Ni alloy. In spite of many experiments performed to investigate the formation mechanism and composition control of particles in steel by thermodynamic and kinetic theories^34–36^, limited studies about the effect of melt composition on the nucleation and growth of particles are conducted.

In current study, the relationships between compositions of Fe-O-Al-Ca melts with not only the particle type, but also the particle size distribution were analyzed. The nucleation and growth by Ostwald ripening of particles in Fe-O-Al-Ca melt were estimated and verified by experimental data. This study will provide information to predict the nucleation and growth of particles in the melt and will be helpful for controlling behavior of particle.

## Results and Discussion

### Experimental results

**Figure 1:**
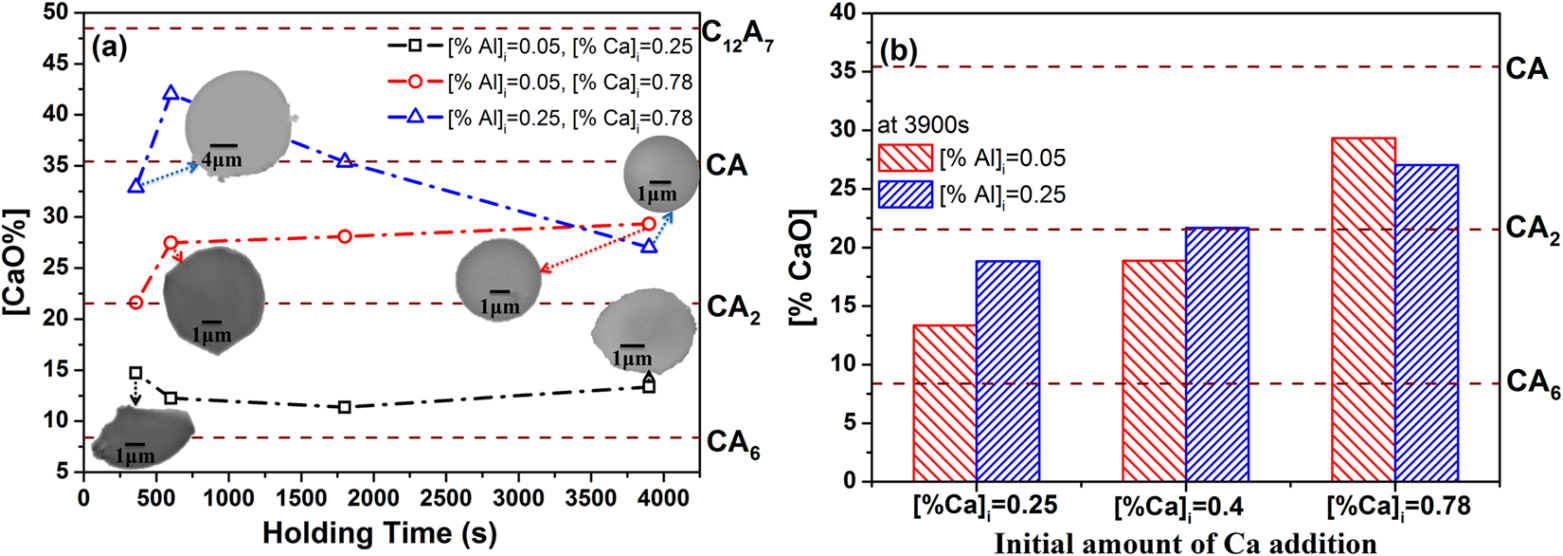
Typical morphologies and average compositions of particles in samples. (**a**) SEM images of typical particles and average composition evolution of calcium aluminates during whole deoxidation process at 1600 °C. (**b**) Average compositions of particles in steels after deoxidation at 1600 °C for 3900 s.

The average compositions and morphologies of particles in Fe-O-Al-Ca melt during the deoxidation process are shown in Fig. 1. [% Ca]_i_ = 0.25, 0.4, 0.78 (i represents initial addition of metal) were added in the melts with [% Al]_i_ = 0.05, 0.25 in order to study the effect of deoxidants amount on the behavior of calcium aluminate particles in the melt. The yield rates of Al and Ca in this experiment are about 90% and 1.6%, respectively. The chemical compositions of samples are analyzed and presented in Tables 1–2. The energy dispersive spectroscopy results reveal that the major particles in the Fe-O-Al-Ca melt are Al_2_O_3_-CaO. The content of CaS is no more than 5%, thus, it is ignored. Figure 1a presents the SEM images of typical particles and their average composition evolution in the melt during deoxidation process. The SEM-EDS results of typical particles in A1C1 and A2C3 can be found as Supplementary Figure S1. The samples were taken at 1600 °C after deoxidation for 360 s, 600 s, 1800 s and 3900 s and timing started at Al powder added. Experiment A1C1 ([% Al]_i_ = 0.05 and [% Ca]_i_ = 0.25) exhibits predominantly solid CaO·6Al_2_O_3_ (melting point is 1850 °C)^37^ +CaO·2Al_2_O_3_ (melting point is 1750 °C)^37^ particles with irregular shape during the whole melting process. The particles in experiment A1C3 ([% Al]_i_ = 0.05 and [% Ca]_i_ = 0.78) evolve from CaO·2Al_2_O_3_ to partially liquid CaO·2Al_2_O_3_ + CaO·Al_2_O_3_ (melting point is 1605 °C)^37^ particles with spherical shape, and the evolution trajectory of particles in experiment A2C3 ([% Al]_i_ = 0.25 and [% Ca]_i_ = 0.78) is CaO·2Al_2_O_3_ + CaO·Al_2_O_3_ → CaO·Al_2_O_3_ + 12CaO·7Al_2_O_3_ → CaO·Al_2_O_3_ → CaO·2Al_2_O_3_ + CaO·Al_2_O_3_. Figure 1b illustrates the compositions of particles in the melts with various contents of deoxidants at 3900 s. It can be seen that the average CaO content of particles increases with increasing amount of Ca addition. Most of particles are composed of CaO·6Al_2_O_3_ and CaO·2Al_2_O_3_ after melting for 3900 s, except for the melts with initial Ca addition of 0.78% which exhibits predominantly CaO·2Al_2_O_3_ + CaO·Al_2_O_3_ particles.

**Table 1:** Experimental condition and main chemical compositions of samples in deoxidation experiments.

Exp. No	Deoxidants	Holding time at 1600°C (s)	O_T_,ppm	[O], ppm	[Ca], ppm	[Al], ppm
A1C1	0.05%Al + 0.25%Ca (0.83%SiCa)	360	161.6–165.2	5.6–9.2	1.7	360
		600	128.1–128.6	0.8–1.3	0.7	300
		1800	72.1–75.7	2.4–2.8	4.7 × 10^–5^	290
		3900	68.2–72.2	2.4	4.7 × 10^–5^	290
A1C2	0.05%Al + 0.4%Ca (1.33%SiCa)	3900	42.2–43.8	2.5	4.5 × 10^−5^	480
A1C3	0.05%Al + 0.78%Ca (2.6%SiCa)	360	126–127.7	0.4–2.7	36.4	940
		600	71.4–74.4	4.5–7.4	5.7	940
		1800	41.1–43.4	0.9–2	0.0012–5.0	950
		3900	29.0–30.3	0.9–1.2	0.0012–5.4	950
A2C1	0.25%Al + 0.25%Ca (0.83%SiCa)	3900	53.3–53.6	0.8–0.6	1.4 × 10^−4^	1900
A2C2	0.25%Al + 0.4%Ca (1.33%SiCa)	3900	38.9–39.1	0.8	1.5 × 10^−4^	2000
A2C3	0.25%Al + 0.78%Ca (2.6%SiCa)	360	106–110.6	0.9–3.1	68.1	2100
		600	70.3–72.1	0.2–1.1	10.8	2100
		1800	25.9–27.2	0.5–3.2	0.00327–6.1	2200
		3900	20.4–20.7	0.5–1.1	0.00327–6.0	2200

**Table 2:** Other chemical compositions of final samples.

Exp. No	C	Si	Mn	P	S	Cu	Ni
A1C1	0.0015	0.0026	0.01	0.004	0.0019	0.0038	0.0035
A1C2	0.0014	0.0029	0.01	0.005	0.0016	0.0036	0.0035
A1C3	0.0016	0.0028	0.01	0.004	0.0014	0.0037	0.0038
A2C1	0.0016	0.0029	0.01	0.007	0.0017	0.0035	0.0037
A2C2	0.0015	0.0025	0.01	0.005	0.0014	0.0036	0.0039
A2C3	0.0017	0.0028	0.01	0.006	0.0013	0.0036	0.0034

**Figure 2:**
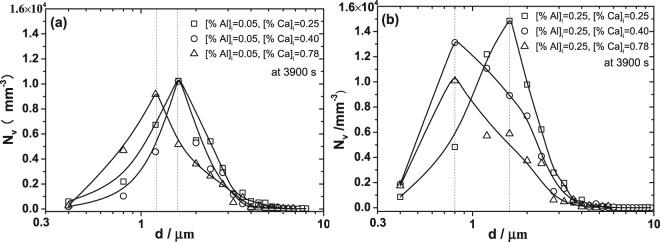
Particle size distributions in three-dimensional of different samples at 1600 °C after deoxidation for 3900 s. (**a**) Is for steels with initial Al added amount of 0.05% and (**b**) is for steels with initial Al added amount of 0.25%.

**Figure 3:**
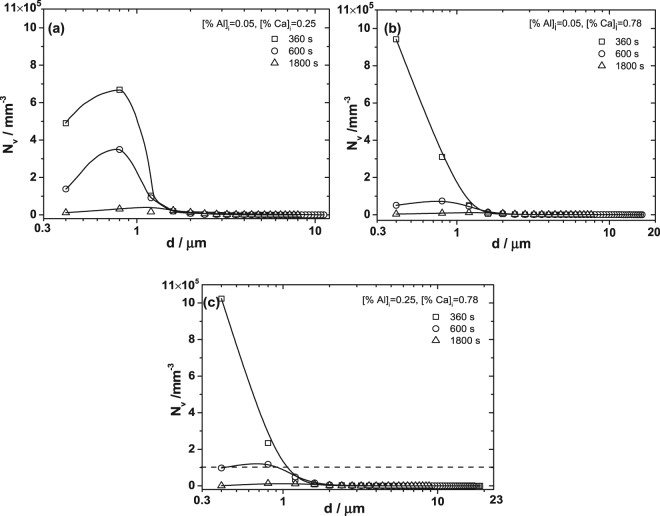
Particle size distribution in three-dimensional for different samples after deoxidation at 1600 °C for 360 s, 600 s and 1800 s. (**a**) Is for steels with [%Al]_i_ = 0.05, [%Ca]_i_ = 0.25. (**b**) Is for steels with [%Al]_i_ = 0.05, [%Ca]_i_ = 0.78. (**c**) Is for steels with [%Al]_i_ = 0.25, [%Ca]_i_ = 0.78.

Figure 2 depicts the particle size distribution in three-dimensional for the experimental samples at 3900 s based on the stereological analysis^38,39^. It can be seen that the peak of the curves in Fig. 2 tends to decrease with the increasing amount of initial Ca addition, indicating that the number density of particles decreases with increasing Ca at 3900 s. Besides, the size of particles corresponding to the peaks of curves in the samples containing higher Ca content is smaller than that in the melts containing lower Ca content. It is concluded that the number density and size of particles tend to decrease with an increase of initial Ca addition after deoxidation for 3900 s. The particle size distribution for different samples after deoxidation at 1600 °C for 360 s, 600 s and 1800 s are plotted in Fig. 3. The particles with size less than 260 nm can’t be detected due to the limitation of resolution of SEM. Therefore, the curves of particle size distribution in Fig. 3b,c are incomplete. However, it still can be seen that the primary particles are smaller and in significantly larger amount in the melt with high initial Ca addition ([Ca%]_i_ = 0.78), compared with those in the melt with low initial Ca addition ([Ca%]_i_ = 0.25). The number of particles decreases significantly and large particles form with the proceeding of deoxidation, attributing to the floatation, aggregation and growth of particles.

### Calculation results

#### Nucleation of Calcium Aluminates

In order to study the contents of Al, Ca and O on the nucleation rates of calcium aluminates, *I*_*CxAy*_ (cm^−3^·s^−1^), it was estimated as the following relationship based on the classical nucleation theory^32^:

1 $$\mathrm{ln}\,{I}_{{C}_{x}{A}_{y}}=\frac{16\pi {\gamma }_{{C}_{x}{A}_{y}-Melt}^{3}{V}_{O({C}_{x}{A}_{y})}^{2}}{3{k}_{B}{R}^{2}{T}^{3}}(\frac{1}{{(\mathrm{ln}{S}_{{C}_{x}{A}_{y}}^{\ast })}^{2}}-\frac{1}{{(\mathrm{ln}{S}_{{C}_{x}{A}_{y}})}^{2}})$$

where *V*_*O*(*CxAy*)_ is the molar volume of oxide (m^3^/mol), *k*_*B*_ is the Boltzman constant (1.38 × 10^−23^ J·K^−1^), R is the gas constant (8.314 J·mol^−1^·K^−1^) and T is the absolute temperature (K). *γ*_*CxAy-Melt*_ is interfacial energy between calcium aluminates and metallic melt (J/m^2^).

According to the research of Li^40^ and Suito^32^, supersaturation degree of calcium aluminate *S*_*CxAy*_ can be expressed by Eq. (2). *K*_*CaO*_ is calculated from the relation: log *K*_*CaO*_ = *−*9.08^41^ (1600 °C). In this calculation, the effect of melt compositions on the activity coefficient of Ca and O is not considered.

2 $${S}_{C{\rm{x}}A{\rm{y}}}=\frac{{a}_{Ca}\cdot {a}_{O}}{{a}_{CaO}}\cdot {K}_{CaO}$$

S^*^_CxAy_ is the critical supersaturation degree which is the value of *S*_*CxAy*_ at I = 1 (cm^−3^·s^−1^) and it is derived from Eq. (3). A is the frequency factor (10^26^ cm^−3^·s^−132^). *a* and *b* are used to modify the calculated values by matching that with the experimental critical supersaturation degree^32^ (*a* = 0.026, *b* = 1.25). The values of S^*^_CxAy_ obtained by Eq. (3) are shown in Table 1.

3 $${S}_{CxAy}^{\ast }=\exp (\frac{{V}_{O(CxAy)}}{RT}\sqrt{\frac{16\pi {\gamma }_{PL}^{3}}{3{k}_{B}T\,\mathrm{ln}\,A}})\times a+b$$

The interfacial energy between solid particles and metallic melt can be expressed by Young’s equation:

4 $${\gamma }_{{C}_{{\rm{x}}}{A}_{y}-Melt}={\gamma }_{{C}_{{\rm{x}}}{A}_{y}}-{\gamma }_{Melt}\,\cos \,\theta $$

The interfacial energy between liquid particle and metallic melt can be calculated by Neumann’s relation^42^:

5 $${{\gamma }^{{\rm{2}}}}_{{C}_{{\rm{x}}}{A}_{y}-Melt}={{\gamma }^{{\rm{2}}}}_{{C}_{{\rm{x}}}{A}_{y}}+{{\gamma }^{{\rm{2}}}}_{Melt}-2{\gamma }_{{C}_{{\rm{x}}}{A}_{y}}{\gamma }_{Melt}\,\cos \,\phi $$

where *γ*_*CxAy*_ and *γ*_*Melt*_ are the surface energies of calcium aluminate and metallic melt (J/m^2^), *θ* is the contact angle between solid particle and melt, *φ* is the visible contact angle of liquid particle. The relation between *φ* and θ^*^ (the contact angle between liquid particle and melt) can be expressed by Eq. 6^42^ if vertical equilibrium is considered.

6 $${\gamma }_{{C}_{{\rm{x}}}{A}_{y}-Melt}\,\sin \,({\theta }^{\ast }-\phi )={\gamma }_{{C}_{{\rm{x}}}{A}_{y}}\,\sin \,\phi $$

The surface energy of metallic melt (J/m^2^) used in this study was calculated at 1600 °C as following^43–46^:

7 $${\gamma }_{Melt}=1.75-0.279\,\mathrm{ln}\,(1+140\cdot {a}_{O})$$

The relevant parameters^45,47–52^ used in the calculation of nucleation rates for calcium aluminates are shown in Table 3. The activities of Al_2_O_3_ and CaO in particles were estimated by Factsage Software 7.0 of “Equilib” module. The calcium aluminates with mole ratio of Al_2_O_3_/CaO at the range from 1 to 3 are almost in liquid state at 1600 °C and their visible contact angle is calculated by Eq. 6. In this study, CA type particle is treated as liquid because it is often in partly or totally molten state in the experiments of measuring wettability^53^ and it is observed to be spherical or semi- spherical in the samples. The surface energies of solid calcium aluminates are estimated by the relation:

8 $${\gamma }_{C{\rm{x}}Ay}=x\cdot {\gamma }_{CaO}+y\cdot {\gamma }_{Al2O3}$$

where x and y are the molar fractions of CaO and Al_2_O_3_ in calcium aluminates. *γ*_*CaO*_ and *γ*_*Al2O3*_ are the surface energies of solid CaO (0.74 J/m^2^ ^47^) and solid Al_2_O_3_ (0.94 J/m^2^ ^45^) at 1600 °C.

**Table 3:** Relevant parameters of calcium aluminates used in this study.

Type	ρ, g/cm^−3^	V_o_, m^3^/mol	*γ*_*CxAy*_, J/m^2^	θ, deg	θ^*^(φ), deg	*a*_*Al2O3*_	*a*_*CaO*_	C_P(O)_, kg·m^−3^	C_P(Ca)_, kg·m^−3^	So^*^
CA_6_	3.79^50^	9.3	0.9^45,47^	126^51^	—	1	0.005	1725	227	1.9–5.3
CA_2_	2.92^50^	12.7	0.87^45,47^	120^48^	—	0.88	0.011	1256	448	1.9–4.8
CA	2.56^49^	15.4	0.62^52^	—	74^47,48^ (69)	0.264	0.113	1037	648	1.9–25
C_12_A_7_	2.69^49^	15.6	0.63^52^	—	59^48^ (40)	0.047	0.449	1025	932	1.3–1.4

ρ is density. V_o_ is molar volume of oxide. *γ*_*CxAy*_ is surface energy. θ is the contact angle between solid particle and melt. *φ* is the visible contact angle of liquid particle. θ^*^ is the contact angle between liquid particle and melt. *a* is activity. C_P_ is oxygen or calcium concentration in oxide expressed by weight per unit volume. So^*^ is the critical supersaturation degree.

**Figure 4:**
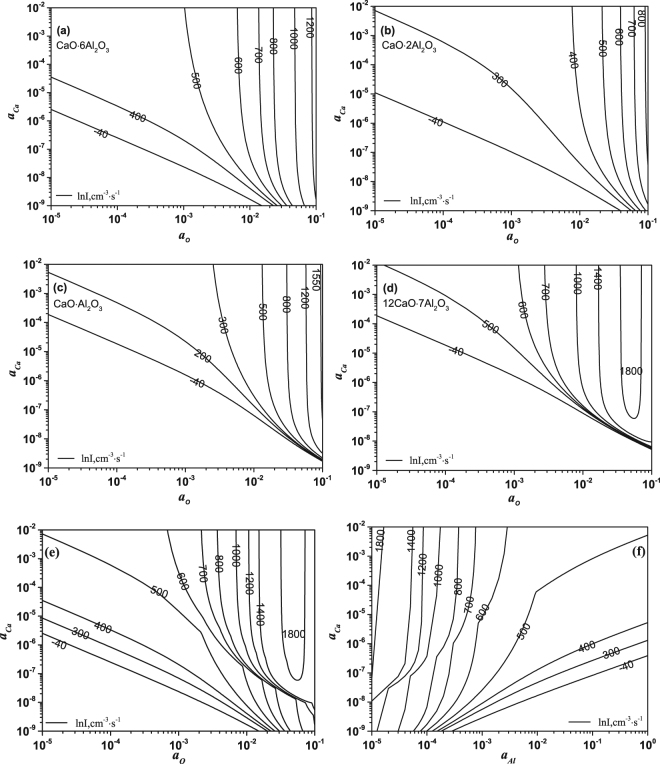
Calculated nucleation rates of calcium aluminates in Fe-O-Al-Ca melt at 1600 °C. (**a**–**d**) Relationship of *a*_Ca_ and *a*_O_ when CaO·6Al_2_O_3_, CaO·2Al_2_O_3_, CaO·Al_2_O_3_ and 12CaO·7Al_2_O_3_ nucleate at various rates, repectively. (**e**) Relationship of *a*_Ca_ and *a*_O_ when all kinds of calcium aluminates nucleate at various rates simultaneously. (**f**) Relationship of *a*_Ca_ and *a*_Al_ when all kinds of calcium aluminates nucleate at various rates simultaneously. The nucleation rate in (**e)** and (**r**) represents the total nucleation rate of all types of calcium aluminates.

Combining Eqs (1–8) at 1600 °C, the effect of melt compositions (*a*_*o*_ and *a*_*Ca*_) on the nucleation rate of each type of calcium aluminate, including CaO·6Al_2_O_3_, CaO·2Al_2_O_3_, CaO·Al_2_O_3_ and 12CaO·7Al_2_O_3_ is shown in Fig. 4a–d, respectively. The nucleation rate of CaO·2Al_2_O_3_ for given activities of Ca and O is larger than that of CaO·6Al_2_O_3_, and the nucleation rates of various types of calcium aluminates in the region with high supersaturation degree (*a*_*o*_ > 0.03 and *a*_*Ca*_ > 10^−6^) increase in the order of CaO·2Al_2_O_3_ < CaO·6Al_2_O_3_ < CaO·Al_2_O_3_ < 12CaO·7Al_2_O_3_.

Figure 4e,f is obtained by adding the nucleation rates of all types of calcium aluminates together which means that it is assumed that these calcium aluminates nucleate simultaneously. Assuming that [Al] is in equilibrium with [O] before calcium aluminates nucleate, the effect of *a*_*Al*_ on the nucleation rates of calcium aluminates can be calculated based on the thermodynamic equilibrium relation: Al_2_O_3_ = 2[Al] + 3[O] (log K_Al2O3_ = −12.57^41^). *a*_*Al*_ is determined from the relationship: ${K}_{A{l}_{2}{O}_{3}}=\frac{{a}_{Al}^{2}\cdot {a}_{o}^{3}}{{a}_{A{l}_{2}{O}_{3}}}$ (Al_2_O_3_ = 2[Al] + 3[O], log K_Al2O3_ = −12.57. For CA_2_, ${a}_{Al}=\sqrt{\frac{2.37\times {10}^{-13}}{{a}_{o}^{3}}}$). Figure 4a–d suggests that the nucleation rate of each type of calcium aluminates increases with an increase of *a*_*o*_ and *a*_*Ca*_, and decrease of *a*_*Al*_. Figure 4e,f indicates that in the region where *a*_*o*_ is larger than 2 × 10^−3^ or *a*_*Al*_ is smaller than 2 × 10^−3^, the total nucleation rate is mainly dependent on the value of *a*_*o*_ or *a*_*Al*_ and it reaches the maximum (about 1800) when *a*_*o*_ is 6 × 10^−2^.

In order to verify the guiding significance of the nucleation theory on the metal smelting, the theoretical nucleation rates (ln I) of calcium aluminates in experiments were calculated based on the EDS results of particle compositions as shown in Table 4 and the activities of O, Al and Ca are obtained by substituting compositions of melt and thermodynamic data in Table 5 (thermodynamic data in Table 5 are derived from ref.^37^) to Eqs (9–10)^41^. The theoretical nucleation rates changed little as shown in Table 4 obtained by considering the effects of *a*_*o*_ and *a*_*Al*_ separately, which verifies that Eq. (2) is well applied in this study and the reaction between O and Al reaches equilibrium state nearly before adding Ca in this condition. The reason why the theoretical nucleation rate of calcium aluminates in the melt with lower amount of Ca addition is larger than that with higher amount of Ca addition is mainly attributed to the discrepancy of particle composition.

9 $${a}_{i}={f}_{i}[mass\,\,\, \% i]$$

10 $$\mathrm{log}\,{f}_{i}=\sum {e}_{i}^{j}[mass\,\,\, \% j]+{r}_{i}^{j,k}[mass\,\, \% j][mass\,\, \% k]$$

where *a*_*i*_, *f*_*i*_ and [*mass i* %] are the 1 mass% activity, 1 mass% activity coefficient, and the concentration of *i* in mass fraction, respectively. ${e}_{i}^{j}$ is the first-order interaction coefficient and ${r}_{i}^{j,k}$ is the second-order interaction coefficient.

**Table 4:** Experimental and calculated nucleation results.

Exp. No.	*a*_*o*_	*a*_*Al*_	*a*_*Ca*_	CA_6_, wt	CA_2_, wt	CA, wt	ln I*-a*_O_, cm^−3^·s^−1^	ln I-*a*_Al_, cm^−3^·s^−1^	ln $\bar{{\bf{I}}}$, cm^−3^·s^−1^	*f*_*V*(*n*)_, 10^−4^	*r*_*C*(*CxAy*)_, nm
A1C1	7.8 × 10^−4^	0.0360	9.1 × 10^−5^	29	71	—	484	479	43.8	9.0	0.277
A1C3	3.1 × 10^−4^	0.0949	1.6 × 10^−2^	—	99	1	313	312	41.7	0.2	0.248
A2C3	2.7 × 10^−4^	0.2143	2.3 × 10^−2^	—	6	94	310	308	41.0	0.1	0.267

*a*_*o*,_ *a*_*Al*,_ *a*_*Ca*_ is activity of O, Al and Ca. CA_x_ is the weight percentage of various calcium aluminate partilces in samples. ln I*-a*_O_ is theoretical nucleation rate of particles based on the relationship of *a*_*o*_ and *a*_*Ca*_. ln I*-a*_Al_ is theoretical nucleation rate of particles based on the relationship of *a*_*o*_ and *a*_*Al*_. ln $\bar{{\rm{I}}}$ is mean values of experimental nucleation rates for particles in samples. *f*_*V*(*n*)_ is volume fraction of particles in samples. *r*_*C*(*CxAy*)_ critical size of nuclei for particles.

**Table 5:** First-order and second-order interaction coefficients^41^ e^j^_i_ and r_i_^(j,k)^ of various elements in liquid steel at 1600 °C.

e^j^_i_/ r_i_^(j,k)^	C	Si	Mn	P	S	O	Al	Ca	Ca, Ca	O, Al	Al, Al	Ca, O	O, O
O	−0.421	−0.066	−0.021	0.07	−0.133	−0.174	−1.17	−313	570000	302	−0.01	−18000	—
Al	0.091	0.056	−0.004	0.033	0.035	−1.98	0.043	−0.047	—	−0.0284	—	—	39.8
Ca	−0.34	−0.095	−0.007	−0.097	−28	−780	−0.072	−0.002	—	—	—	−90000	650000

The mean values of experimental nucleation rates ($\overline{{\rm{I}}}$) in Exp. A1C1, Exp. A1C3 and Exp. A2C3 were obtained with Eqs. (11 and 12):

11 $$\overline{I}=\frac{{f}_{V({\rm{n}})}}{\frac{4}{3}\pi {r}_{C\,({C}_{x}{A}_{y})}^{3}\cdot t}$$

*f*_*V*(*n*)_ is the equilibrated volume fraction of nucleus and it can be estimated by the following relationship: *f*_*V*(*n*)_ = *f*_*V*(*Ca*)_ − *f*_*V*(*Al*)_ (*f*_*V*(*Ca*)_ is the volume fraction of all particles in the melt after Ca adding, that is, the volume fraction of paticles at 360 s. *f*_*V*(*Al*)_ is the volume fraction of Al_2_O_3_ just before Ca adding). The time for equilibrium of nucleus volume is 0.2 s^18^ and the critical size of nuclei *r*_*C*(*CxAy*)_ is the given by

12 $${r}_{C({C}_{x}{A}_{y})}=\frac{2{\gamma }_{{C}_{x}{A}_{y}-Steel}\cdot {V}_{O({C}_{x}{A}_{y})}}{RT\,\mathrm{ln}\,{S}_{{C}_{x}{A}_{y}}}$$

The calculated results suggest that the critical size of nuclei for calcium aluminates is about 0.2–0.3 nm. The experimental nucleation rates of calcium aluminates decrease in the order of experiments A1C1< A1C3< A2C3, exhibiting a same change trend with theoretical values, which indicates that decreasing the initial Ca addition and Al addition is conductive to the increase of nucleation rate for calcium aluminate. The average nucleation rate of calcium aluminates is smaller than that predicted from classical nucleation theory, presumably attributed to the underestimation of t and overestimation of local composition of melt.

#### Growth of Calcium Aluminates by Ostwald Ripening

Ostwald growth of calcium aluminates in Fe-O-Al-Ca melt controlled by oxygen diffusion can be expressed as following^18^:

13 $${\overline{r}}_{t}^{3}-{\overline{r}}_{0}^{3}=\alpha \cdot {k}_{{d}({O})}\cdot t$$

14 $${k}_{d(O)}=\frac{2{\gamma }_{{{C}}_{{x}}{{A}}_{{y}}{-}\mathrm{Steel}}{D}_{O}{V}_{O({C}_{x}{A}_{y})}{C}_{O}}{RT({C}_{P({C}_{x}{A}_{y})}-{C}_{O})}$$

where ${\overline{{r}}}_{{t}}$ and ${\overline{{r}}}_{{0}}$ are the mean radius of particles at time t (m) and that at the start of Ostwald growth (m), respectively. k_d_ is coarsening rate (μm^3^·s^−1^). *D*_*O*_ is the diffusion constant of oxygen (2.91 × 10^−9^ m^2^·s^−1^), *C*_O_ is the dissolved oxygen concentration expressed by weight per unit volume (kg·m^−3^) and *C*_*P*(*CxAy*)_ is the oxygen concentration in oxide expressed by weight per unit volume (kg·m^−3^). α is the coarsening rate coefficient. In previous study^54^, it is found that calculated coarsening rate is more accurate by using α_LSW_ from LSW theory instead of α_CN_ from communicating neighbour (CN model). Therefore, α values as 4/9 in this study.

**Figure 5:**
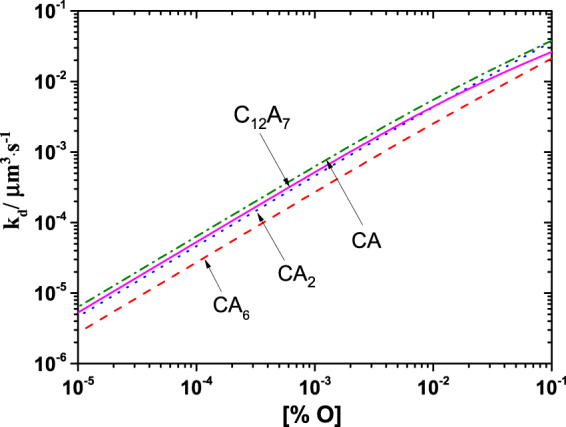
Relationship of coarsening rate caused by Ostwald ripening k_d_ for calcium aluminates and dissolved oxygen content [% O] in Fe-O-Al-Ca melt, calculated at 1600 °C.

The coarsening rate k_d_ of each type calcium aluminate was calculated by substituting the relevant data listed in Table 3 into Eq. (14) and are plotted with oxygen content as shown in Fig. 5. As can be seen, the coarsening rate increases with increasing dissolved oxygen content. Besides, for a given dissolved oxygen (less than 300 ppm), an increase of C/A ratio in calcium aluminates increases their values of k_d_ (except for CA) which increases in the order of CA_6_ < CA_2_ < C_12_A_7_ < CA.

**Figure 6:**
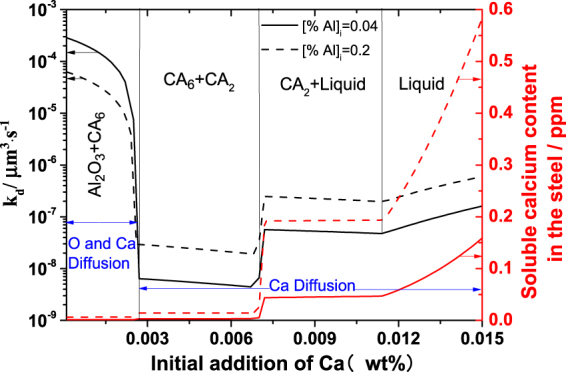
Effect of Ca addition on coarsening rate caused by Ostwald ripening k_d_ of particles and soluble Ca content in Fe-O-Al-Ca melt with various Al contents at equilibrium when Al_2_O_3_, CA_6_, CA_2_ and liquid calcium aluminate form as stable compounds, calculated at 1600 °C and based on FACTSAGE calculation.

The effect of Ca addition on the Ostwald growth of particles in Fe-O-Al-Ca melt with [Al]_i_ of 0.04% and 0.2% was obtained by considering the oxygen diffusion and calcium diffusion as shown in Fig. 6. The coarsening rate k_d(Ca)_ can be expressed by Eq. (15) in which the notations are similar to Eq. (14). D_Ca_ is assumed to be equal to the D_O_ because the solute diffusivities in liquid Fe is considered to be the same order of magnitude^18^.

15 $${k}_{d(C{\rm{a}})}=\frac{2{\gamma }_{{{C}}_{{x}}{{A}}_{{y}}{-}Steel}{D}_{C{\rm{a}}}{V}_{Ca({C}_{x}{A}_{y})}{C}_{C{\rm{a}}}}{RT({C}_{P({C}_{x}{A}_{y},C{\rm{a}})}-{C}_{C{\rm{a}}})}$$

Ca diffusion will be the rate determining step when k_d(Ca)_ is smaller than k_d(O)_ and vice versa. Figure 6 is obtained based on Eqs (14) and (15), in combination with FactSage modeling. Equilibrium compositions of melt with Ca addition are estimated by FACTSAGE 7.0 with the FactPS and FToxid and FTmisc databases. (based on the compositions of raw materials).“Equilib” module is used, and pure solids, and Fe-liq and A liquid slag in solution phases are selected as products. Calculated temperature and pressure are set as 1600 and 1 atm, respectively. Al diffusion will not be the rate determining step due to its high concentration in this work. It is found that with the increasing amount of added Ca (0–0.015%), the content of soluble Ca at equilibrium increases at the range from 0 to 0.6 ppm, and the coarsening rate of particles derived from Ostwald ripening decreases firstly and then increases as the liquid calcium aluminates form and increase. The Ostwald growth of Al_2_O_3_ is determined by O diffusion, while Ca diffusion is the rate determining step for the coarsening of calcium aluminates at equilibrium which is marked by blue line in Fig. 6. The value of k_d_ decreased slightly with an increase of Ca addition in the “CA6 + CA2” region ([% Ca]_i_ = 0.0027–0.0068) due to the increasing proportion of CA2. Besides, it indicates that the optimum amount of Ca addition for inhibiting the Ostwald growth of calcium aluminate particles is 0.0027–0.0068%. In addition, when the amount of initial calcium addition is larger than 0.0027%, the coarsening rate increases with the increasing amount of Al addition because equilibrated calcium increases and Ca diffusion is the rate determining step in this case.

**Figure 7:**
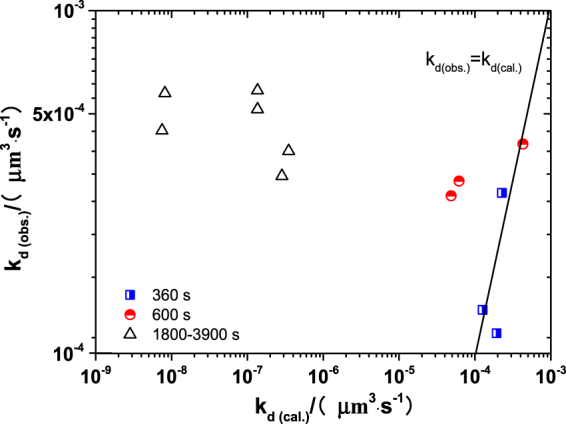
Comparison of experimental coarsening rate k_s(obs.)_ from particle size in samples and calculated coarsening rate k_s(cal.)_ from Ostwald ripening at 1600 °C.

The observed coarsening rates are obtained by substituting the experimental data into Eq. (13) and are plotted with the calculated values (obtained by substituting the relevant data in Table 3 and the composition of samples in Tables 1–2 to Eqs (14 and 15)) as shown in Fig. 7. It is found that k_d(cal.)_ tends to increase with increasing k_d(obs.)_ during the first 600 s of deoxidation process. The data at 360 s and 600 s in Fig. 7 fall around the line k_d(cal.)_ = k_d(obs.)_, although there is some deviation in those data probably caused by error of measurement for particle size (nano scale particles are excluded by Image-Proplus during the analysis of particle characteristics). In addition, it should be noted that the triangular points in Fig. 7 at later stage of melting are out of line completely. It can thus be concluded that the Ostwald ripening is the predominate mechanism of coarsening for calcium aluminate particles in Fe-O-Al-Ca melt during the first 600 s after aluminum addition under the condition of no external stirring but not at later stage of Al-Ca deoxidation. The mechanism on coarsening of calcium aluminate particles in Fe-O-Al-Ca melt at later stage of deoxidation is still going on.

## Conclusion

The behavior of particles in Fe-O-Al-Ca melt under the condition of no external stirring at 1600 °C was systematically studied using experimental methods, stereological method, classical nucleation theory, as well as Ostwald ripening theory.

The nucleation rate of calcium aluminates is dependent on their type and the composition of melt. It increases with an increase of *a*_o_ and *a*_Ca_, and decrease of *a*_Al_. Our experimental data suggest that decreasing the initial Ca addition and Al addition is conductive to the increase of nucleation rate for calcium aluminate, which exhibits a same change trend with that predicted from classical nucleation theory. Based on Ostwald ripening theory, for a given dissolved oxygen (less than 300 ppm), coarsening rate of particles in Fe-O-Al-Ca melt increases in the order of CA_6_ < CA_2_ < C_12_A_7_ < CA. The optimum amount of Ca addition for inhibiting the coarsening of calcium aluminates in Fe-O-Al-Ca melt is 0.0027–0.0068%. It is experimentally confirmed that the Ostwald ripening is the predominate mechanism of coarsening for calcium aluminate particles in Fe-O-Al-Ca melt during the first 600 s after aluminum addition under the condition of no external stirring but not at later stage of Al-Ca deoxidation. The mechanism on coarsening of calcium aluminate particles in Fe-O-Al-Ca melt at later stage of deoxidation is still going on.

## Methods

### High temperature experiments

High-purity iron was used as raw materials in the experiment and its chemical composition (wt.%) is 99.95% Fe, 0.0016% C, 0.0033% Si, 0.01% Mn, 0.0053% P, 0.0017% S, 0.003% Al, 0.0037% Cu, 0.0038% Ni. Al powder packed in iron foil (Al >99%) was first added in the molten steel at 1600 °C and after 5 min, Si-Ca alloy (59% Si, 30% Ca) was added for deoxidation, immediately stirred by a molybdenum rod for 5 s. All the experiments were carried out in Si-Mo heating electric resistance furnace without external stirring after adding Si-Ca alloy. Samples were taken by quartz tubes (Φ6 mm) for certain holding time which were injected with Ar gas firstly to prevent molten steel from being oxidized by air, followed by rapid quenching in salt water. During the whole melting process, the argon gas was controlled at the flow rate of 5 L/min.

### Characterization of particles

The compositions and morphologies of particles were observed using scanning electron microscopy with energy-dispersive spectrometric detection (SEM-EDS). The weight percentages of Al_2_O_3_ and CaO in particles were calculated based on the stoichiometric relationship and contents of Al, Ca, O which were measured by EDS. The stereological analysis (modified Schwartz-Saltykov method with the probability mass function^38,39^) was adopted to obtain the particle size distribution in three-dimensional from that in two-dimensional. The details are described as below^54^: the back-scattered electron pictures of each steel sample were taken under 1000 times corresponding to the area of 271 µm × 271 µm. 169 successive microphotographs were obtained by designating a step of 271 µm. Besides, the planar size and number of inclusions were analyzed by Image- ProPlus software^55^. The probability mass function (PMF) is expressed as following^37^:

16 $$P(r/R)=\frac{\delta {\rm{z}}}{R}=\frac{1}{R}(\sqrt{({R}^{2}-{({r}_{i}-{\rm{\Delta }}r)}^{2})}-\sqrt{{R}^{2}-{r}_{i}^{2}})$$

where P is the probability of a cross section with radius r (r_i_ − Δr < r < r_i_) intersecting a sphere with radius R which is the actual radius of inclusions in three-dimensional, and Δr is interval of groups. In this study, inclusions were classified into 49 successive groups from the largest inclusions based on the measured mean radius of inclusions in two-dimensional. The diameter of largest inclusions detected in all samples is no more than 19.6 μm. Therefore, the radium of inclusions in group 1 denoted by r = 9.8 μm or d = 19.6 μm is in the range of 9.7–9.9 μm, group 2 is denoted by r = 9.6 μm and group 49 is denoted by r = 0.2 μm. For group 49, r_i_ = 0.3 μm and Δr = 0.2 μm. According to the study of Li Tao^38^, the detected two-dimensional inclusions in group j probably belong to the three-dimensional group i (i ≤ j) as expressed by Eq. (17).

17 $${N}_{A}({j})={\sum }_{i=1}^{j}{d}_{i}{N}_{V}(i)P(j,i)$$

where N_A_ and N_V_ are the number density of inclusions in two-dimensional and three-dimensional, respectively. The transformation from two-dimensional spherical inclusion size distribution to three-dimensional inclusion size distribution can be performed based on Eqs (18), and (19) is P matrix. (P^−1^ is inverse matrix of P matrix)

18 $$[\begin{array}{c}{{\rm{d}}}_{1}{N}_{V}(1)\\ {{\rm{d}}}_{2}{N}_{V}(2)\\ {{\rm{d}}}_{3}{N}_{V}(3)\\ \vdots \\ {{\rm{d}}}_{{\rm{n}}}{N}_{V}({\rm{n}})\end{array}]{=P}^{-1}[\begin{array}{c}{N}_{A}(1)\\ {N}_{A}(2)\\ {N}_{A}(3)\\ \vdots \\ {N}_{A}({\rm{n}})\end{array}]$$

19 $$[\begin{array}{ccccccc}P(1,1) &  &  &  &  &  & \\ P(2,1) & P(2,2) &  &  &  &  & \\ P(3,1) & P(3,2) & P(3,3) &  &  &  & \\ P(4,1) & P(4,2) & P(4,3) & P(4,4) &  &  & \\ P(5,1) & P(5,2) & P(5,3) & P(5,4) & P(5,5) &  & \\ \cdots  & \cdots  & \cdots  & \cdots  & \cdots  & \cdots  & \\ P(n,1) & P(n,2) & P(n,3) & \cdots  & \ldots  & P(n,n-1) & P(n,n)\end{array}]$$

### Detection of sample compositions

The compositions of samples were detected by the ICP-AES method (for the detection of Al and Ca, etc.), infared absorption method after combustion in an induction furnace (for the analysis of sulfur) and Leco analyzer (for the measurement of total oxygen). The initial oxygen in all the experiments was 170 ± 20 ppm. The insoluble oxygen, alumina, and calcium contents were calculated based on Eqs (16–20).

20 $$[O{(M)}_{I{\rm{n}}sol.}]={f}_{V}\cdot \frac{{\rho }_{{M}_{x}{O}_{y}}}{{\rho }_{Fe}}\cdot \frac{y{M}_{O}(x{M}_{M})}{{M}_{{M}_{x}{O}_{y}}}\times {10}^{6}$$

where *f*_*V*_ is the total volume fraction of oxide inclusions, *ρ*_*Fe*_ is the density of Fe and *ρ*_*MxOy*_ is the density of the oxide inclusions (*ρ*_*Fe*_ = 7.8 g/cm^3^, *ρ*_*Al2O3*_ = 3.97 g/cm^−3^, *ρ*_*CaO*_ = 3.4 g/cm^−3^). *ρ*_*Al2O3-CaO*_ = *X*_*Al2O3*_ · *ρ*_*Al2O3*_ + *X*_*CaO*_ · *ρ*_*CaO*_. M_MxOy_ and X_MxOy_ are the molecular weight of M_x_O_y_ and the molar fraction of M_x_O_y_^33^.

### Data availability

The data that support the findings of this study are available from Linzhu Wang upon reasonable request.

## Electronic supplementary material

Supplementary information
