# Supplementary material for: Nucleation and Ostwald Growth of Particles in Fe-O-Al-Ca Melt
Source: Sci Rep. 2018 Jan 18;8:1135. doi: 10.1038/s41598-018-19639-w (PMC5773526; doi:10.1038/s41598-018-19639-w)
Supplement: Supplementary file 1 — Supplementary information [file 41598_2018_19639_MOESM1_ESM.pdf]

Nucleation and Ostwald Growth of Particles in Fe-O-Al-Ca Melt

Linzhu Wang[1], Junqi Li[1*], Shufeng Yang[2*], Chaoyi Chen[1], Huixin Jin[1], and Xiang Li[3]

1. School of Materials and Metallurgy, Guizhou University, Guiyang, Guizhou 550025, China
2. School of Metallurgical and Ecological Engineering, University of Science and Technology Beijing, Beijing 100083, China
3. College of Materials & Metallurgical Engineering, Guizhou Institute of Technology, Guiyang, 550003, China

*. Corresponding author, Email: Junqi Li, jqli@gzu.edu.cn; Shufeng Yang, yangshufeng@ustb.edu.cn;

SEM-EDS results of sample A1C1-360s

Image 1

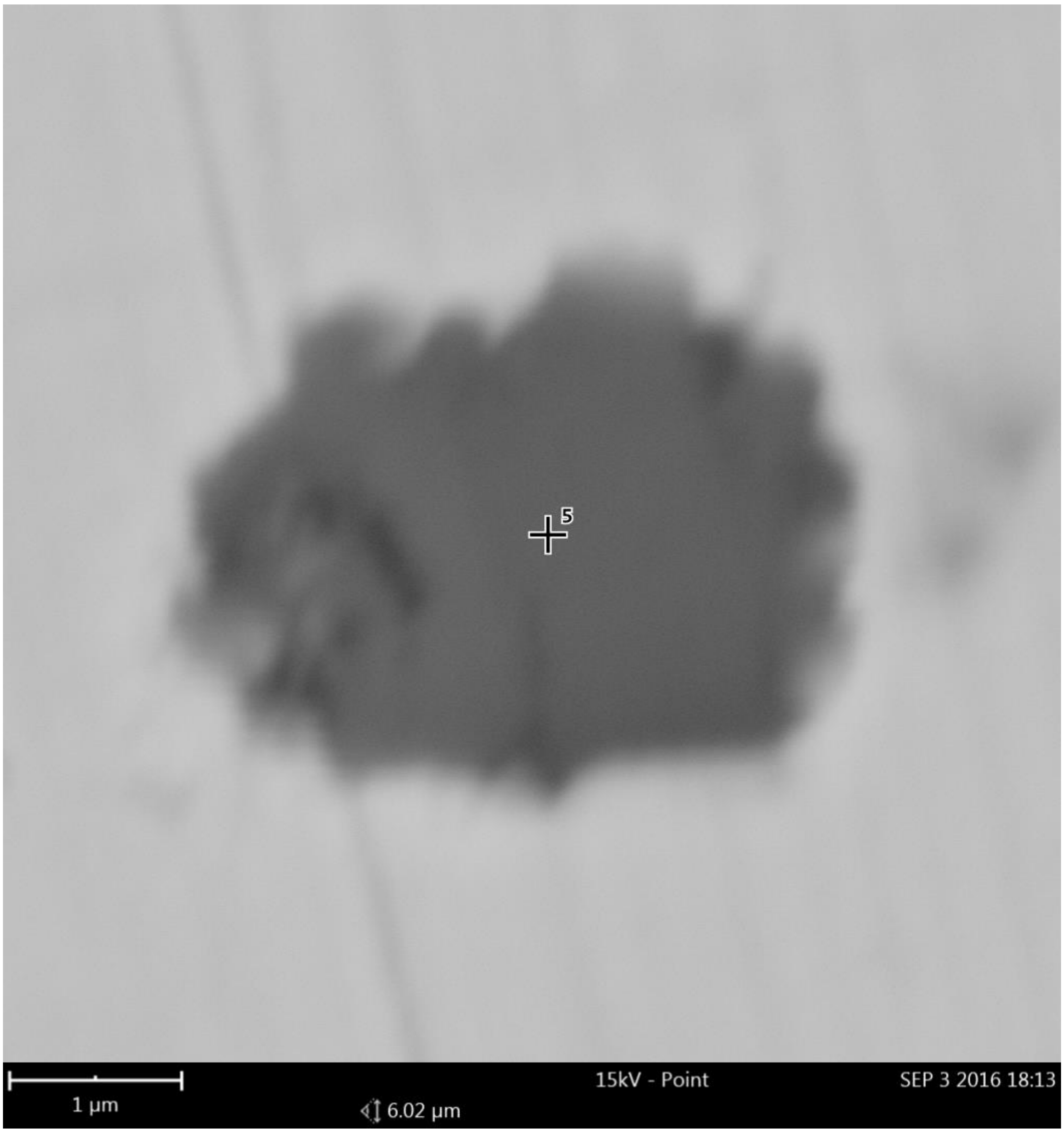

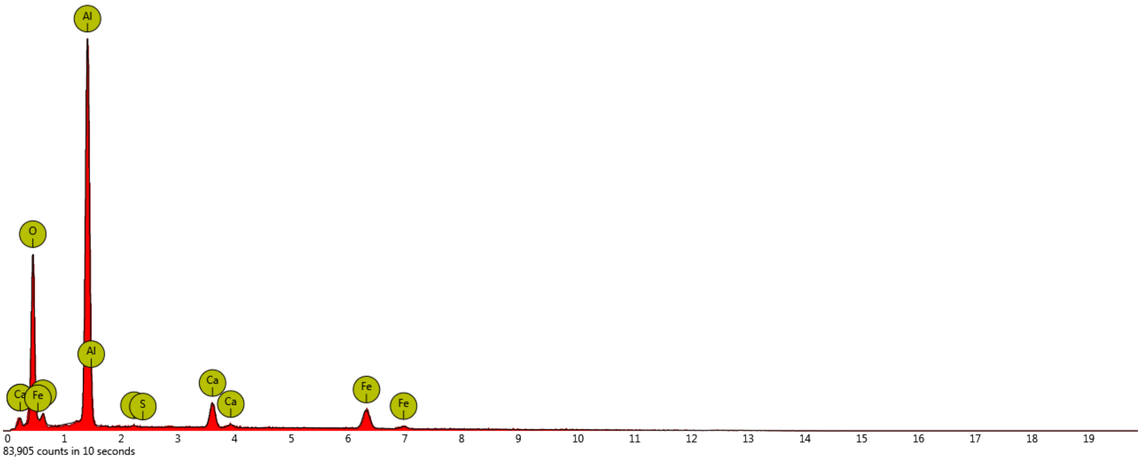

Disabled elements: As, B, Ba, Br, C, Cs, Dy, Er, F, Ga, Hg, La, Lu, Pb, Pm, Pt, Rb, Sb, Sr, Te, Tm, Yb, Zr

Element Number	Element Symbol	Element Name	Weight Concentration	Error
13	Al	Aluminium	40.6	0.1
8	O	Oxygen	42.4	0.3
20	Ca	Calcium	4.5	0.2
26	Fe	Iron	12.2	0.2
16	S	Sulfur	0.2	0.9

Image 2

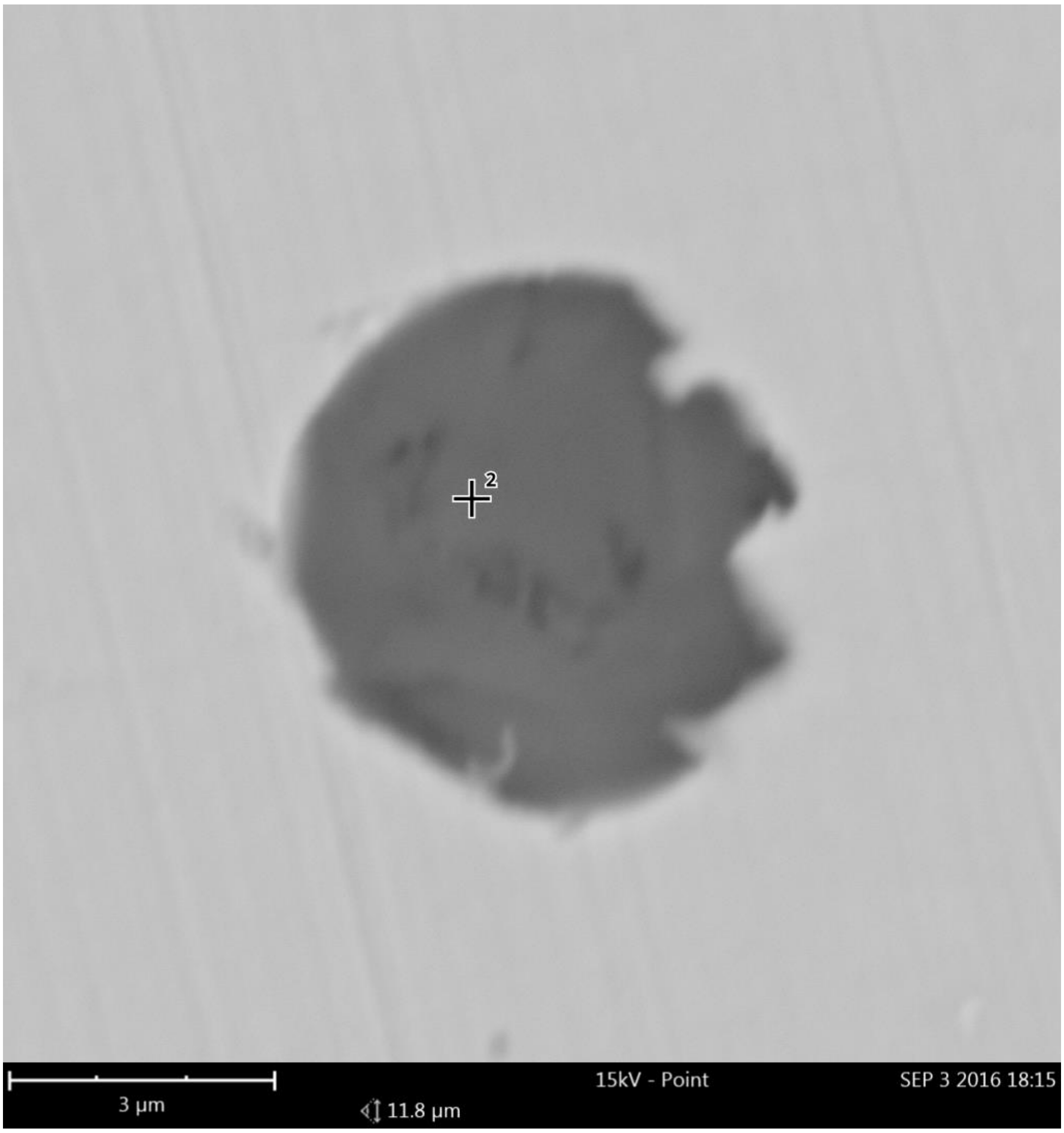

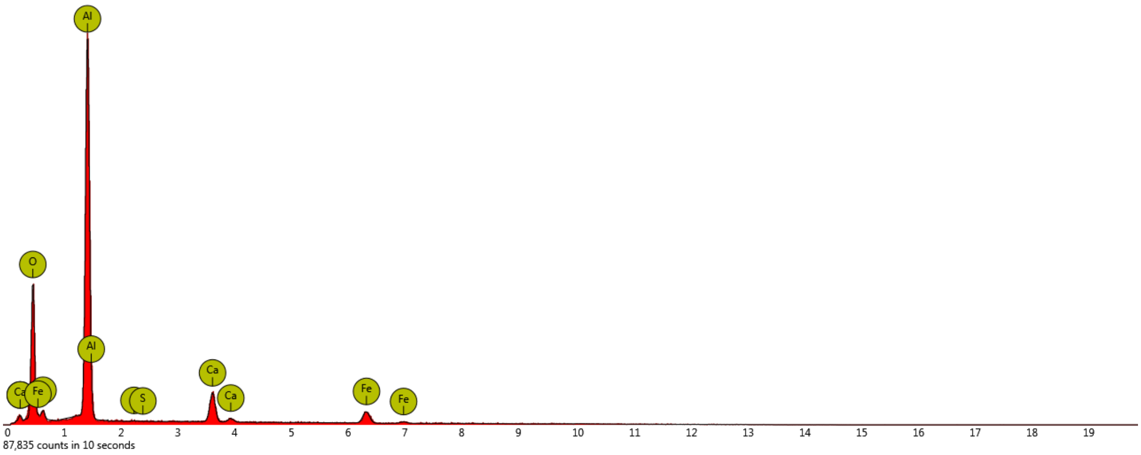

Disabled elements: As, B, Ba, Br, C, Cs, Dy, Er, F, Ga, Hg, La, Lu, Pb, Pm, Pt, Rb, Sb, Sr, Te, Tm, Yb, Zr

Element Number	Element Symbol	Element Name	Weight Concentration	Error
13	Al	Aluminium	43.4	0.1
8	O	Oxygen	41.6	0.4
20	Ca	Calcium	6.1	0.2
26	Fe	Iron	8.7	0.2
16	S	Sulfur	0.1	1.0

5. map

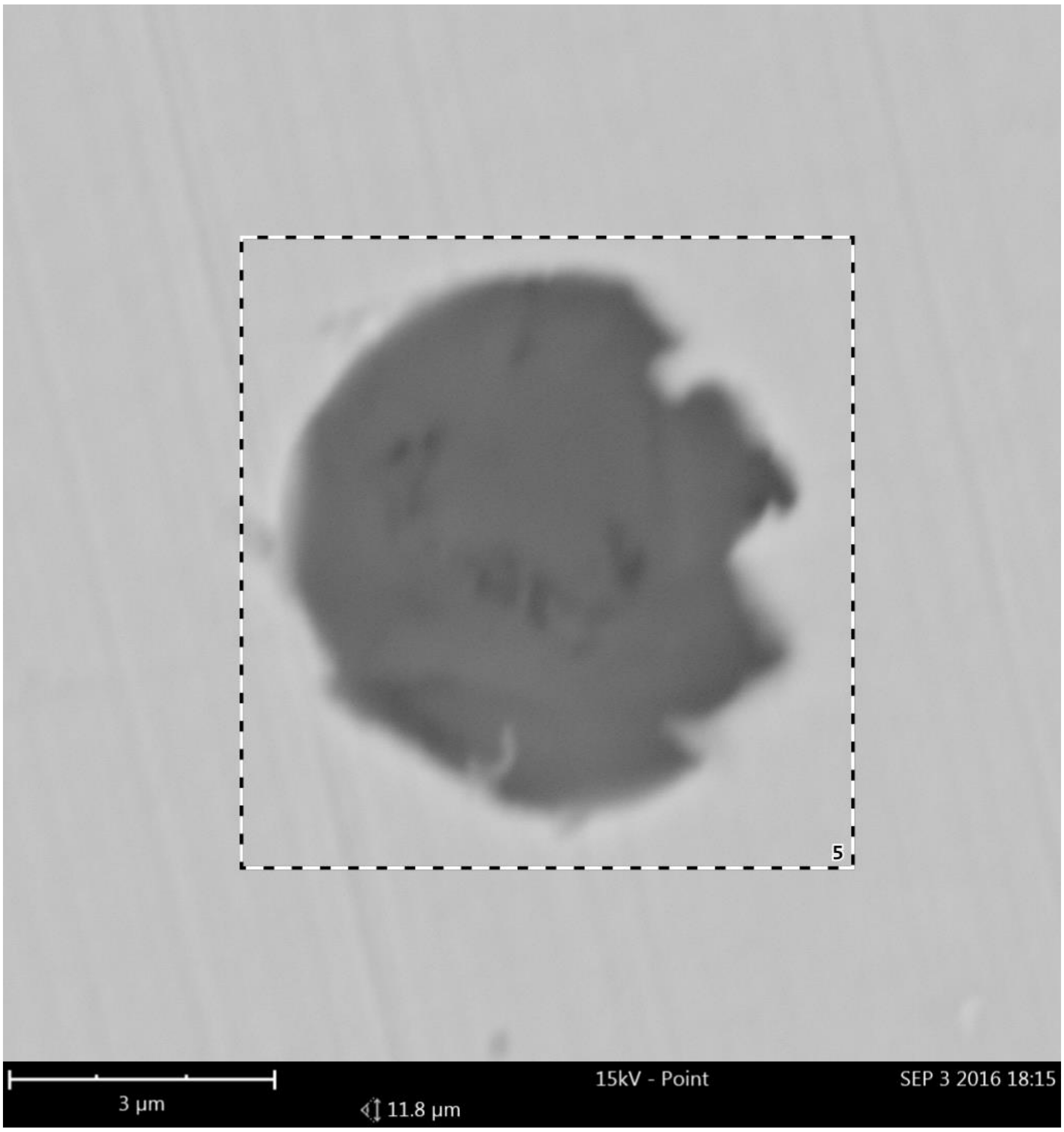

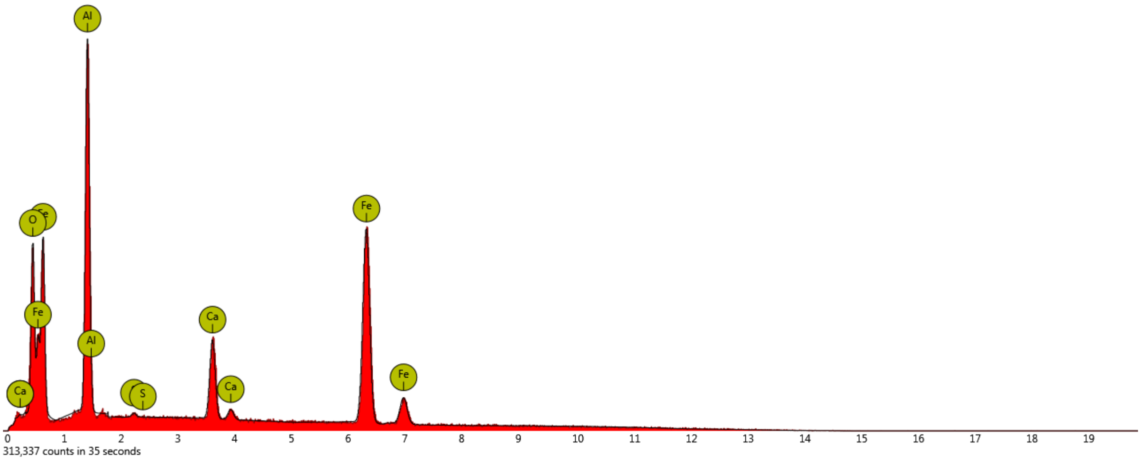

Disabled elements: As, B, Ba, Br, C, Cs, Dy, Er, F, Ga, Hg, La, Lu, Pb, Pm, Pt, Rb, Sb, Sr, Te, Tm, Yb, Zr

**Element Number**	**Element Symbol**	**Element Name**	**Weight Concentration**	**Error**
13	Al	Aluminium	23.0	0.1
26	Fe	Iron	52.9	0.3
8	O	Oxygen	17.8	0.1
20	Ca	Calcium	6.1	0.4

16	S	Sulfur	0.2	0.9

Cut out of map

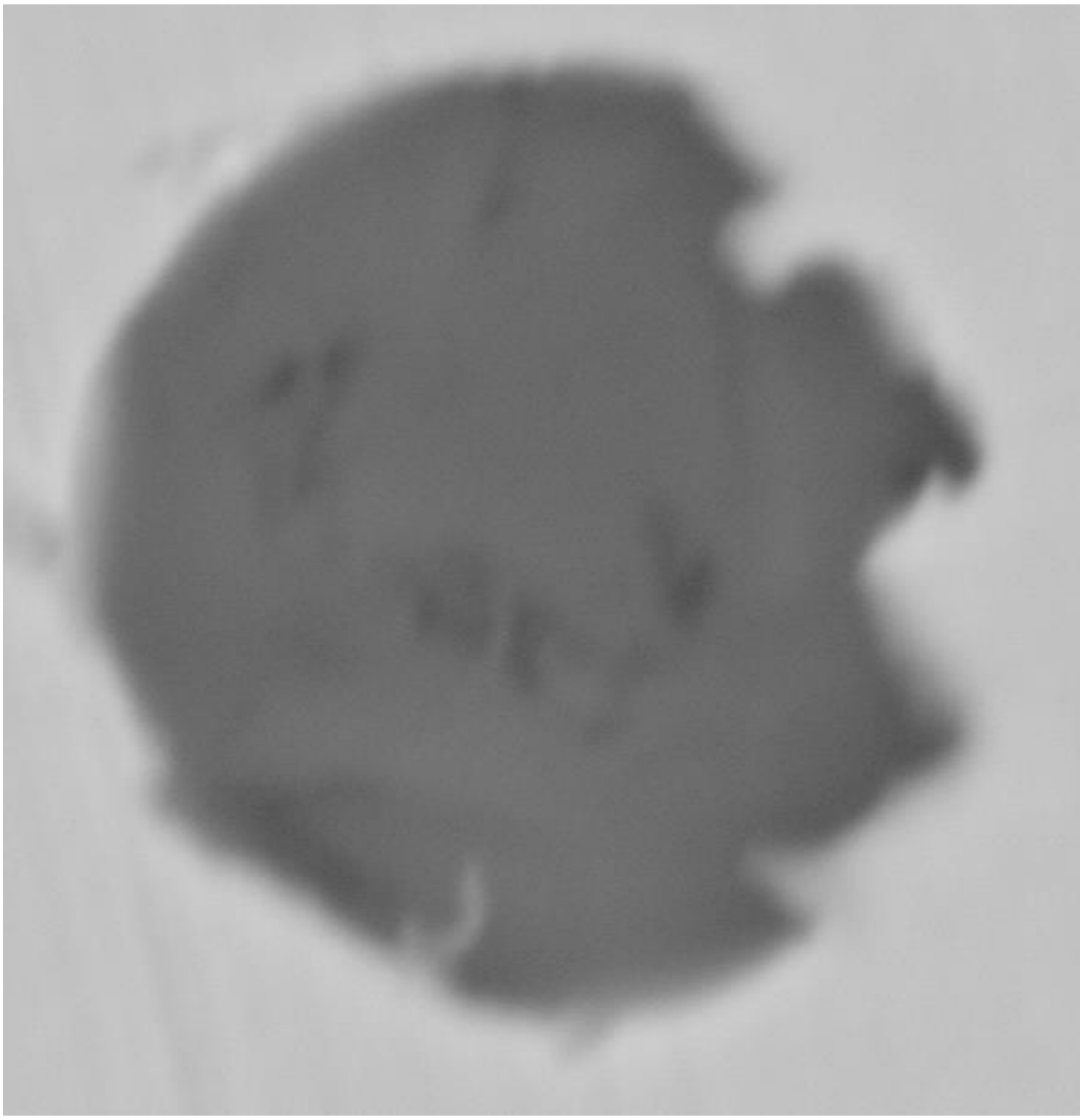

Map: Iron (resolution: 31x32 pixels)

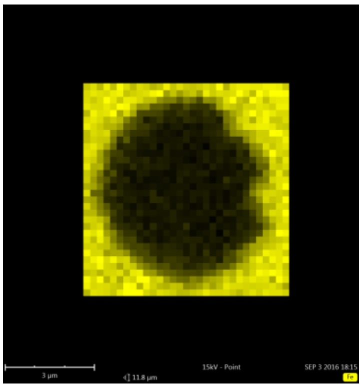

Map: Aluminium (resolution: 31x32 pixels)

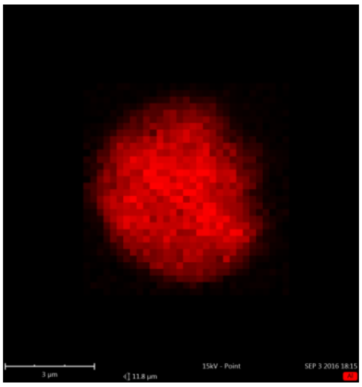

Map: Oxygen (resolution: 31x32 pixels)

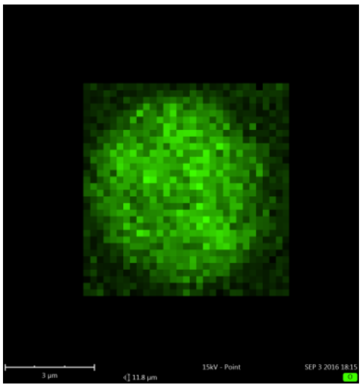

Map: Calcium (resolution: 31x32 pixels)

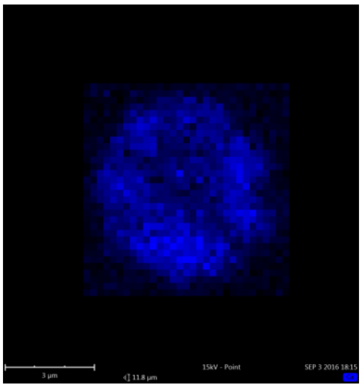

Map: Sulfur (resolution: 31x32 pixels)

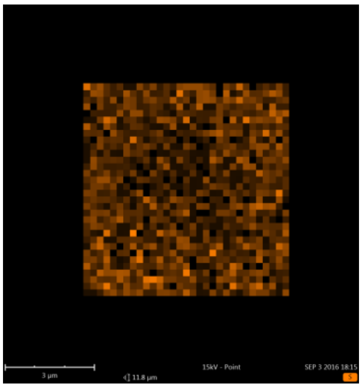

Combined map

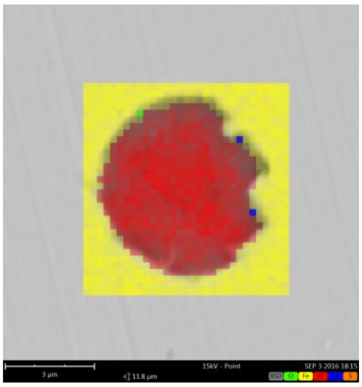

Image 3

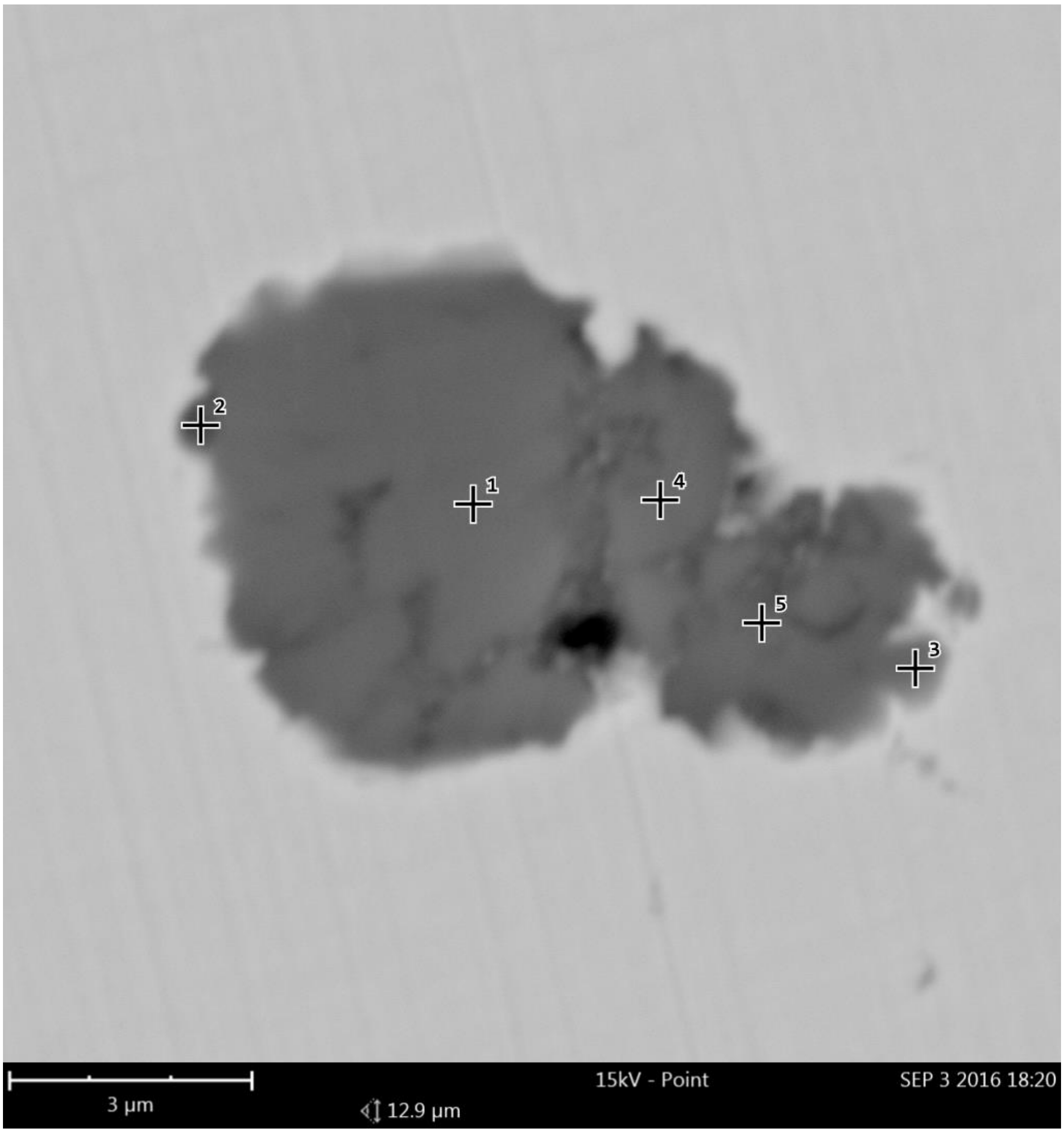

1. spot

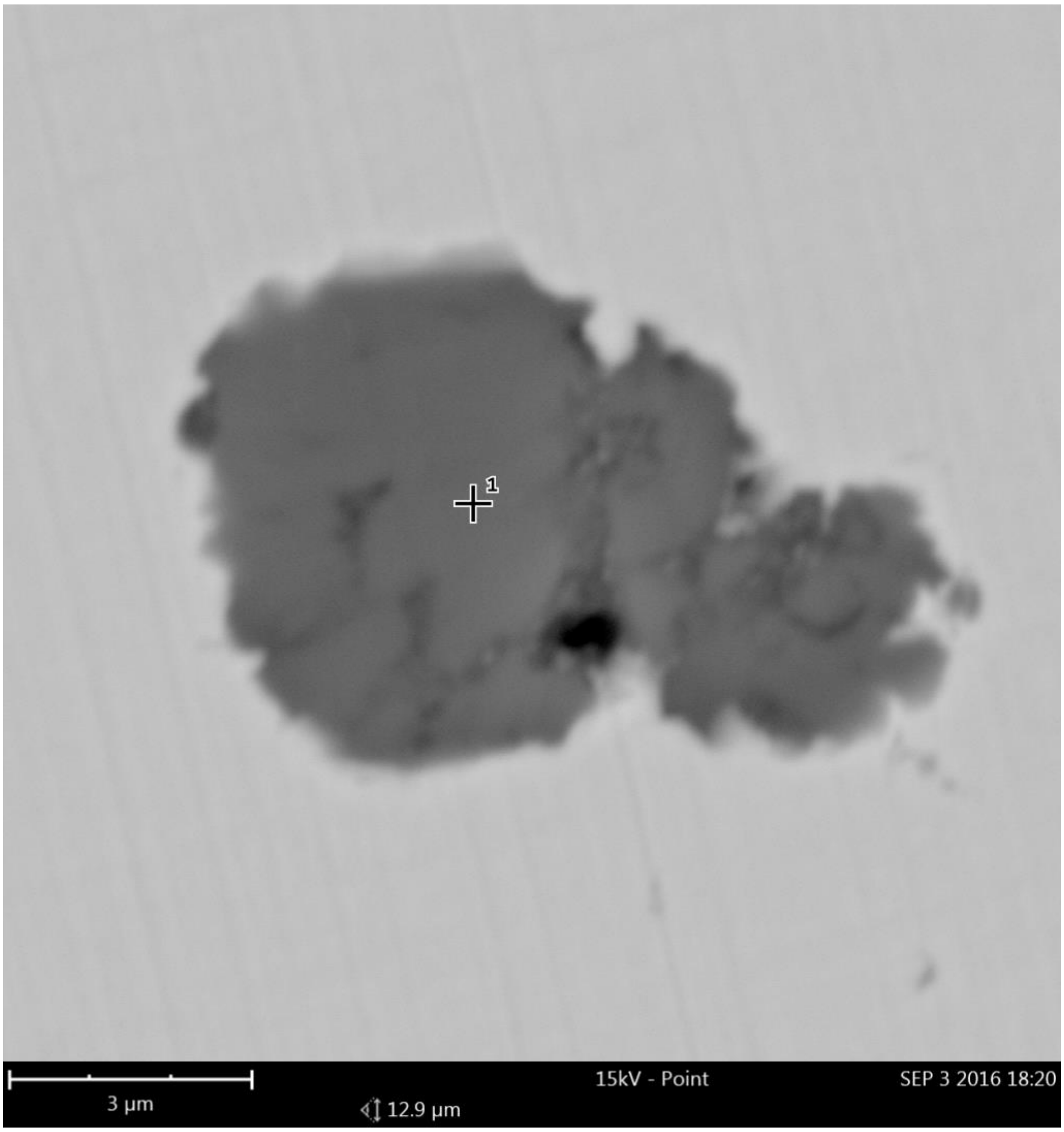

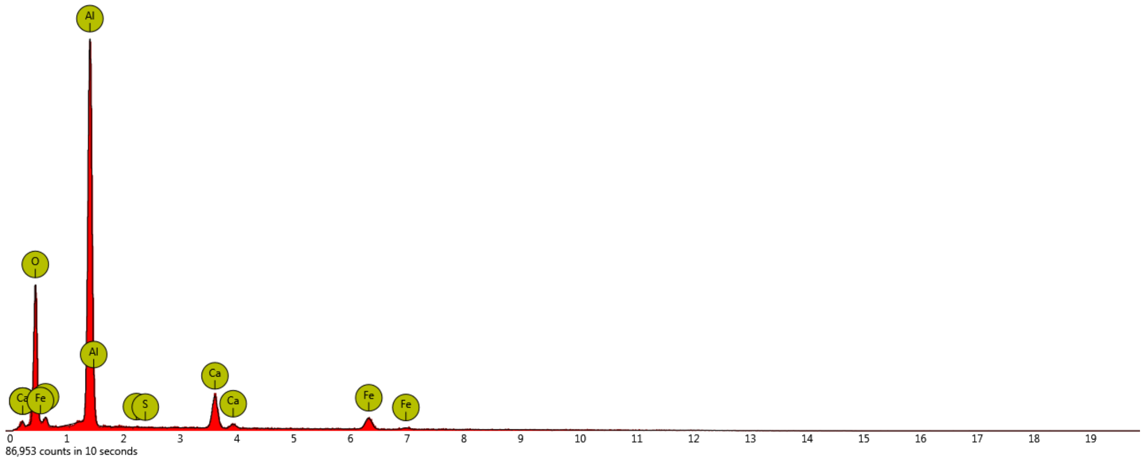

Disabled elements: As, B, Ba, Br, C, Cs, Dy, Er, F, Ga, Hg, La, Lu, Pb, Pm, Pt, Rb, Sb, Sr, Te, Tm, Yb, Zr

Element Number	Element Symbol	Element Name	Weight Concentration	Error
13	Al	Aluminium	42.7	0.1
8	O	Oxygen	42.5	0.4
20	Ca	Calcium	6.6	0.2
26	Fe	Iron	8.1	0.3

16	S	Sulfur	0.1	1.0

Image 4

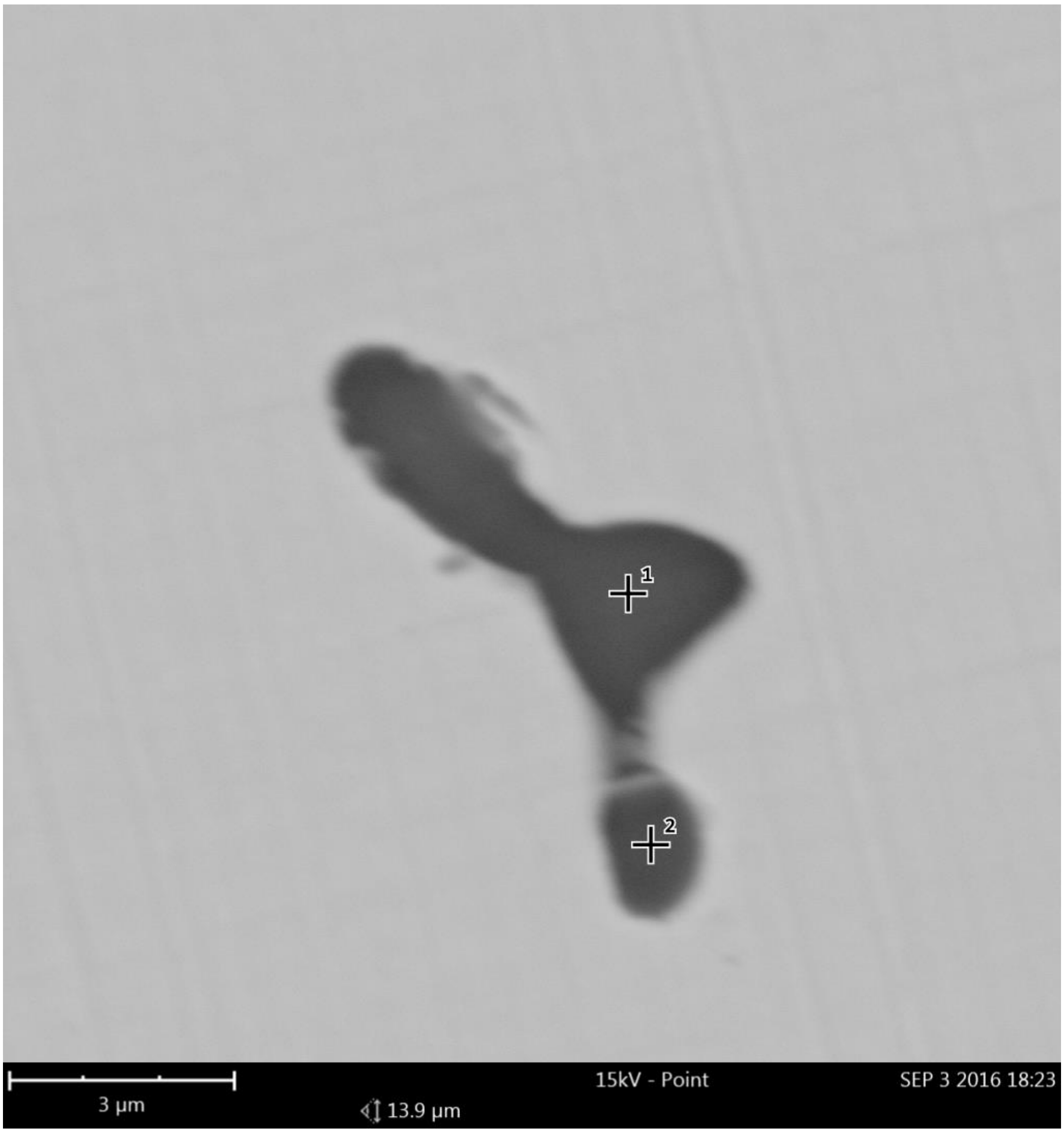

1. spot

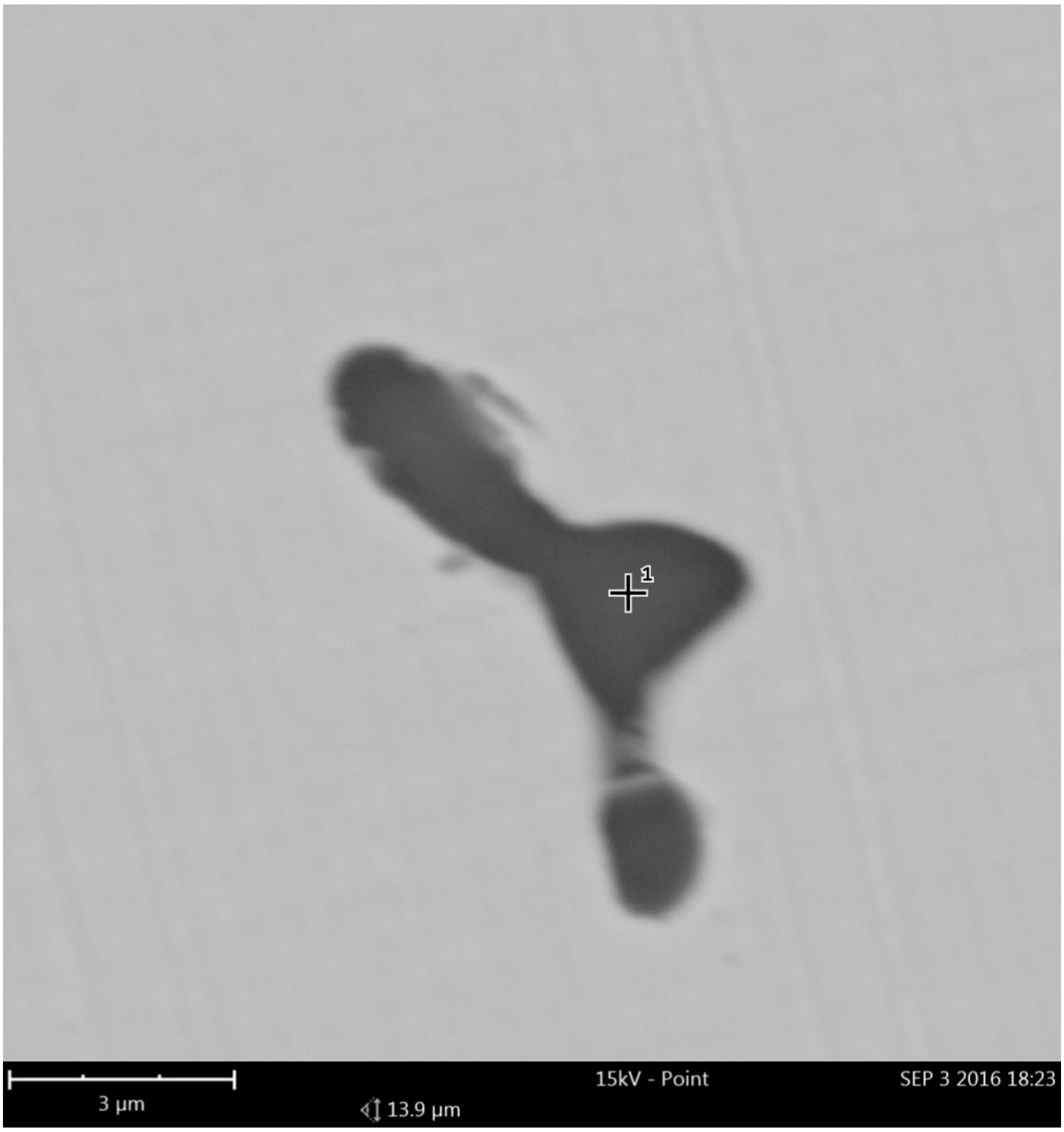

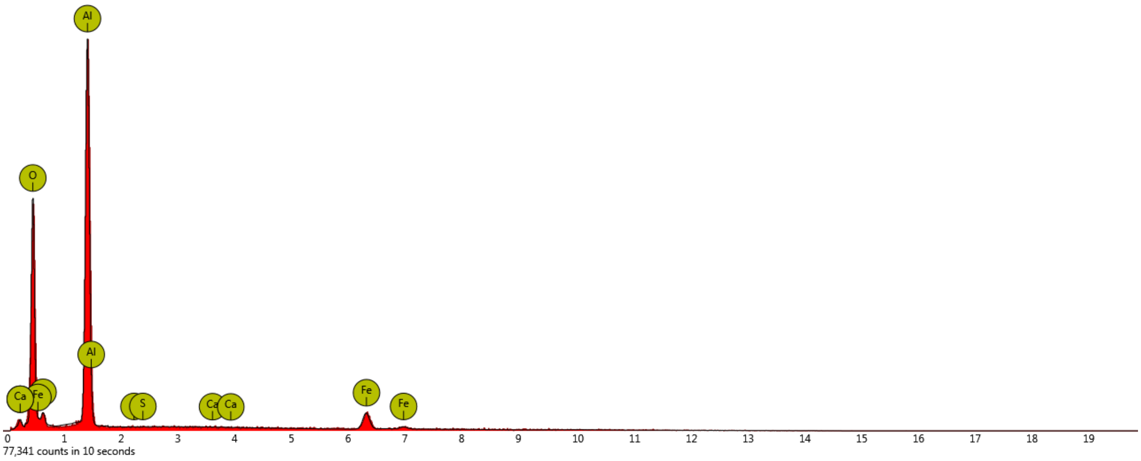

Disabled elements: As, B, Ba, Br, C, Cs, Dy, Er, F, Ga, Hg, La, Lu, Pb, Pm, Pt, Rb, Sb, Sr, Te, Tm, Yb, Zr

Element Number	Element Symbol	Element Name	Weight Concentration	Error
13	Al	Aluminium	41.8	0.2
8	O	Oxygen	47.2	0.7
26	Fe	Iron	11.1	0.4
16	S	Sulfur	0.0	1.1

20	Ca	Calcium	0.0	1.1

2. spot

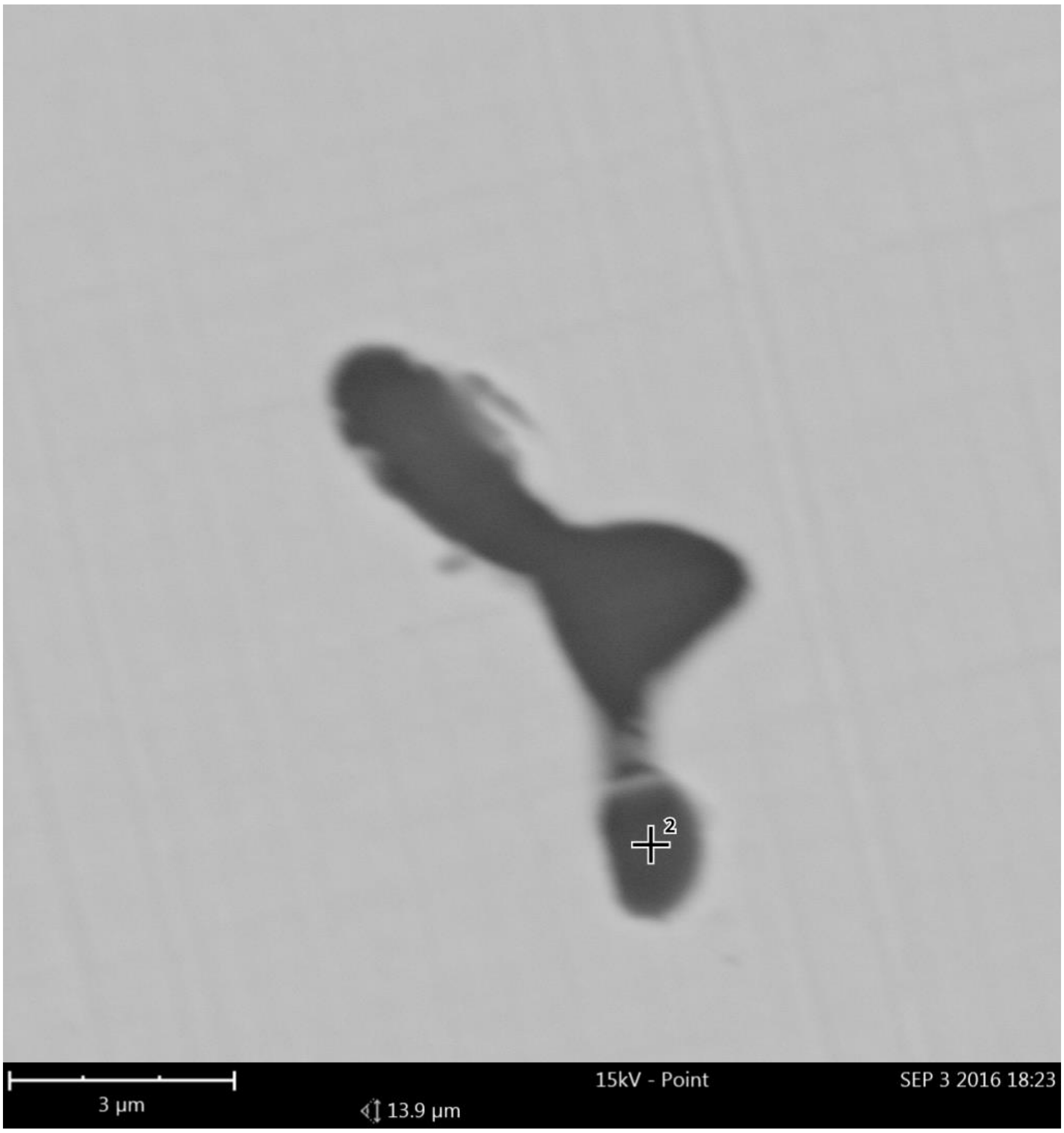

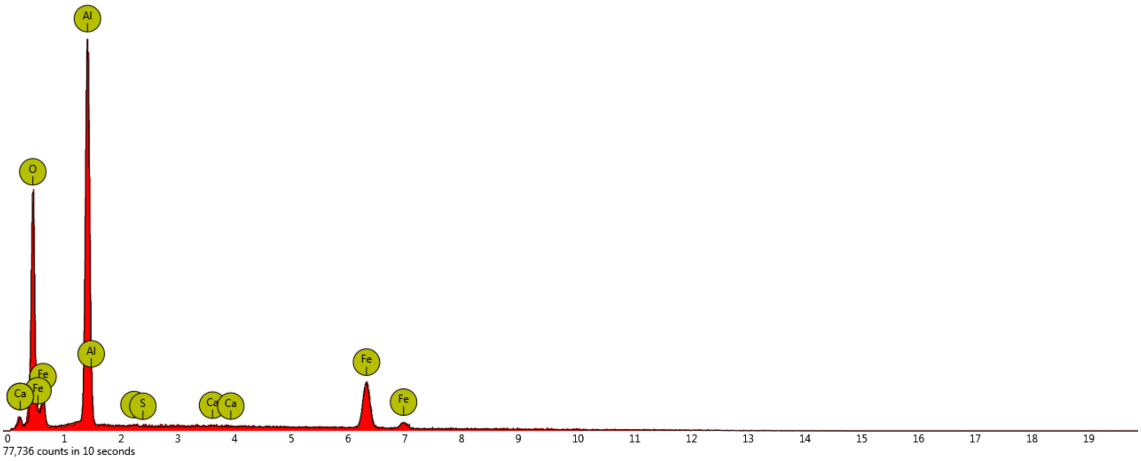

Disabled elements: As, B, Ba, Br, C, Cs, Dy, Er, F, Ga, Hg, La, Lu, Pb, Pm, Pt, Rb, Sb, Sr, Te, Tm, Yb, Zr

Element Number	Element Symbol	Element Name	Weight Concentration	Error

13	Al	Aluminium	37.0	0.1
8	O	Oxygen	38.1	0.4
26	Fe	Iron	24.6	0.2
16	S	Sulfur	0.2	2.4
20	Ca	Calcium	0.1	1.1

Image 5

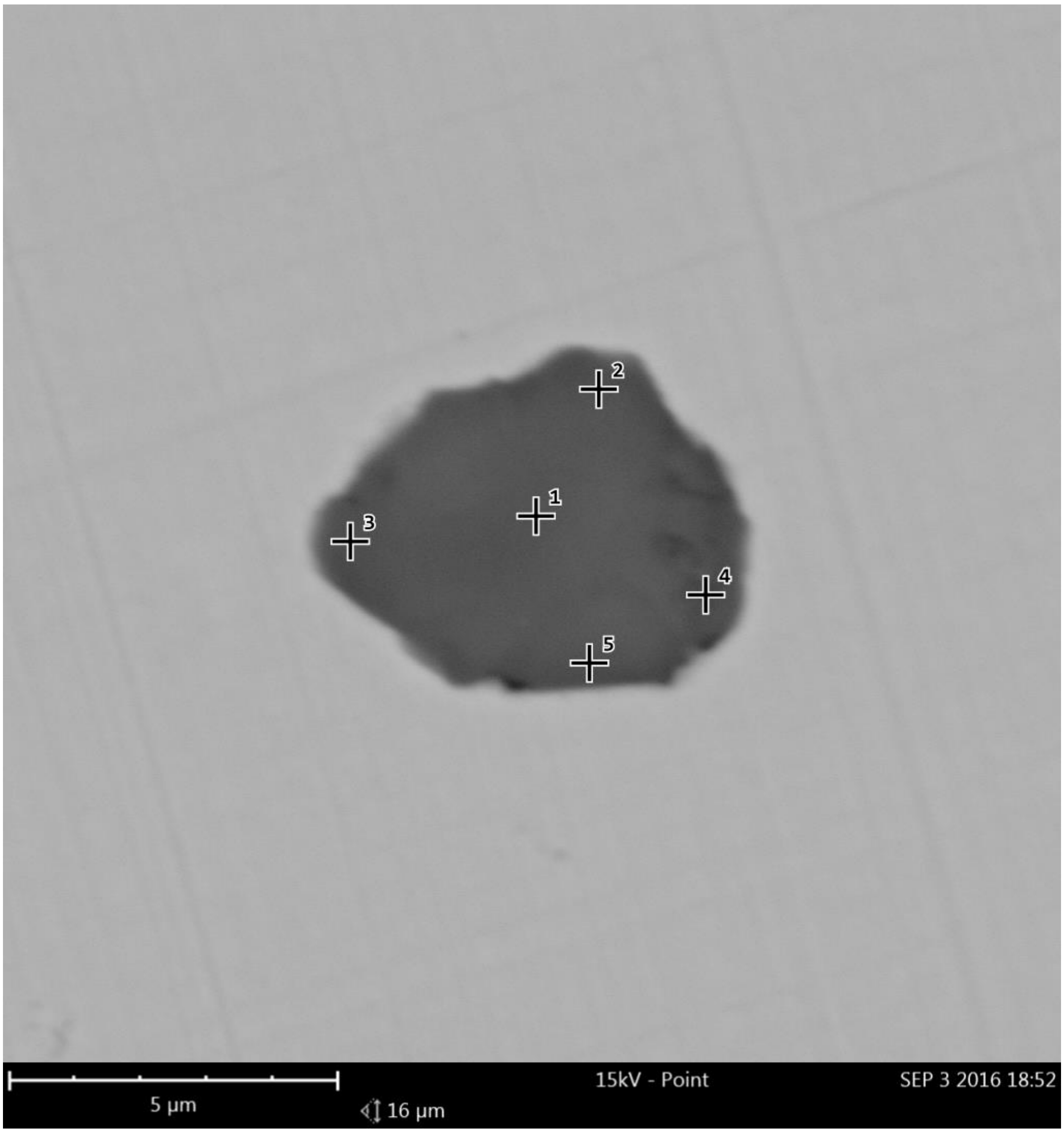

1. spot

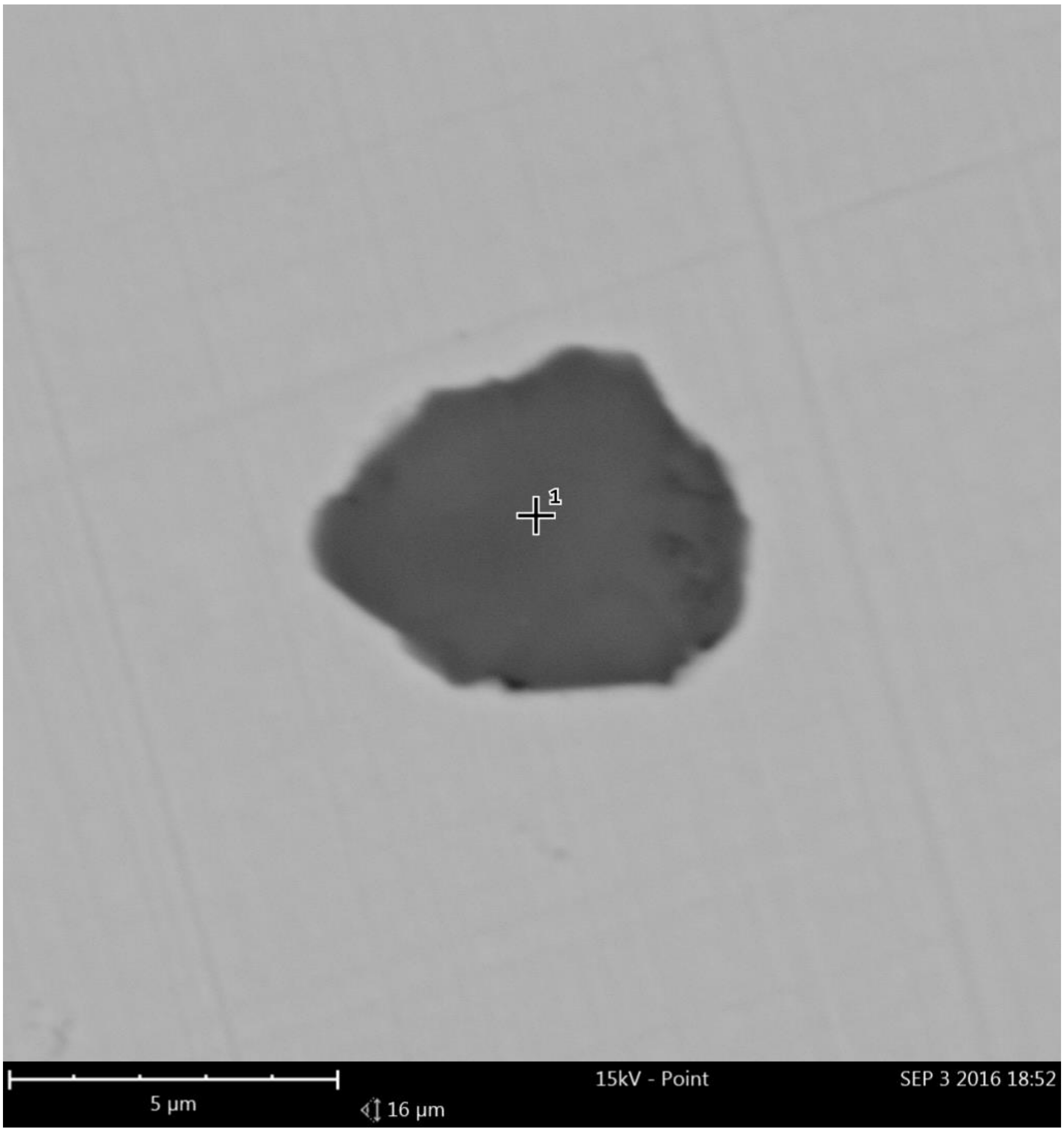

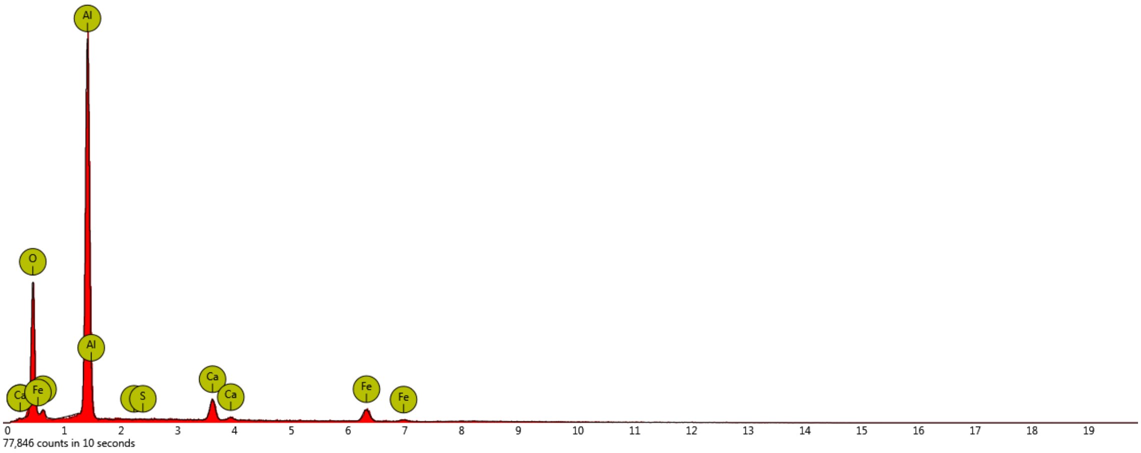

Disabled elements: As, B, Ba, Br, C, Cs, Dy, Er, F, Ga, Hg, La, Lu, Pb, Pm, Pt, Rb, Sb, Sr, Te, Tm, Yb, Zr

**Element Number**	**Element Symbol**	**Element Name**	**Weight Concentration**	**Error**
13	Al	Aluminium	44.9	0.1
8	O	Oxygen	41.4	0.4
20	Ca	Calcium	4.6	0.3
26	Fe	Iron	9.2	0.4

16	S	Sulfur	0.0	1.4

2. spot

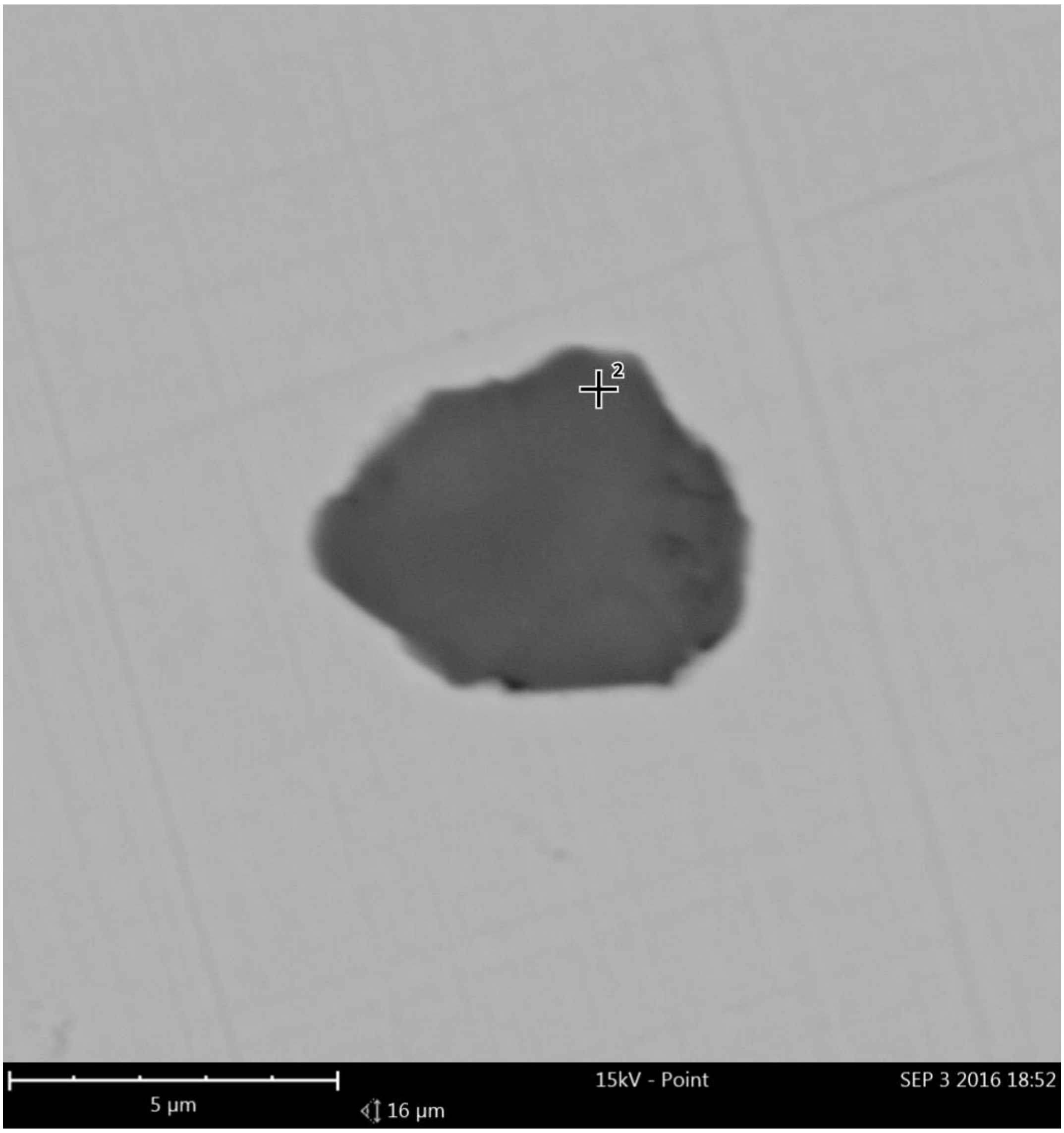

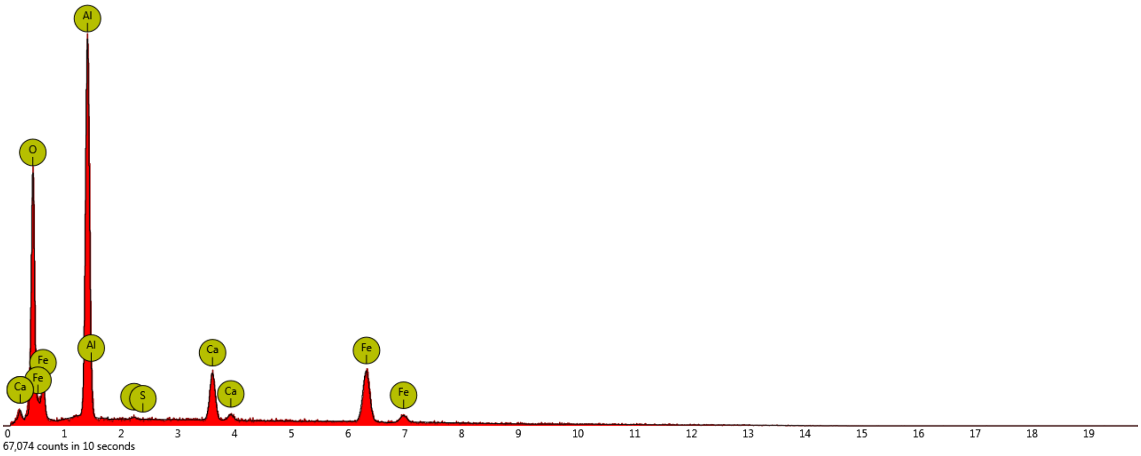

Disabled elements: As, B, Ba, Br, C, Cs, Dy, Er, F, Ga, Hg, La, Lu, Pb, Pm, Pt, Rb, Sb, Sr, Te, Tm, Yb, Zr

Element Number	Element Symbol	Element Name	Weight Concentration	Error

13	Al	Aluminium	30.8	0.1
8	O	Oxygen	40.1	0.4
26	Fe	Iron	23.0	0.2
20	Ca	Calcium	5.9	0.1
16	S	Sulfur	0.2	0.9

3. spot

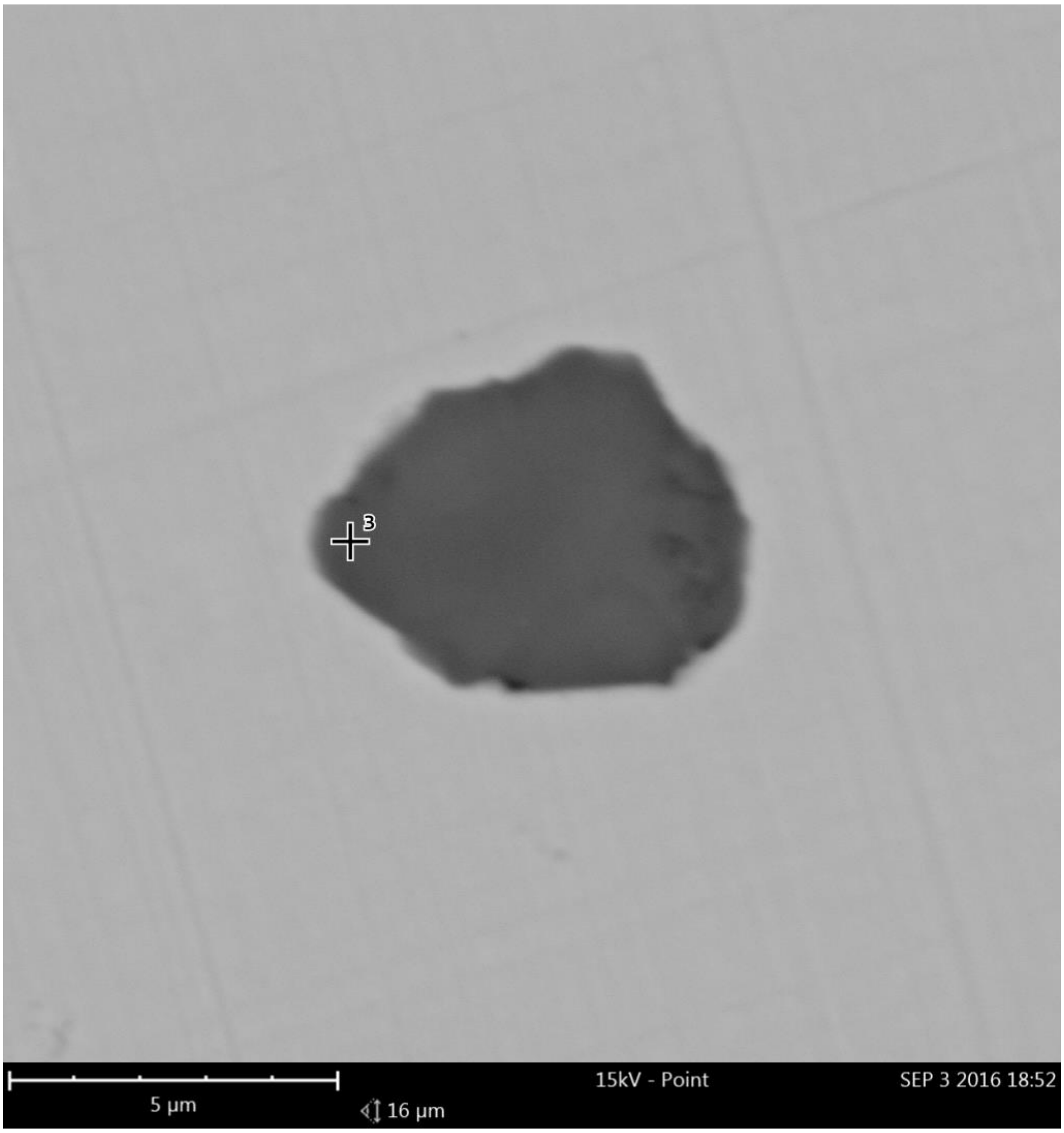

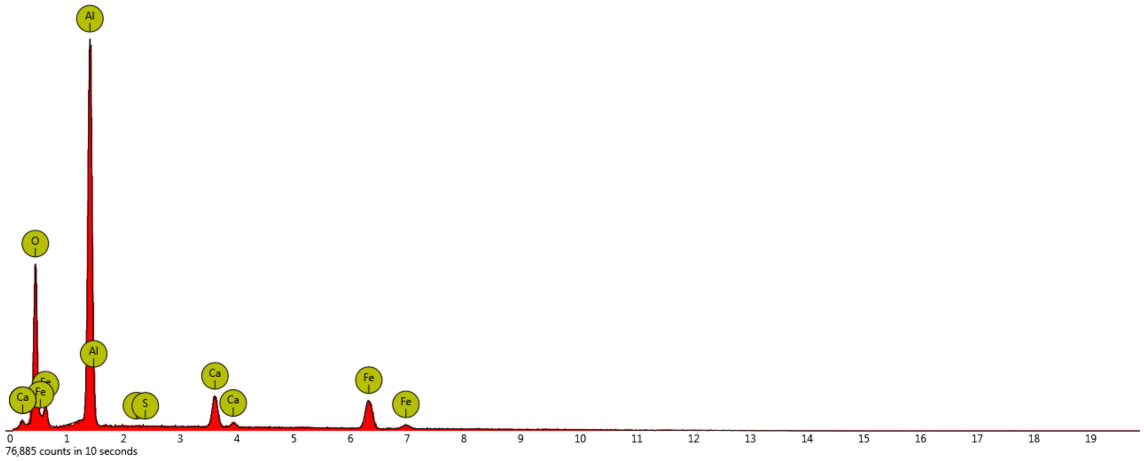

Disabled elements: As, B, Ba, Br, C, Cs, Dy, Er, F, Ga, Hg, La, Lu, Pb, Pm, Pt, Rb, Sb, Sr, Te, Tm, Yb, Zr

Element Number	Element Symbol	Element Name	Weight Concentration	Error
13	Al	Aluminium	39.1	0.1
8	O	Oxygen	38.0	0.4
26	Fe	Iron	17.5	0.3
20	Ca	Calcium	5.4	0.2
16	S	Sulfur	0.0	1.0

4. spot

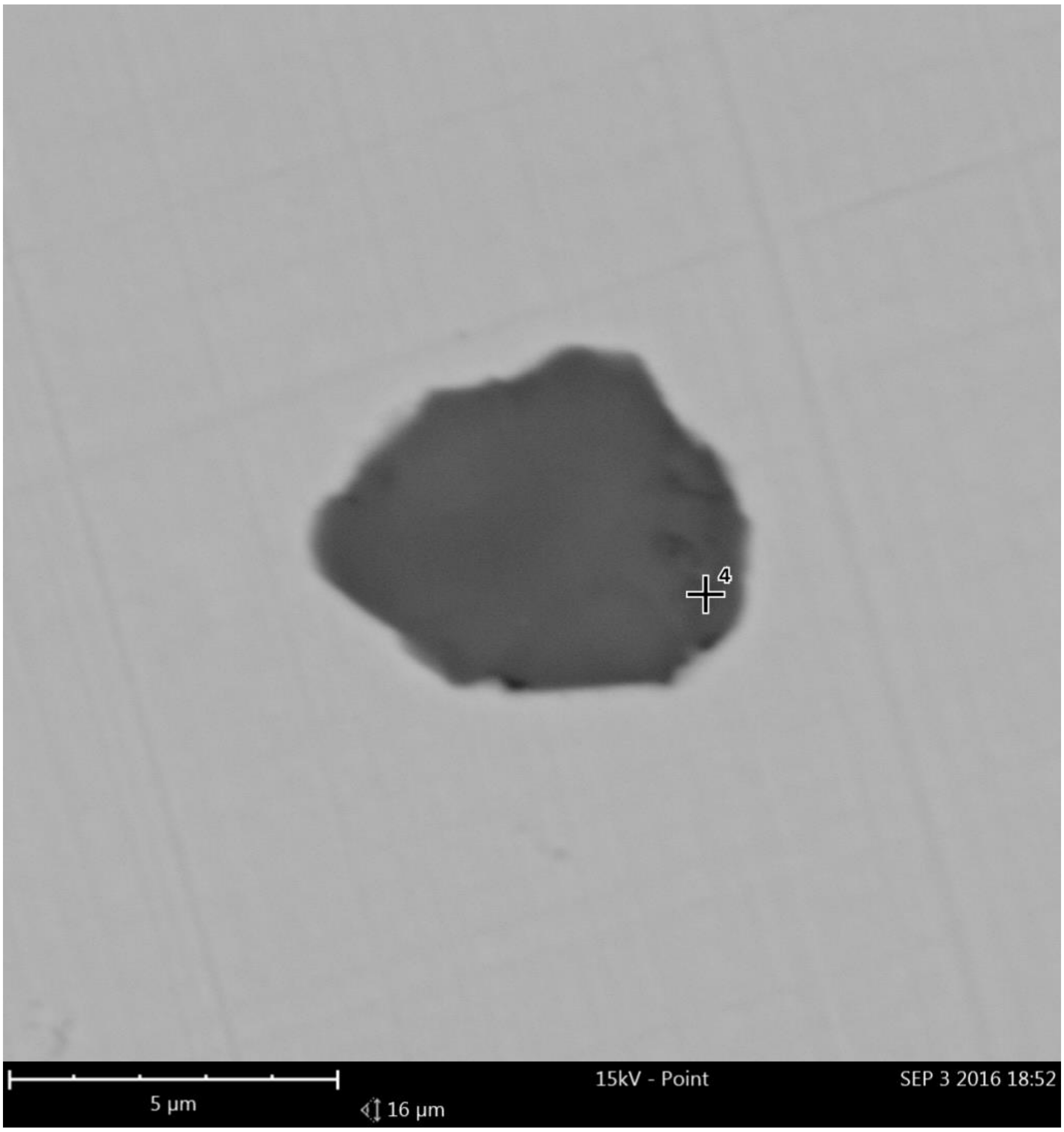

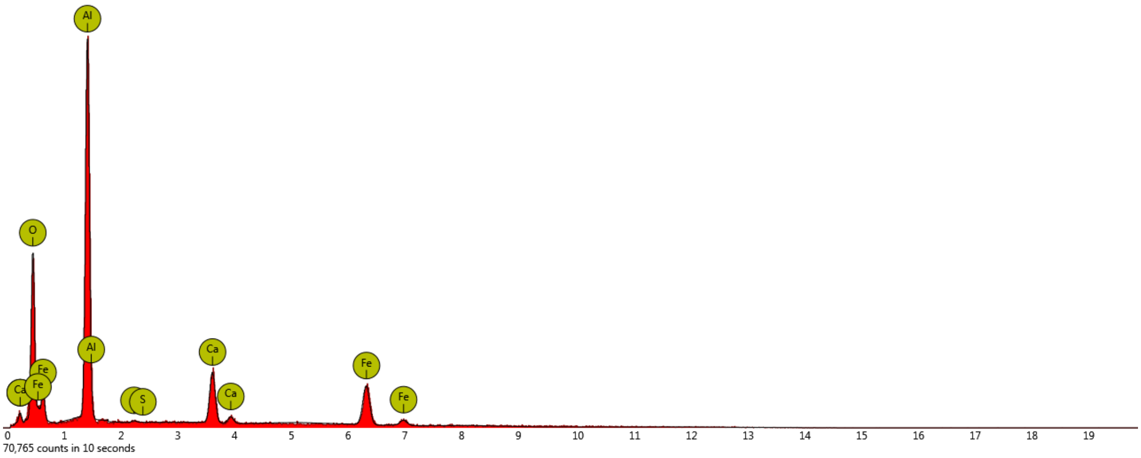

Disabled elements: As, B, Ba, Br, C, Cs, Dy, Er, F, Ga, Hg, La, Lu, Pb, Pm, Pt, Rb, Sb, Sr, Te, Tm, Yb, Zr

Element Number	Element Symbol	Element Name	Weight Concentration	Error
13	Al	Aluminium	35.1	0.1
8	O	Oxygen	36.4	0.4
20	Ca	Calcium	7.6	0.2
26	Fe	Iron	20.8	0.3

16	S	Sulfur	0.2	1.3

5. spot

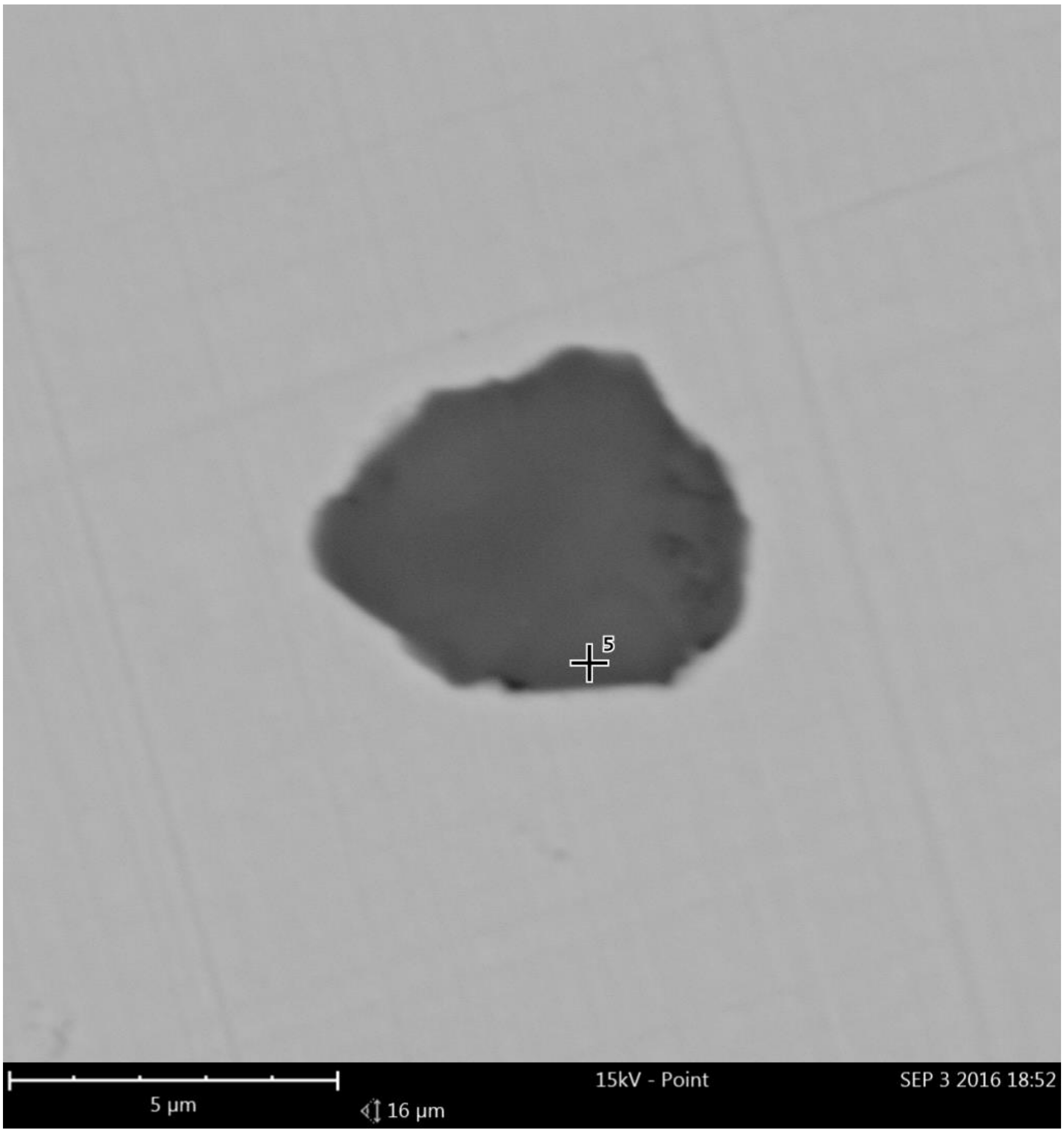

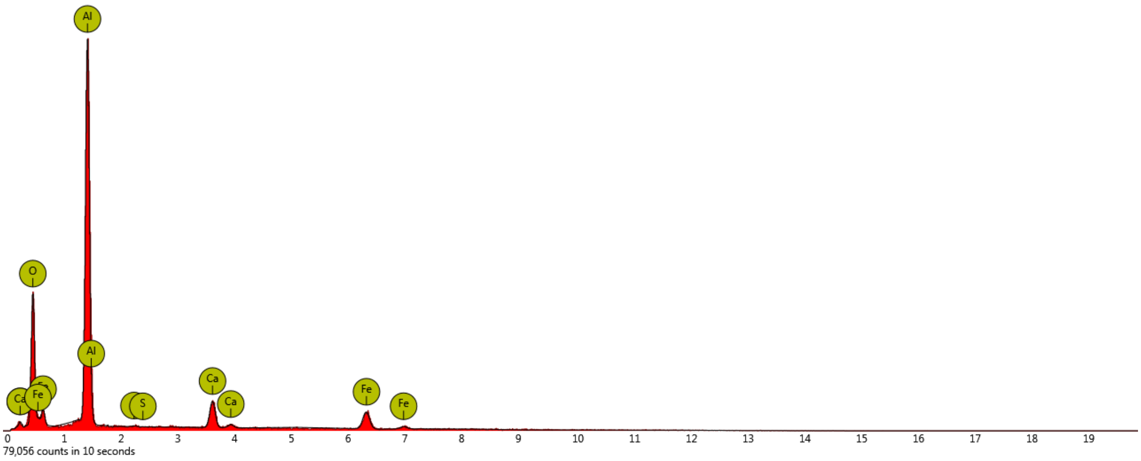

Disabled elements: As, B, Ba, Br, C, Cs, Dy, Er, F, Ga, Hg, La, Lu, Pb, Pm, Pt, Rb, Sb, Sr, Te, Tm, Yb, Zr

Element Number	Element Symbol	Element Name	Weight Concentration	Error

13	Al	Aluminium	43.5	0.1
8	O	Oxygen	38.6	0.3
20	Ca	Calcium	5.4	0.3
26	Fe	Iron	12.3	0.4
16	S	Sulfur	0.1	1.2

Image 6

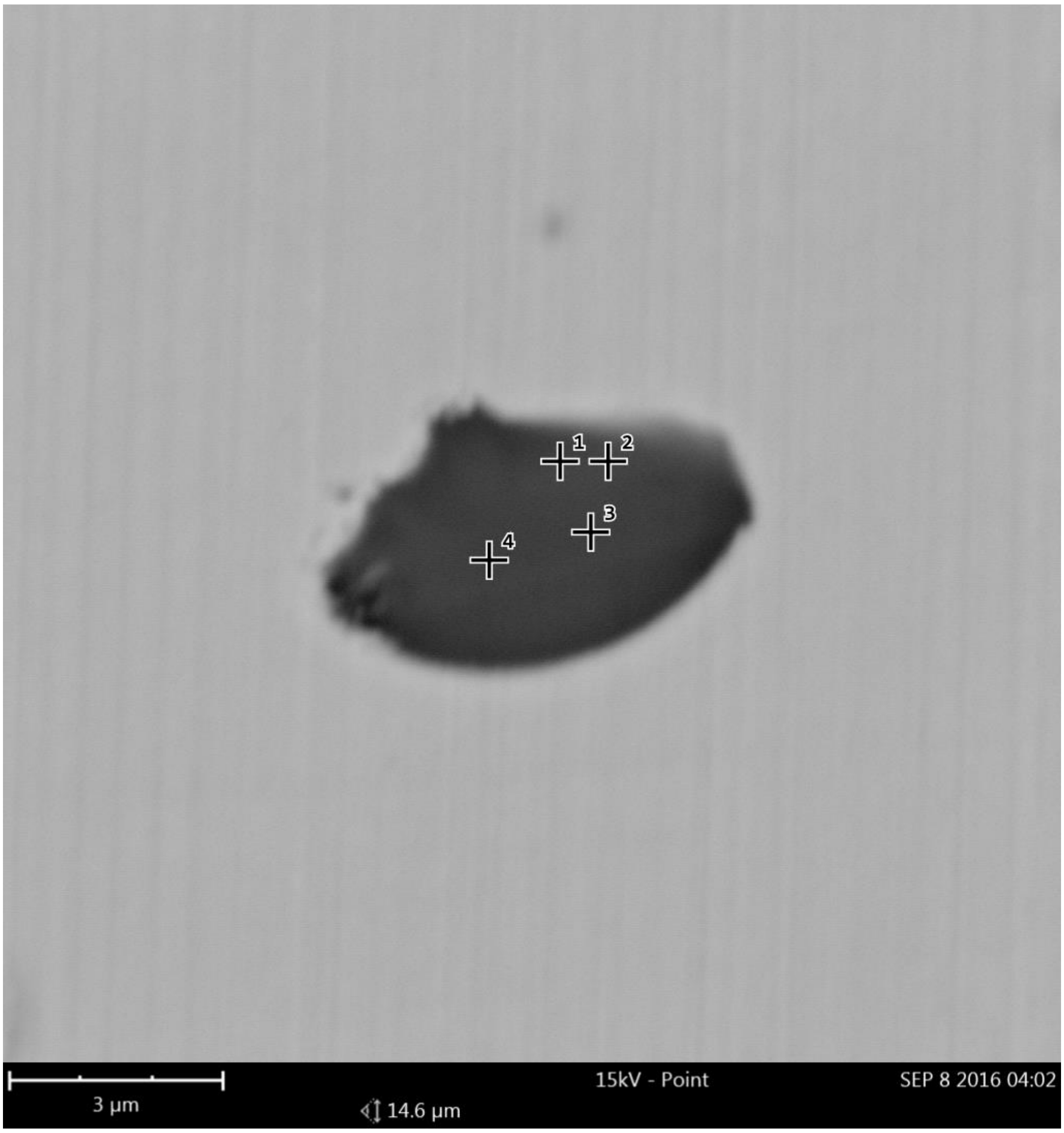

1. spot

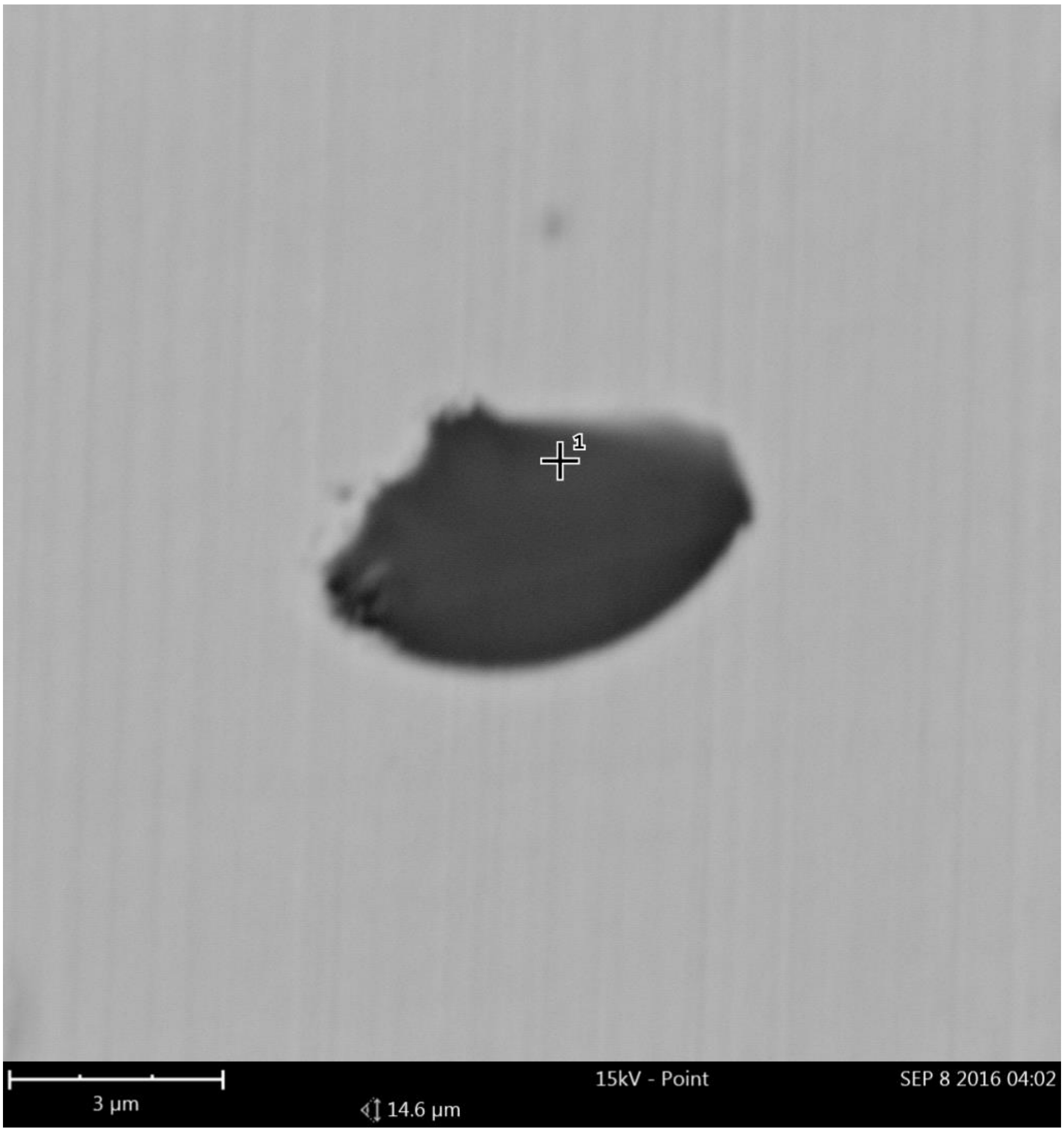

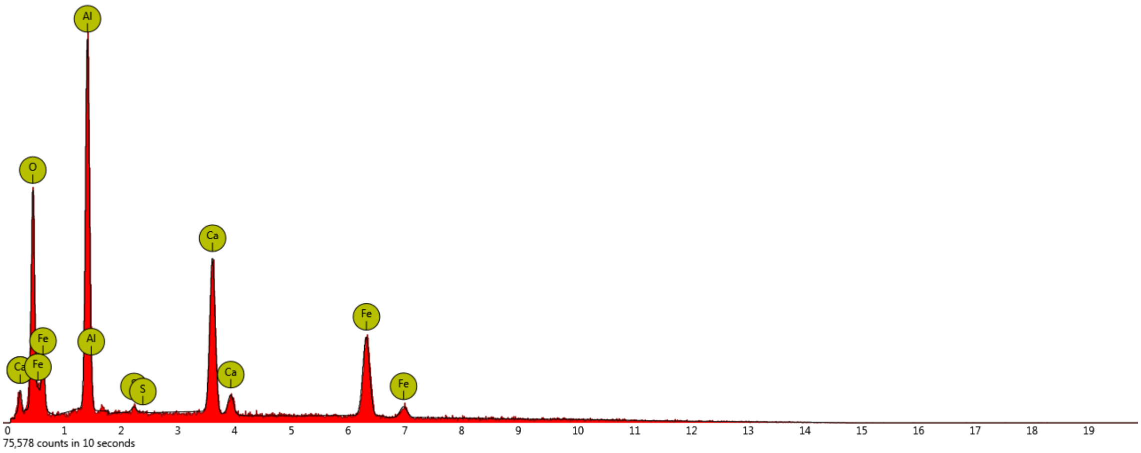

Disabled elements: As, B, Ba, Br, C, Cs, Dy, Er, F, Ga, Hg, La, Lu, Pb, Pm, Pt, Rb, Sb, Si, Sr, Te, Tm, Yb, Zr

Element Number	Element Symbol	Element Name	Weight Concentration	Error
13	Al	Aluminium	23.6	0.1
20	Ca	Calcium	14.3	0.1
8	O	Oxygen	35.1	0.4

26	Fe	Iron	26.5	0.3
16	S	Sulfur	0.4	0.5

2. spot

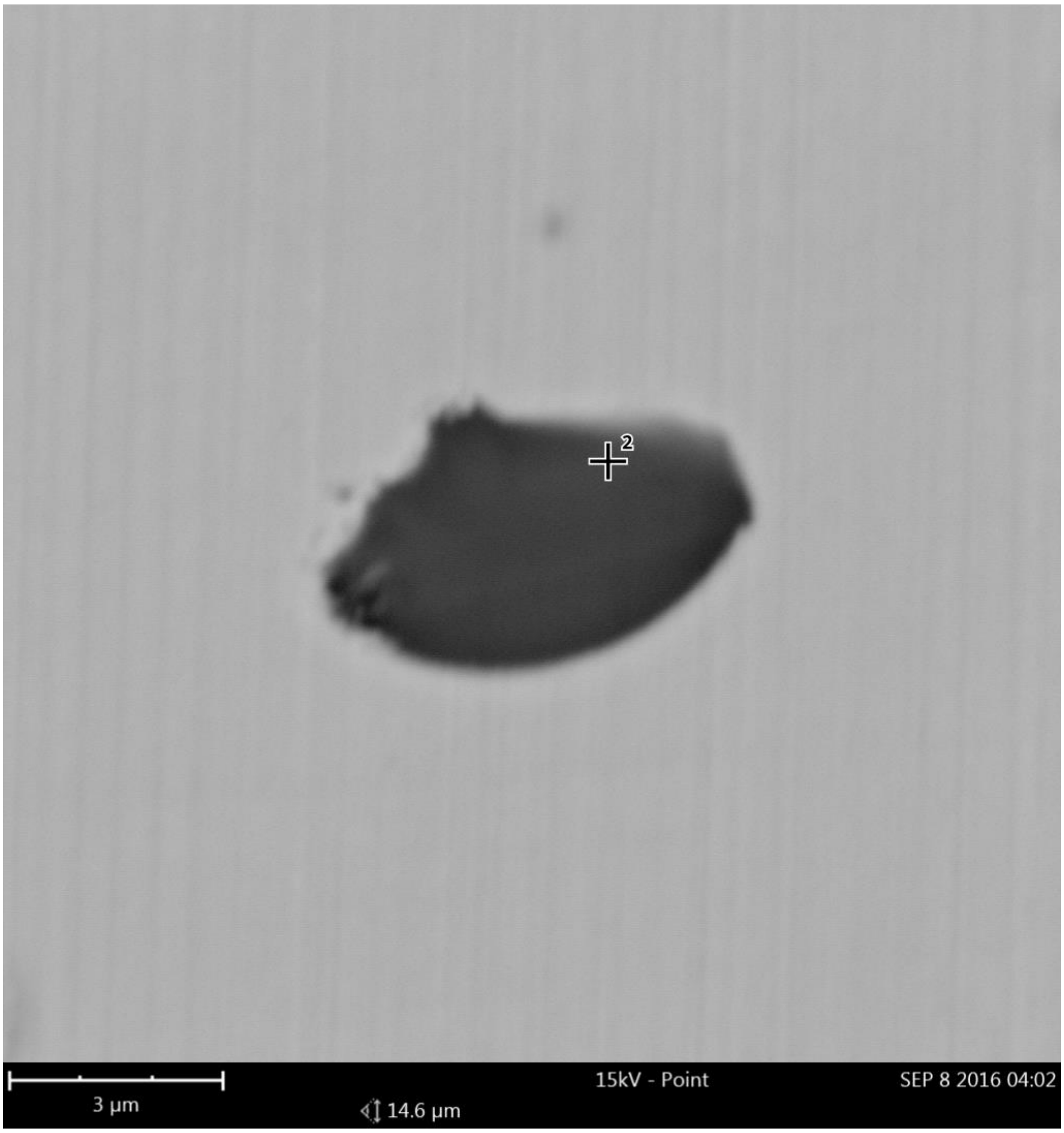

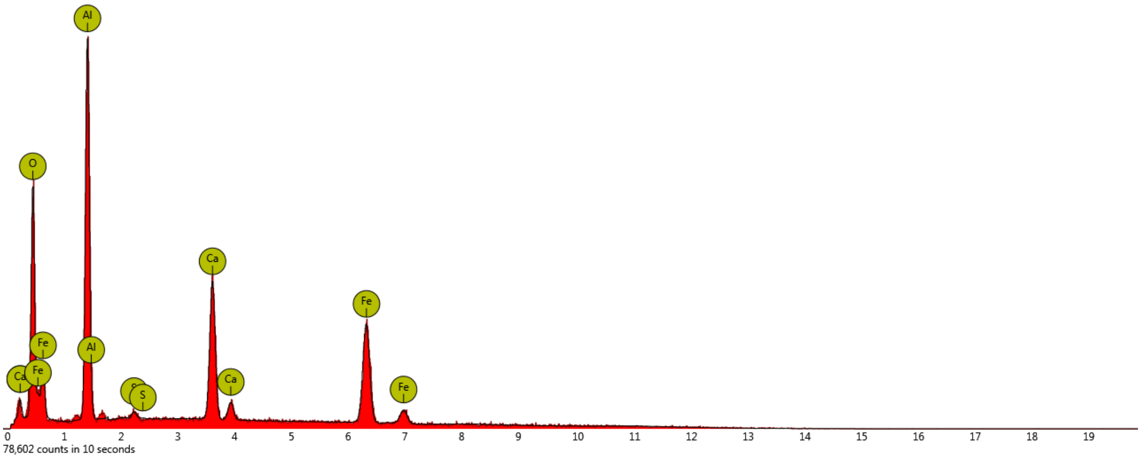

Disabled elements: As, B, Ba, Br, C, Cs, Dy, Er, F, Ga, Hg, La, Lu, Pb, Pm, Pt, Rb, Sb, Si, Sr, Te, Tm, Yb,

Zr

Element Number	Element Symbol	Element Name	Weight Concentration	Error
13	Al	Aluminium	23.7	0.1
8	O	Oxygen	32.5	0.4
20	Ca	Calcium	12.1	0.1
26	Fe	Iron	31.3	0.2
16	S	Sulfur	0.4	1.0

3. spot

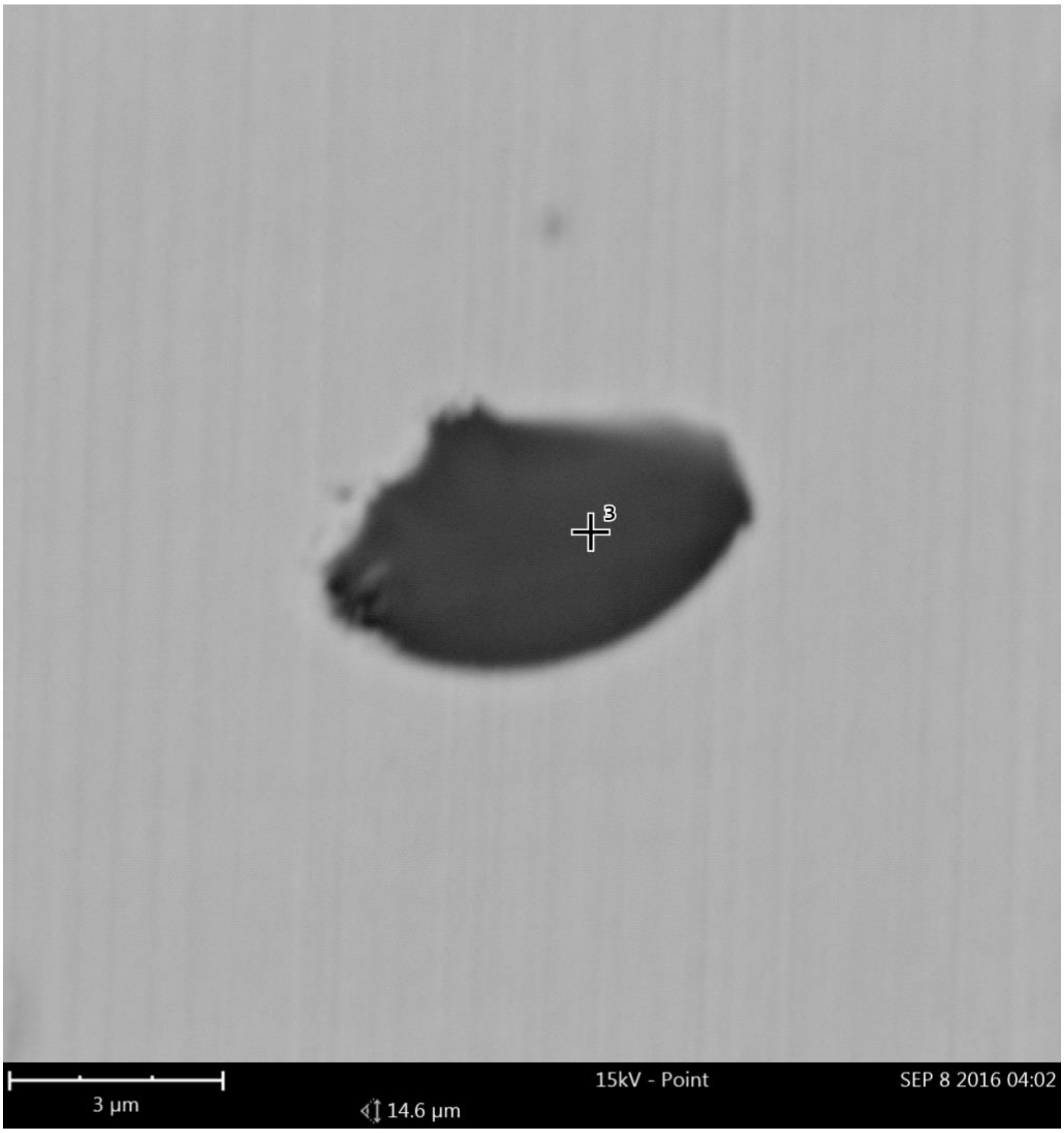

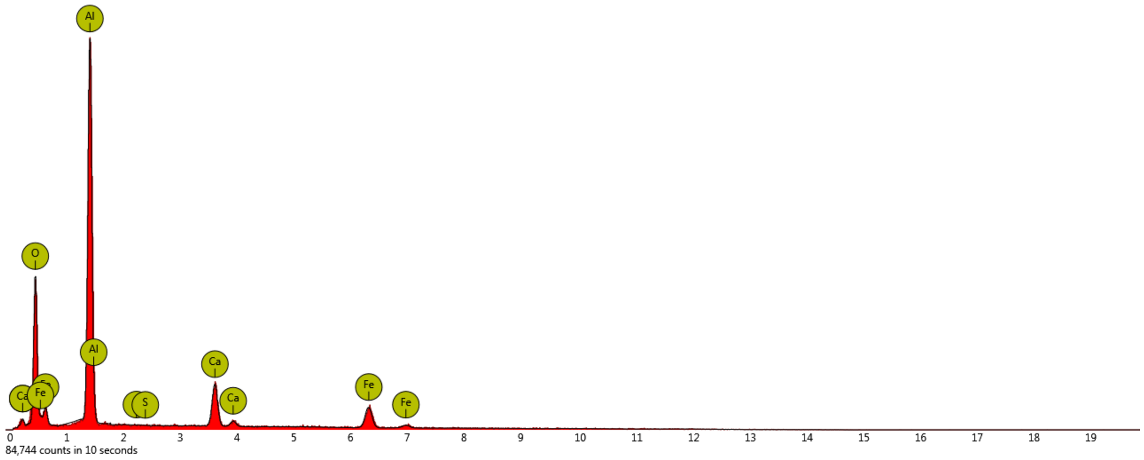

Disabled elements: As, B, Ba, Br, C, Cs, Dy, Er, F, Ga, Hg, La, Lu, Pb, Pm, Pt, Rb, Sb, Si, Sr, Te, Tm, Yb, Zr

**Element Number**	**Element Symbol**	**Element Name**	**Weight Concentration**	**Error**
13	Al	Aluminium	39.5	0.1
8	O	Oxygen	39.7	0.4
20	Ca	Calcium	7.5	0.2
26	Fe	Iron	13.2	0.4
16	S	Sulfur	0.0	1.1

4. spot

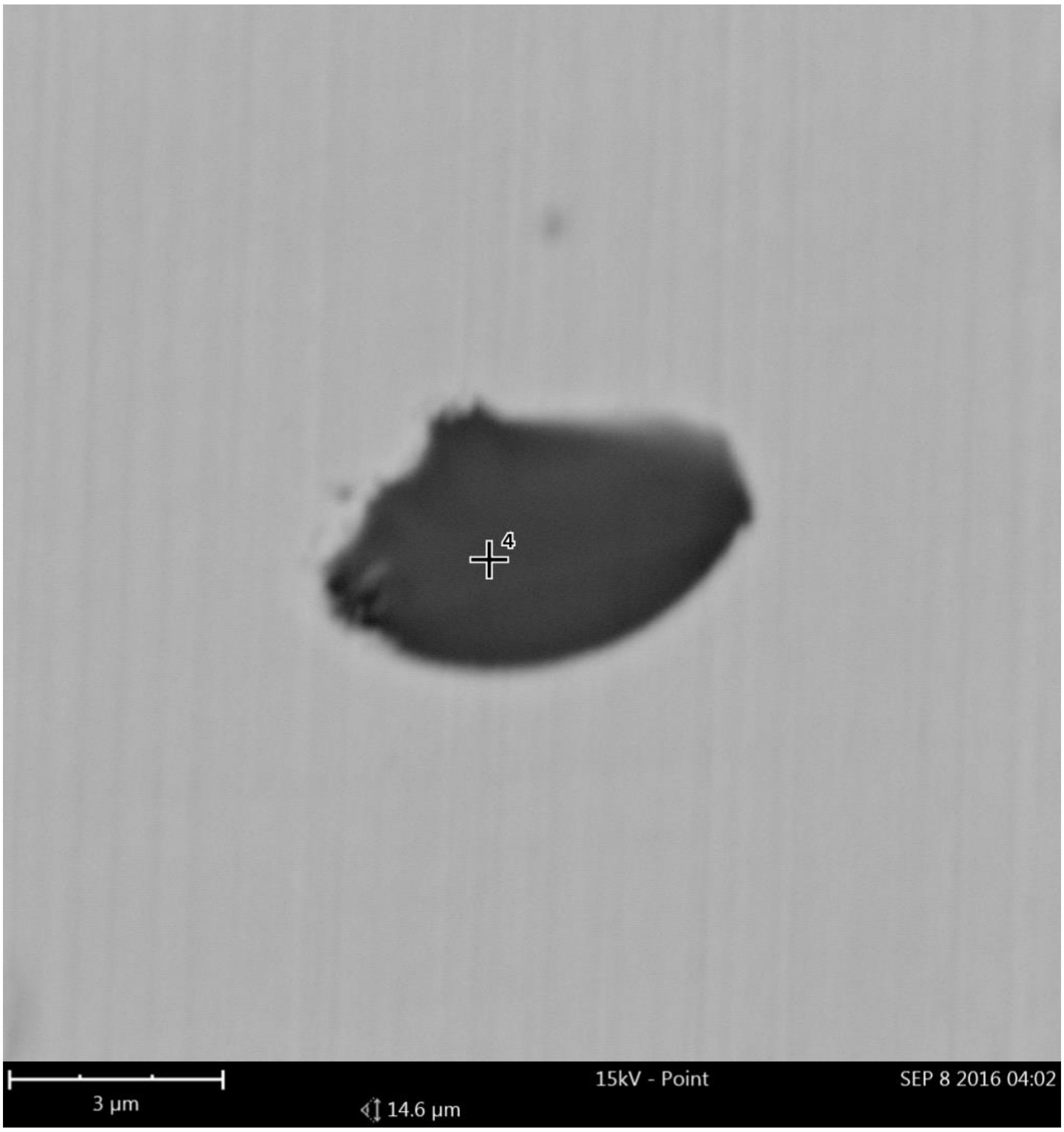

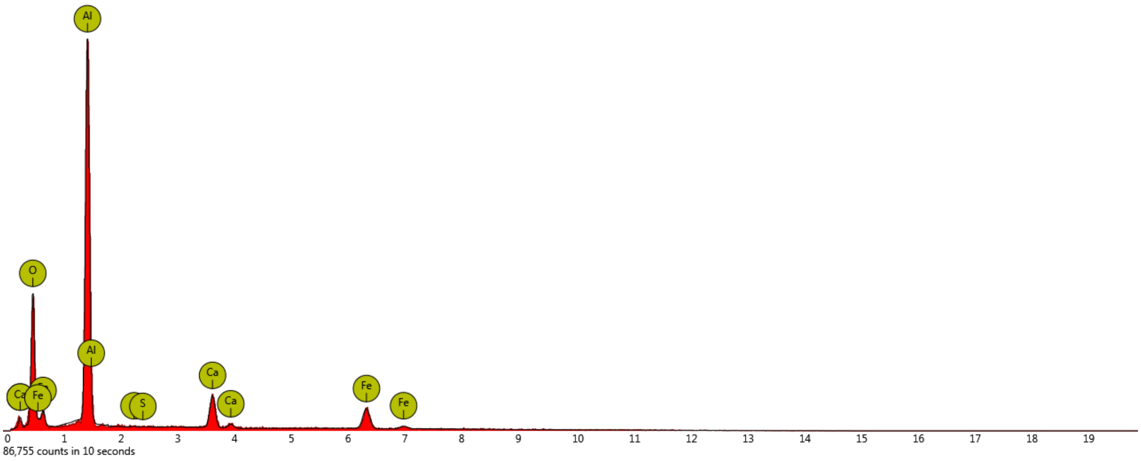

Disabled elements: As, B, Ba, Br, C, Cs, Dy, Er, F, Ga, Hg, La, Lu, Pb, Pm, Pt, Rb, Sb, Si, Sr, Te, Tm, Yb, Zr

**Element Number**	**Element Symbol**	**Element Name**	**Weight Concentration**	**Error**
13	Al	Aluminium	42.2	0.1
8	O	Oxygen	37.7	0.4
20	Ca	Calcium	6.3	0.3

26	Fe	Iron	13.7	0.3
16	S	Sulfur	0.1	1.0

SEM-EDS results of sample A1C1-1800s

Image 1

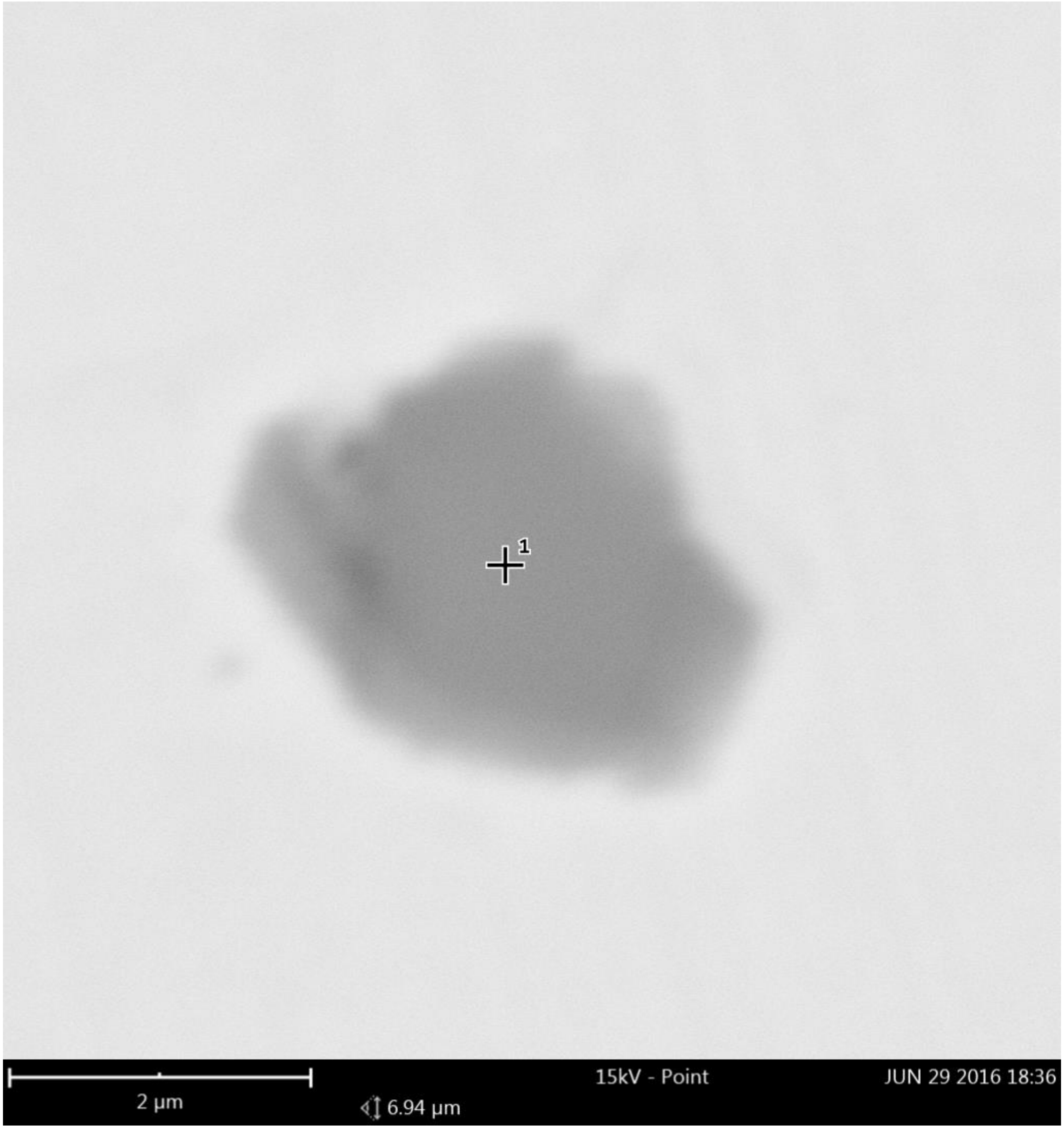

1. spot

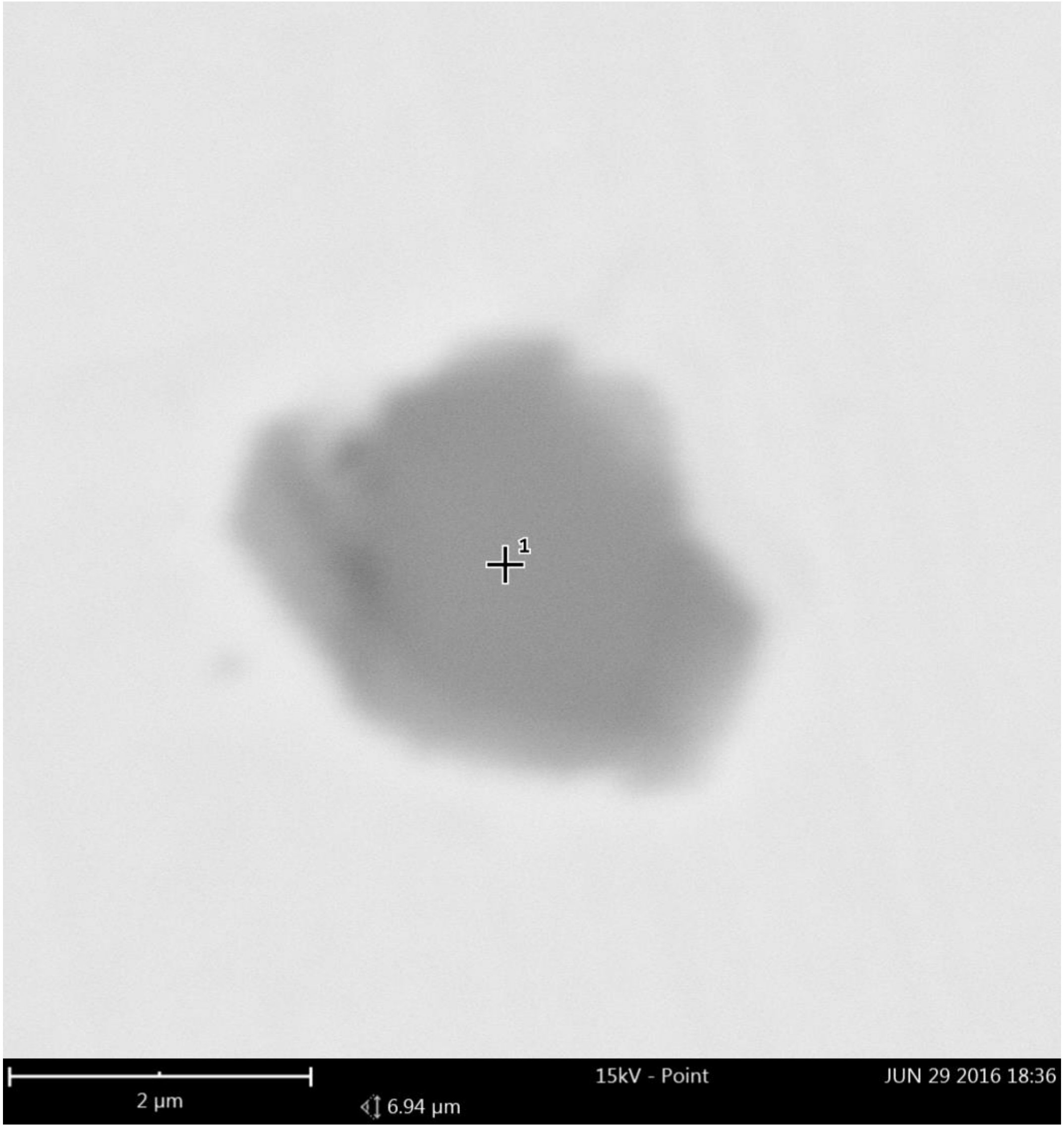

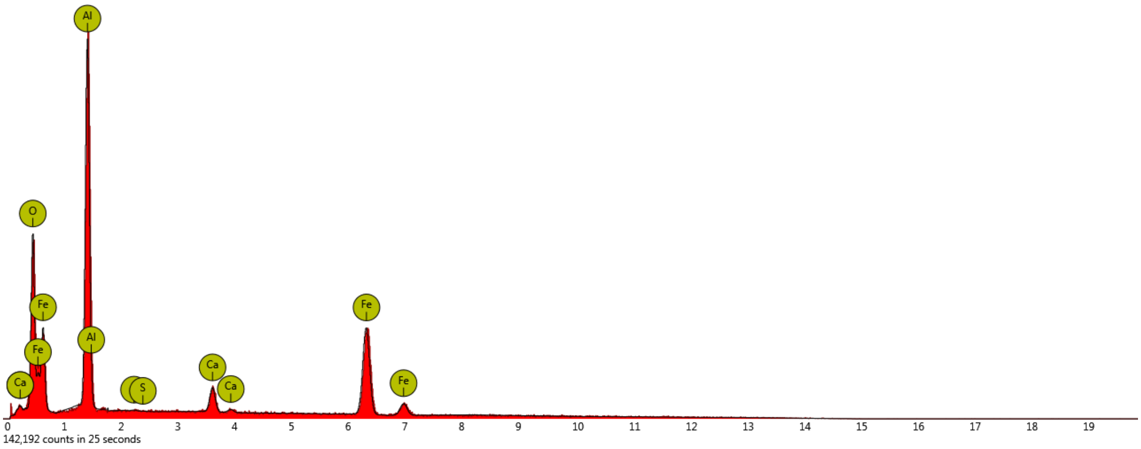

Disabled elements: As, B, Ba, Br, Cs, Dy, Er, F, Ga, Hg, Lu, Pt, Sb, Te, Tm, Yb, Zr

Element Number	Element Symbol	Element Name	Weight Concentration	Error
13	Al	Aluminium	32.0	0.3
8	O	Oxygen	26.7	0.7
26	Fe	Iron	38.2	0.5
20	Ca	Calcium	3.0	0.5

16	S	Sulfur	0.1	1.3

Image 2

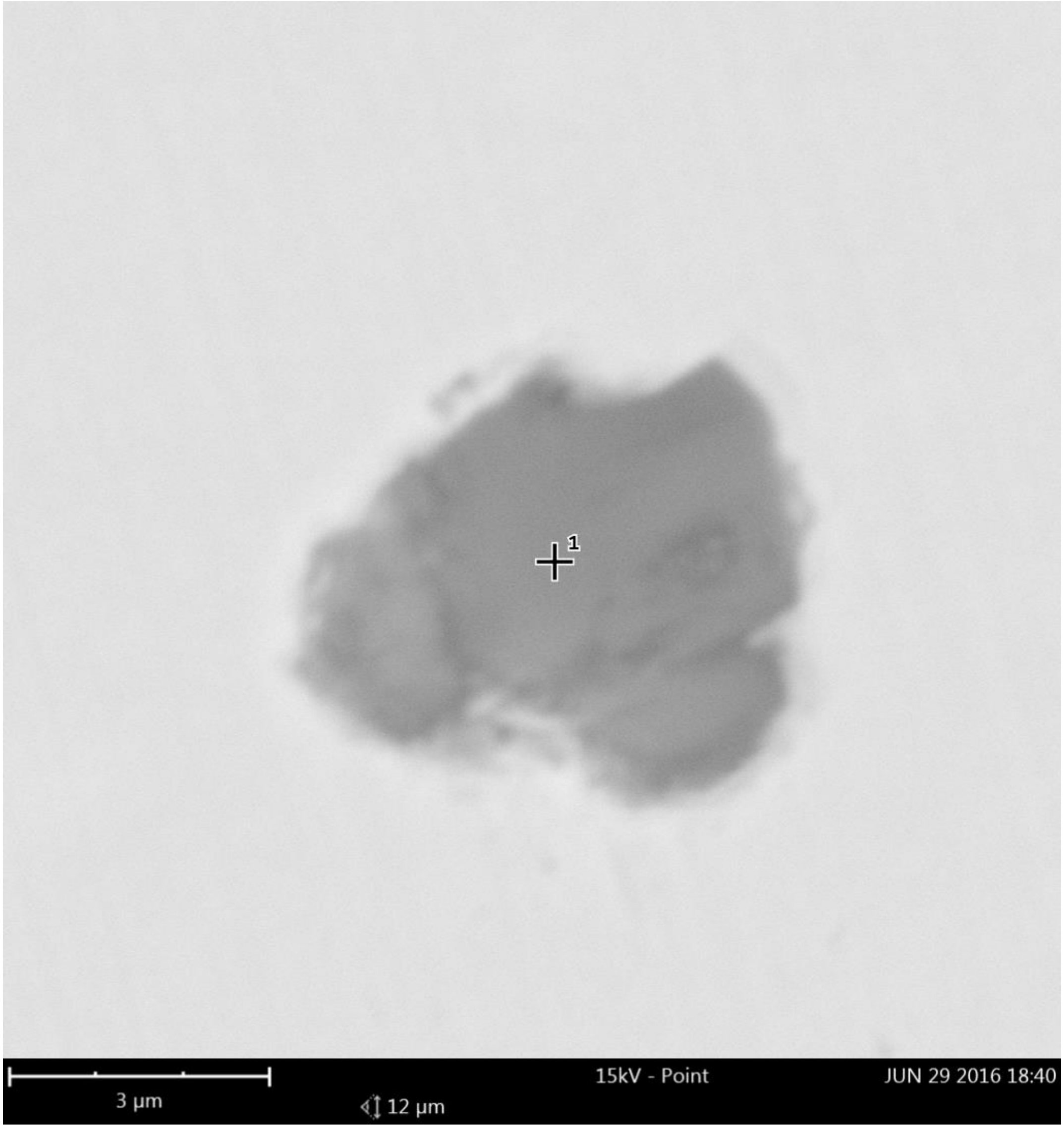

1. spot

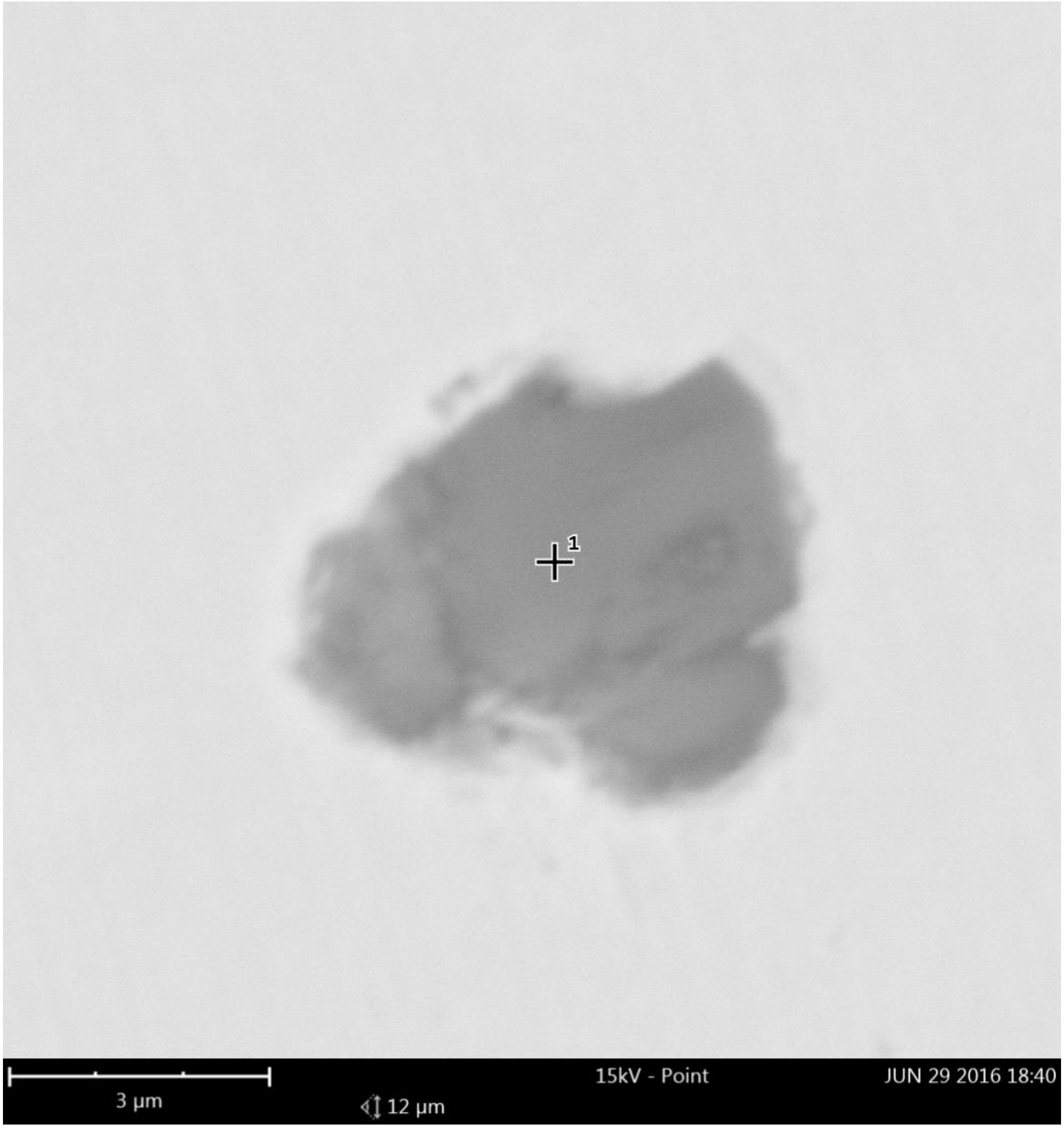

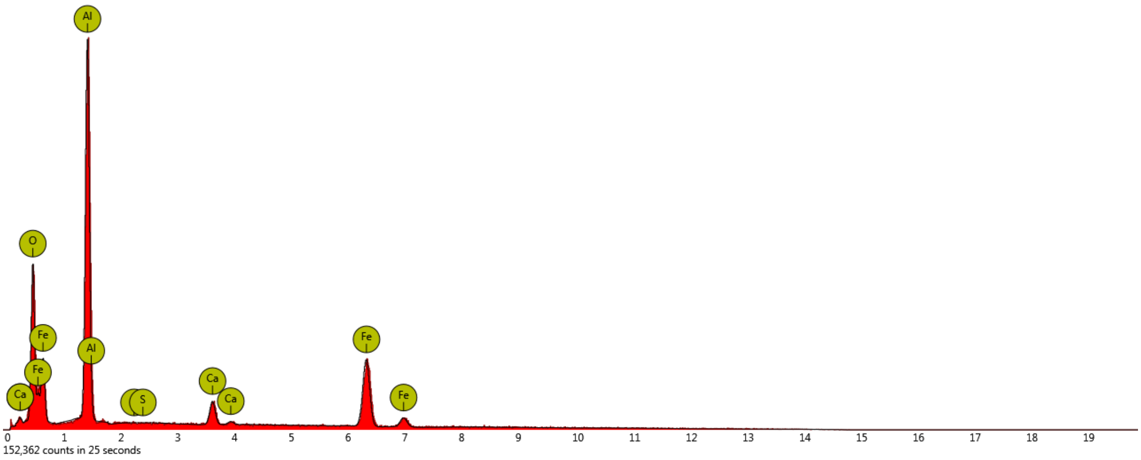

Disabled elements: As, B, Ba, Br, Cs, Dy, Er, F, Ga, Hg, Lu, Pt, Sb, Te, Tm, Yb, Zr

Element Number	Element Symbol	Element Name	Weight Concentration	Error
13	Al	Aluminium	35.6	0.2
8	O	Oxygen	27.9	0.7
26	Fe	Iron	33.2	0.5
20	Ca	Calcium	3.3	0.6

16	S	Sulfur	0.0	1.3

Image 3

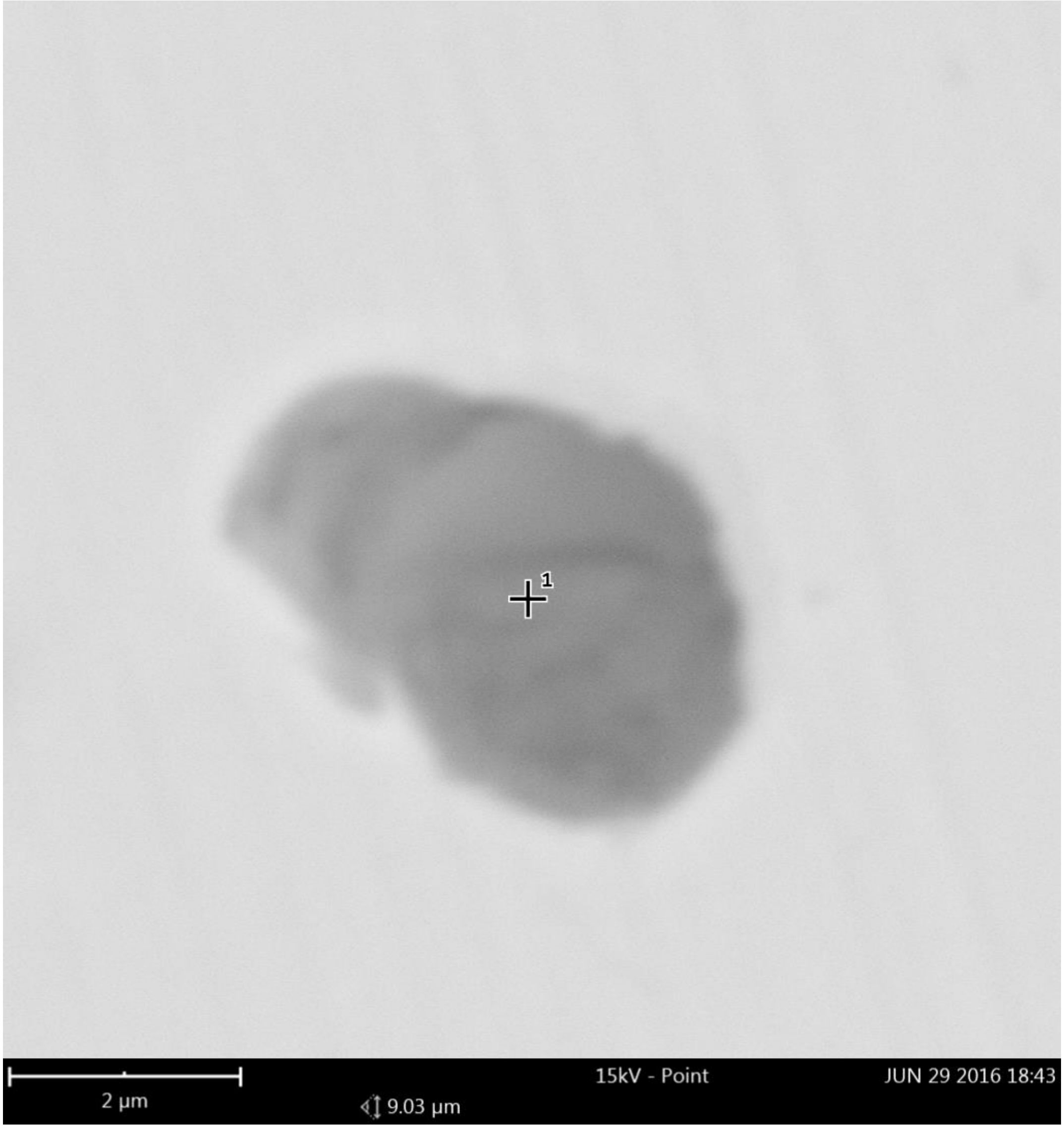

1. spot

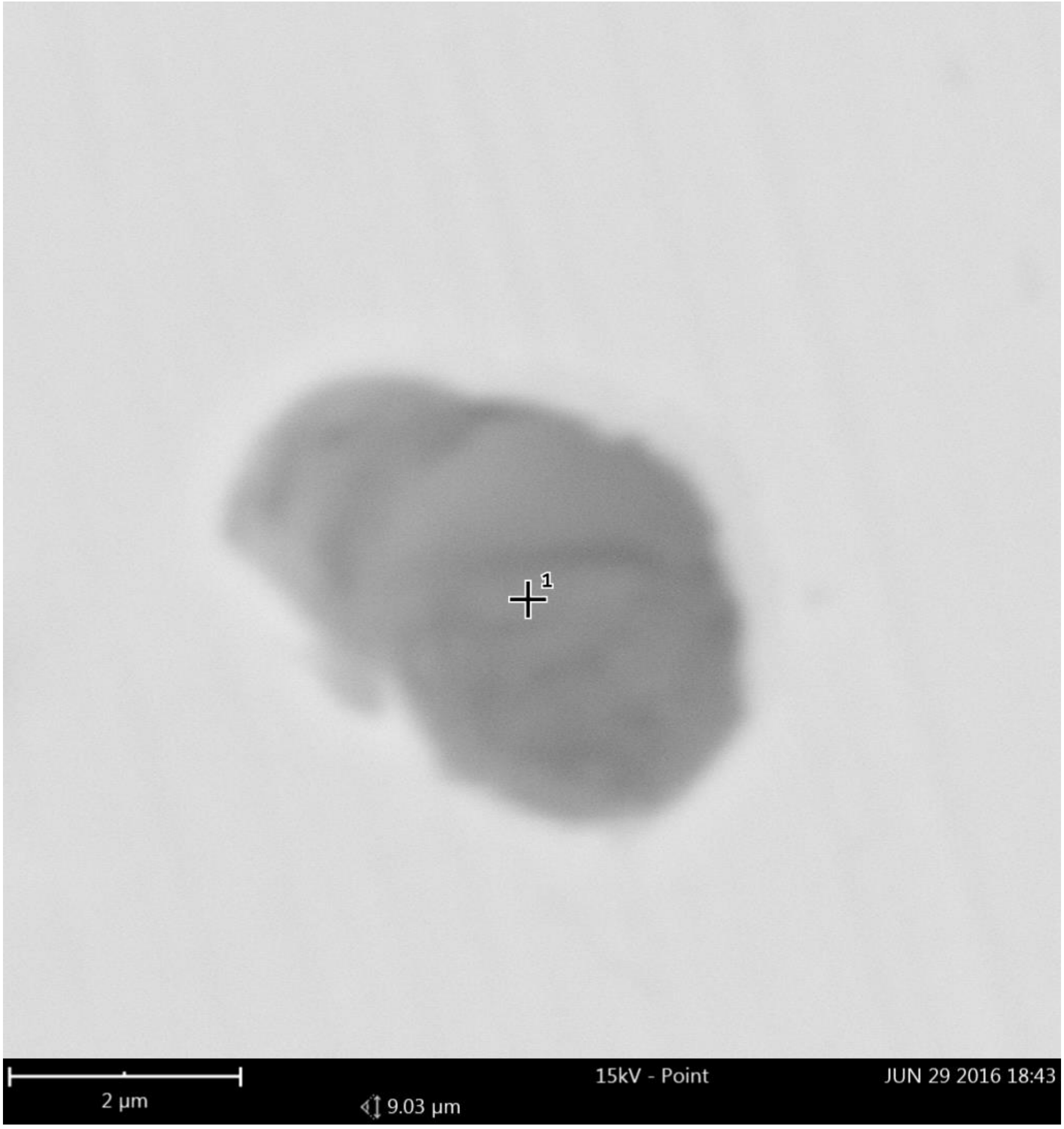

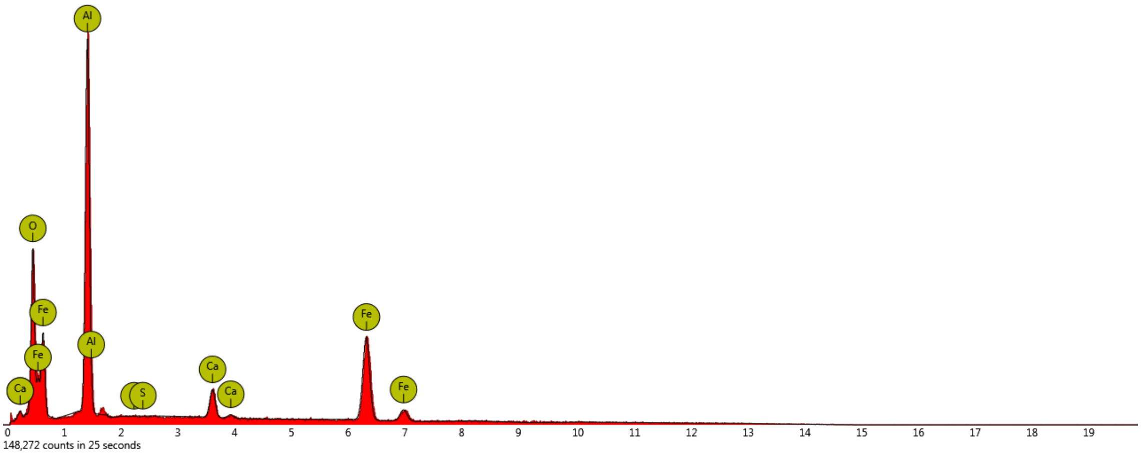

Disabled elements: As, B, Ba, Br, Cs, Dy, Er, F, Ga, Hg, Lu, Pt, Sb, Te, Tm, Yb, Zr

Element Number	Element Symbol	Element Name	Weight Concentration	Error
13	Al	Aluminium	32.8	0.2
8	O	Oxygen	26.5	0.7
26	Fe	Iron	37.2	0.4
20	Ca	Calcium	3.5	0.4

16	S	Sulfur	0.0	1.2

Image 4

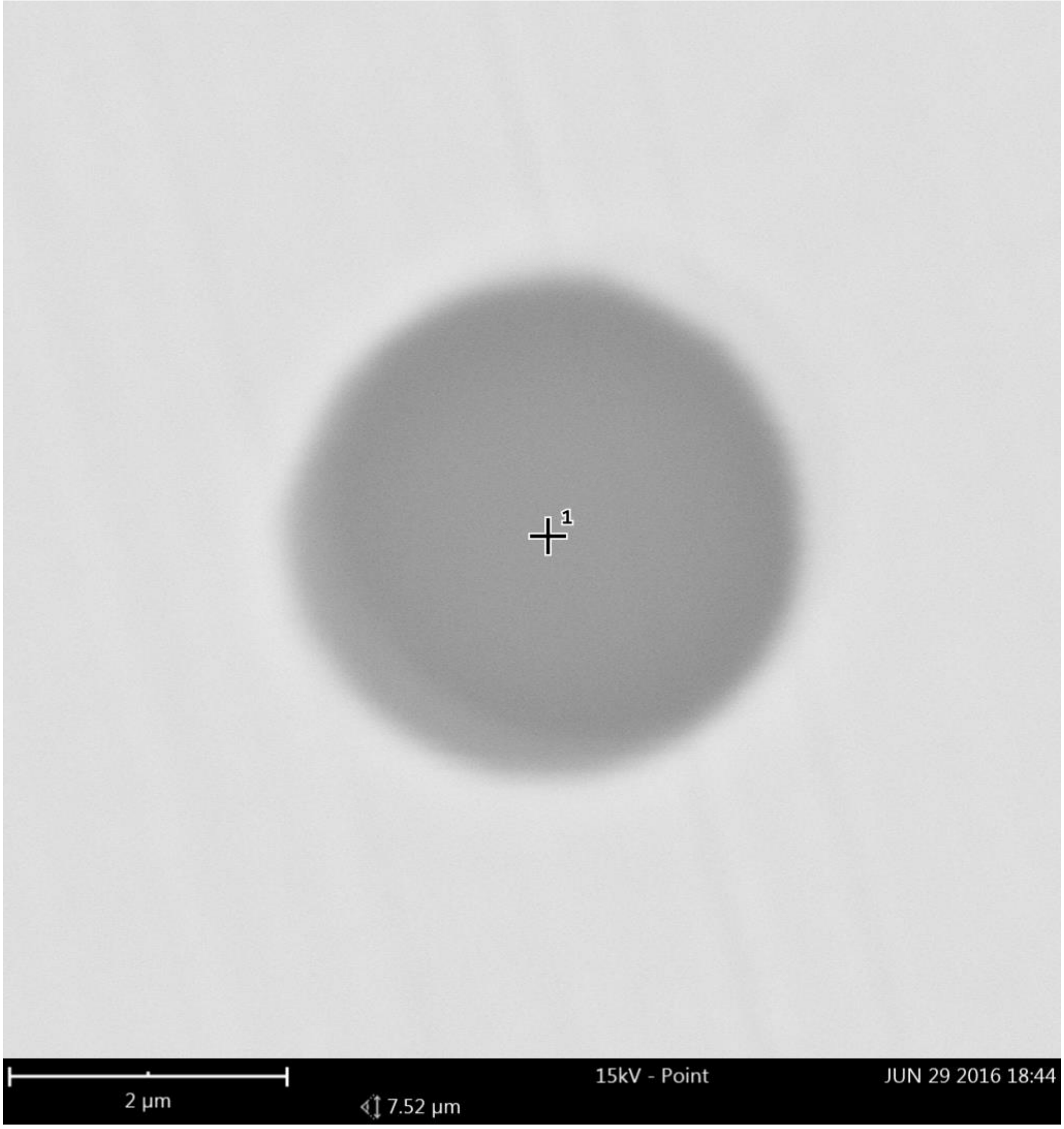

1. spot

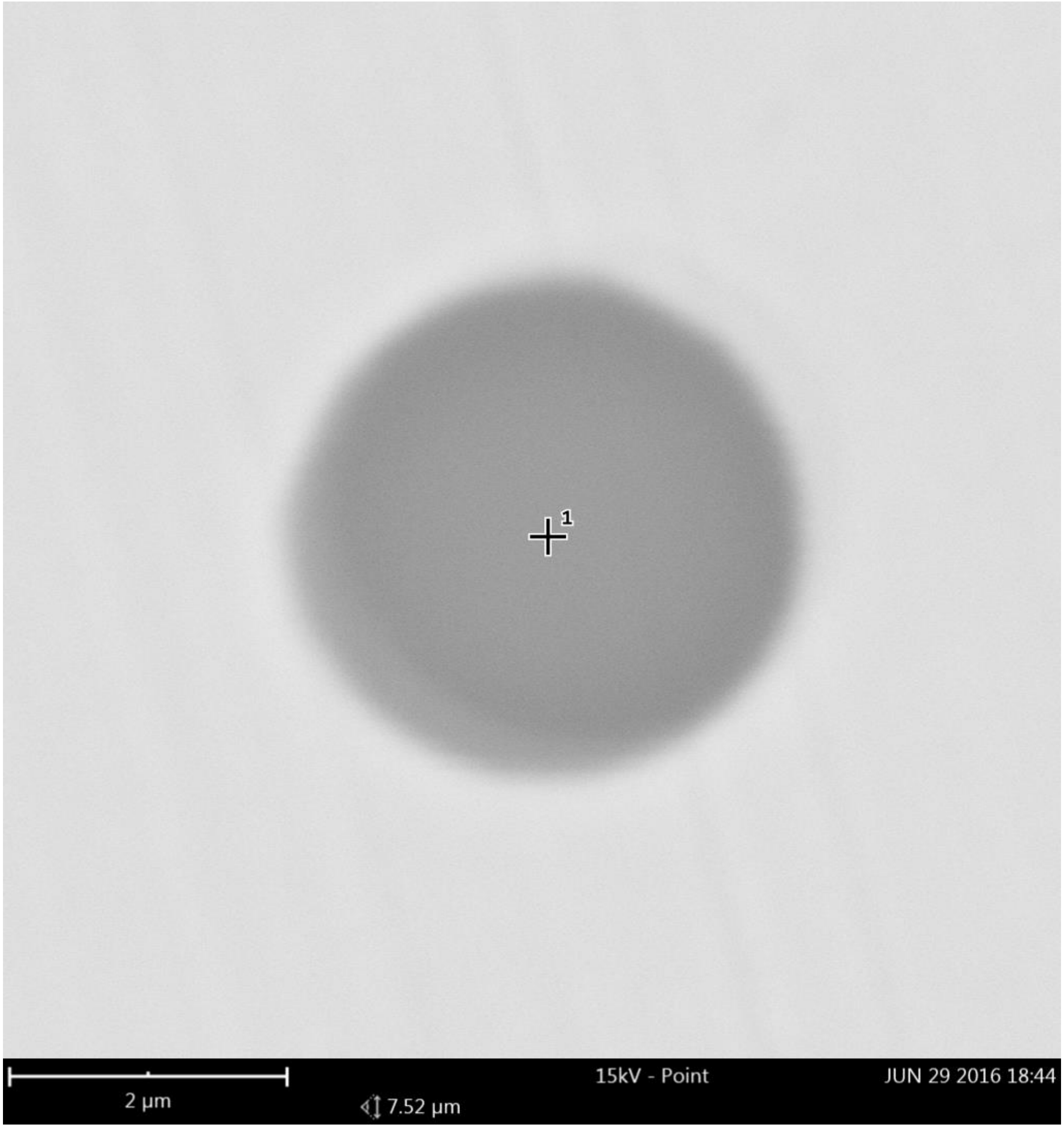

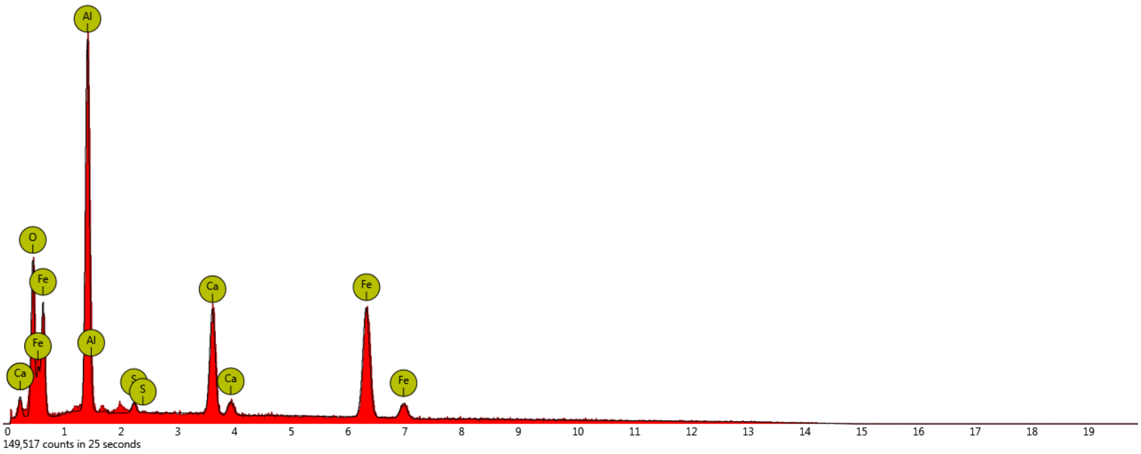

Disabled elements: As, B, Ba, Br, Cs, Dy, Er, F, Ga, Hg, Lu, Pt, Sb, Te, Tm, Yb, Zr

Element Number	Element Symbol	Element Name	Weight Concentration	Error
13	Al	Aluminium	26.3	0.2
26	Fe	Iron	38.4	0.4
20	Ca	Calcium	10.3	0.5
8	O	Oxygen	24.2	1.0

16	S	Sulfur	0.8	0.7

Image 5

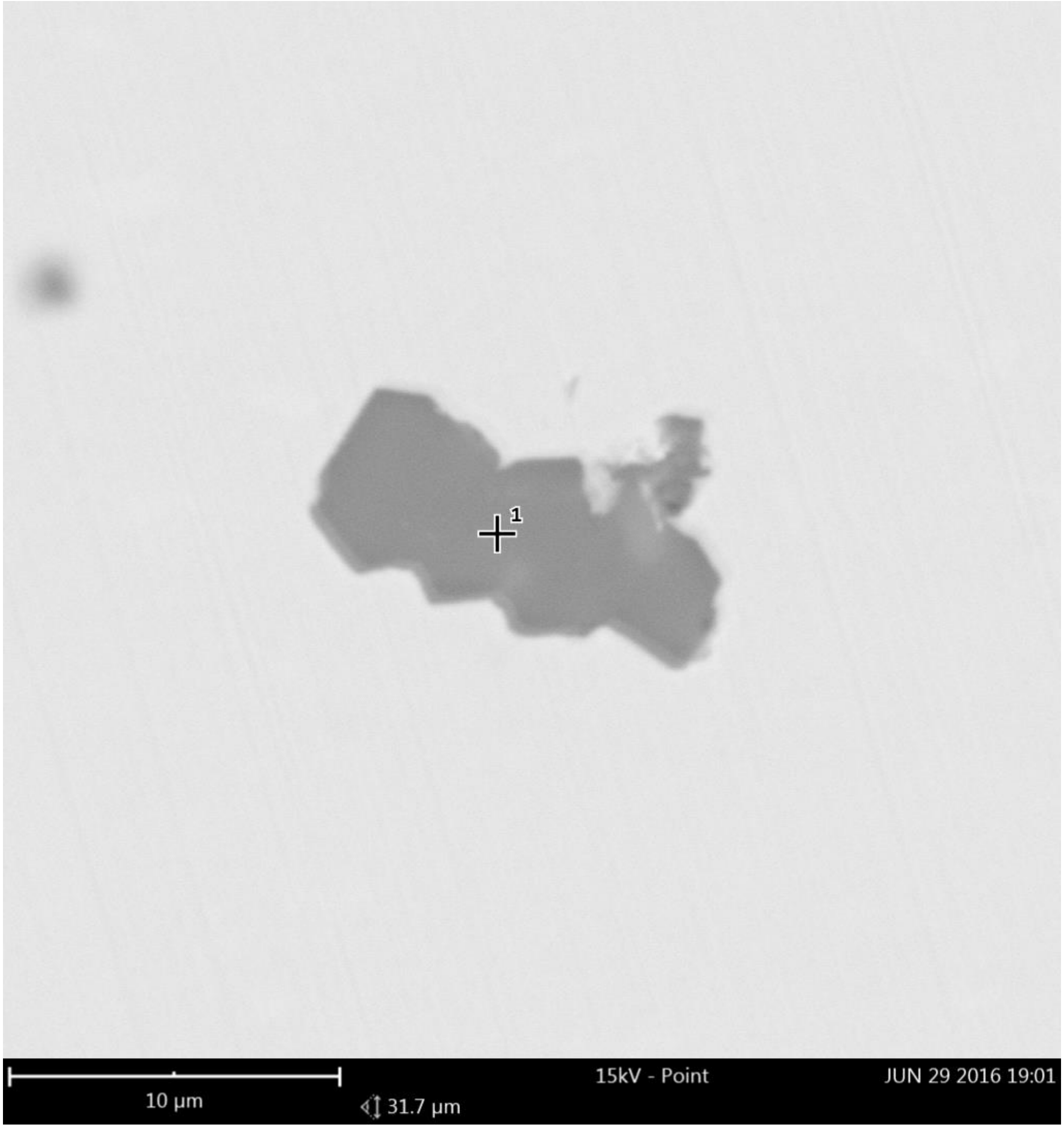

1. spot

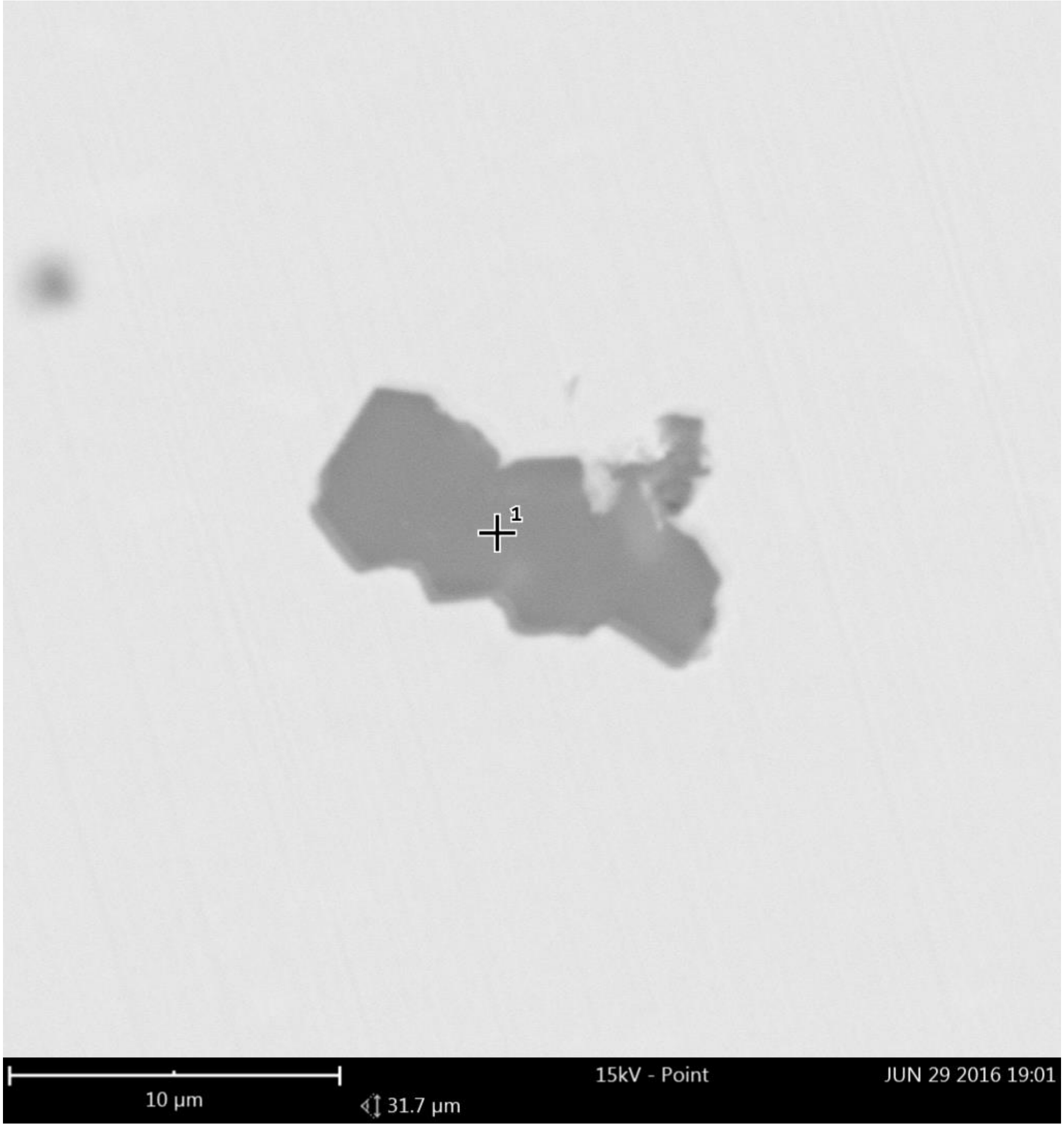

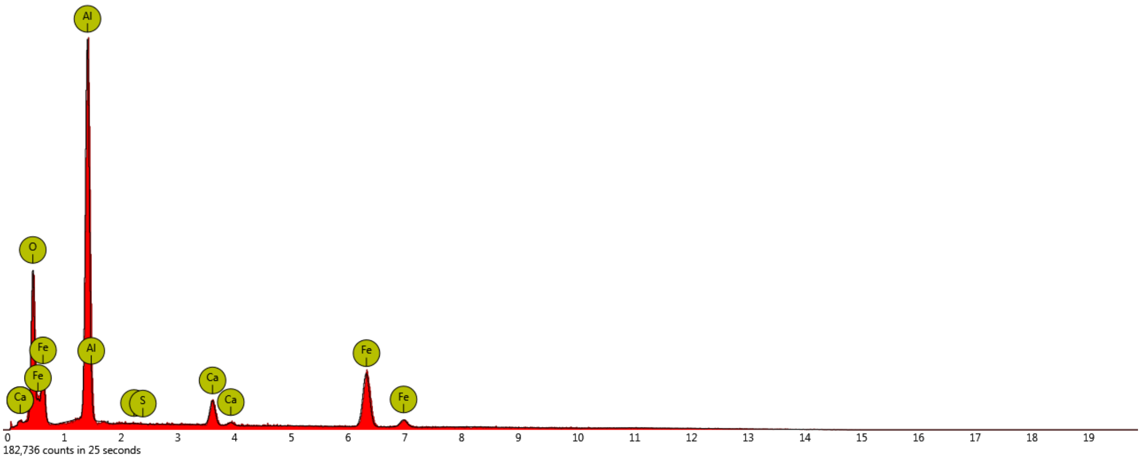

Disabled elements: As, B, Ba, Br, Cs, Dy, Er, F, Ga, Hg, Lu, Pt, Sb, Te, Tm, Yb, Zr

Element Number	Element Symbol	Element Name	Weight Concentration	Error
13	Al	Aluminium	37.3	0.3
8	O	Oxygen	30.0	0.8
26	Fe	Iron	28.9	0.4
20	Ca	Calcium	3.7	0.4

16	S	Sulfur	0.1	1.0

SEM-EDS results of sample A1C1-3900s

Image 1

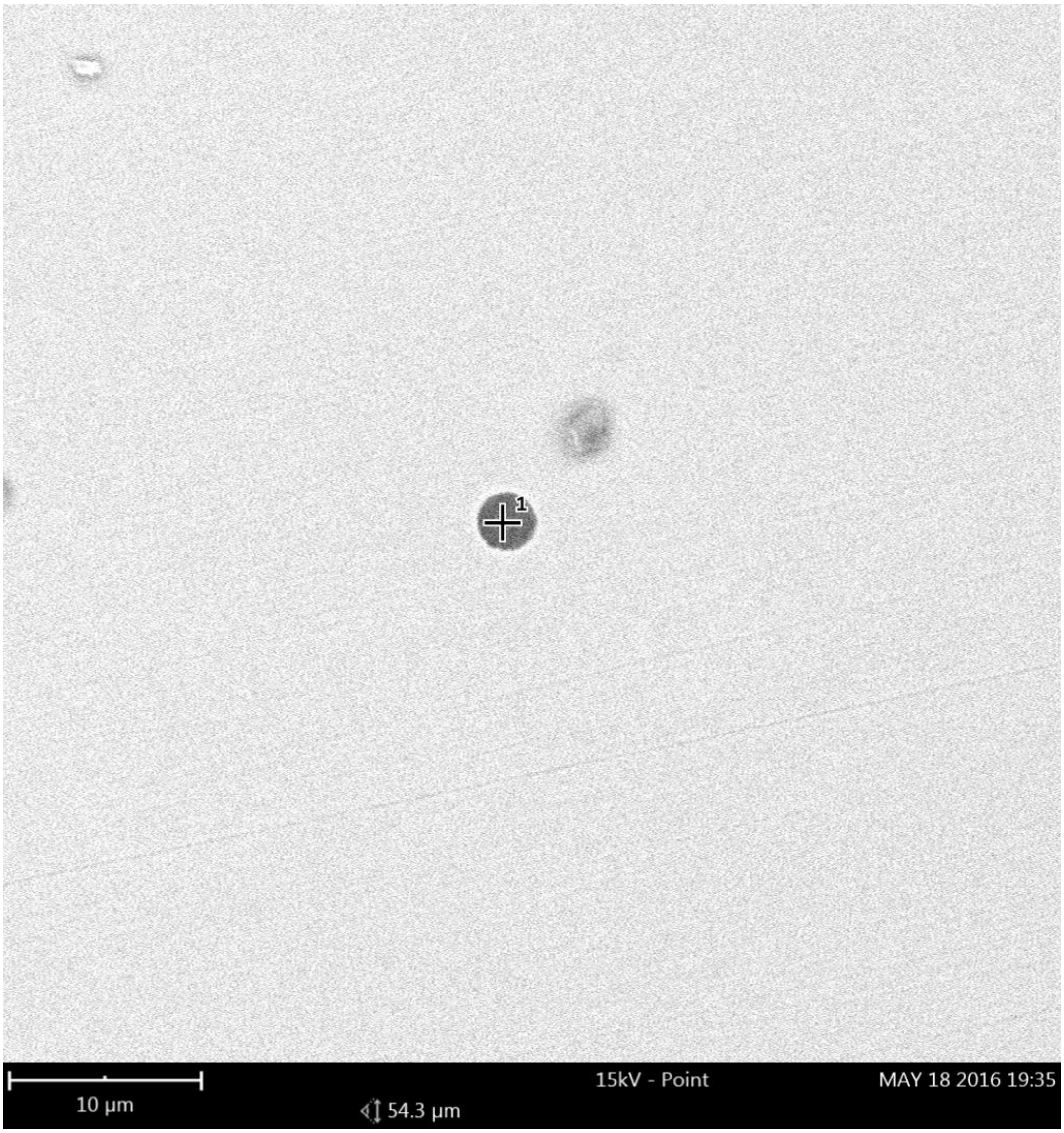

1. spot

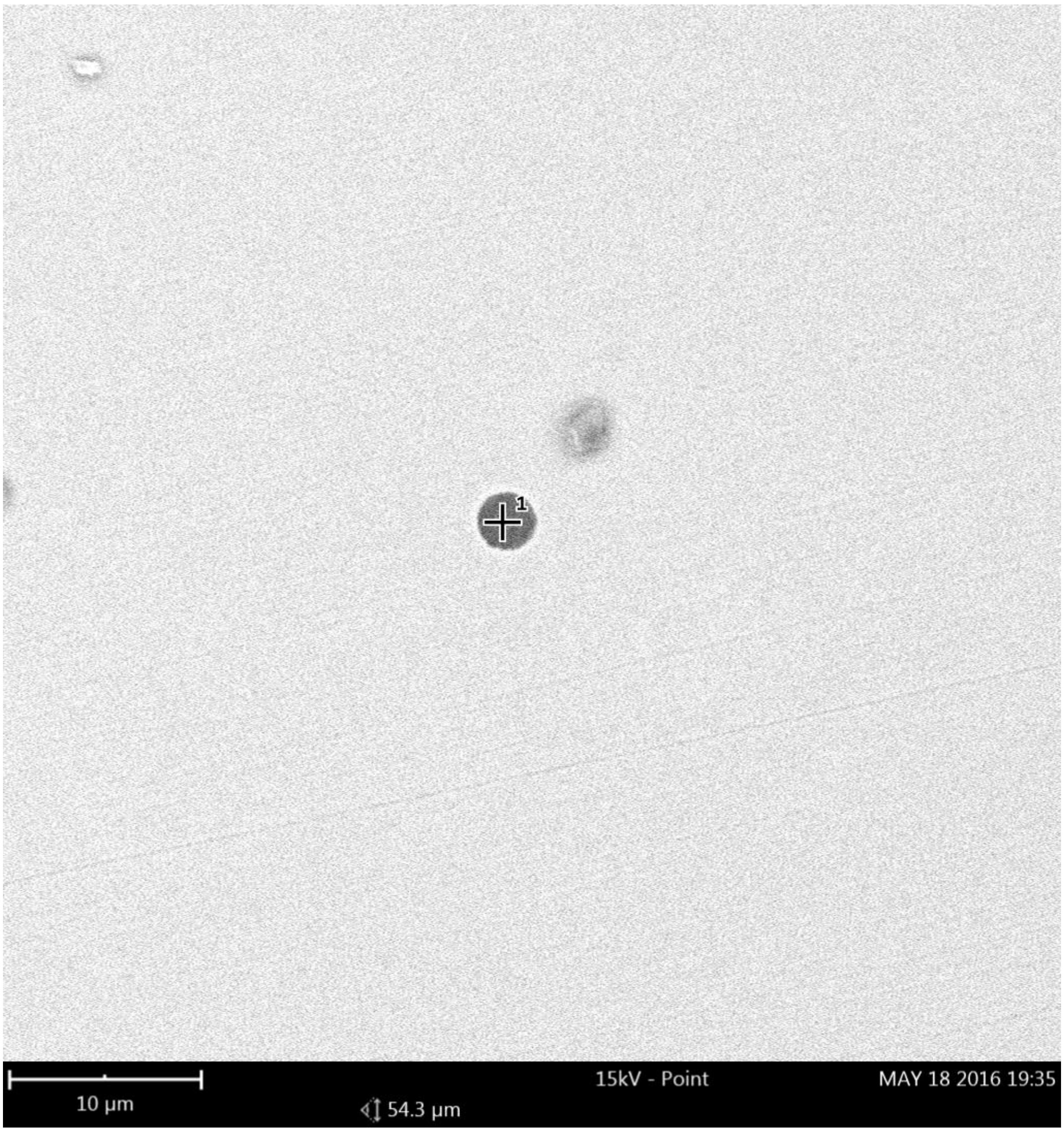

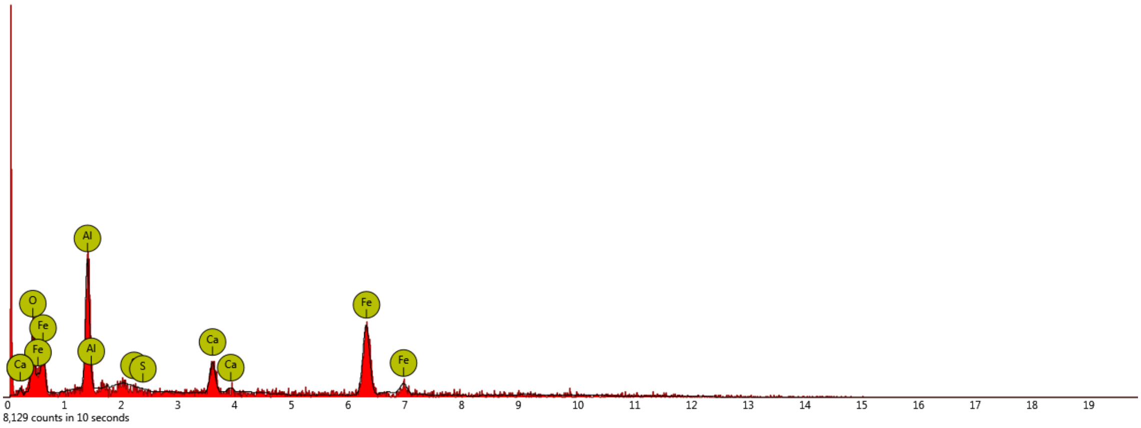

Disabled elements: As, B, Ba, Br, Cs, Dy, Er, F, Ga, Hg, Lu, Pt, Sb, Te, Tm, Yb, Zr

Element Number	Element Symbol	Element Name	Weight Concentration	Error
13	Al	Aluminium	22.1	0.3
26	Fe	Iron	52.2	0.4
8	O	Oxygen	19.5	0.7
20	Ca	Calcium	6.2	0.9

16	S	Sulfur	0.0	2.2

Image 2

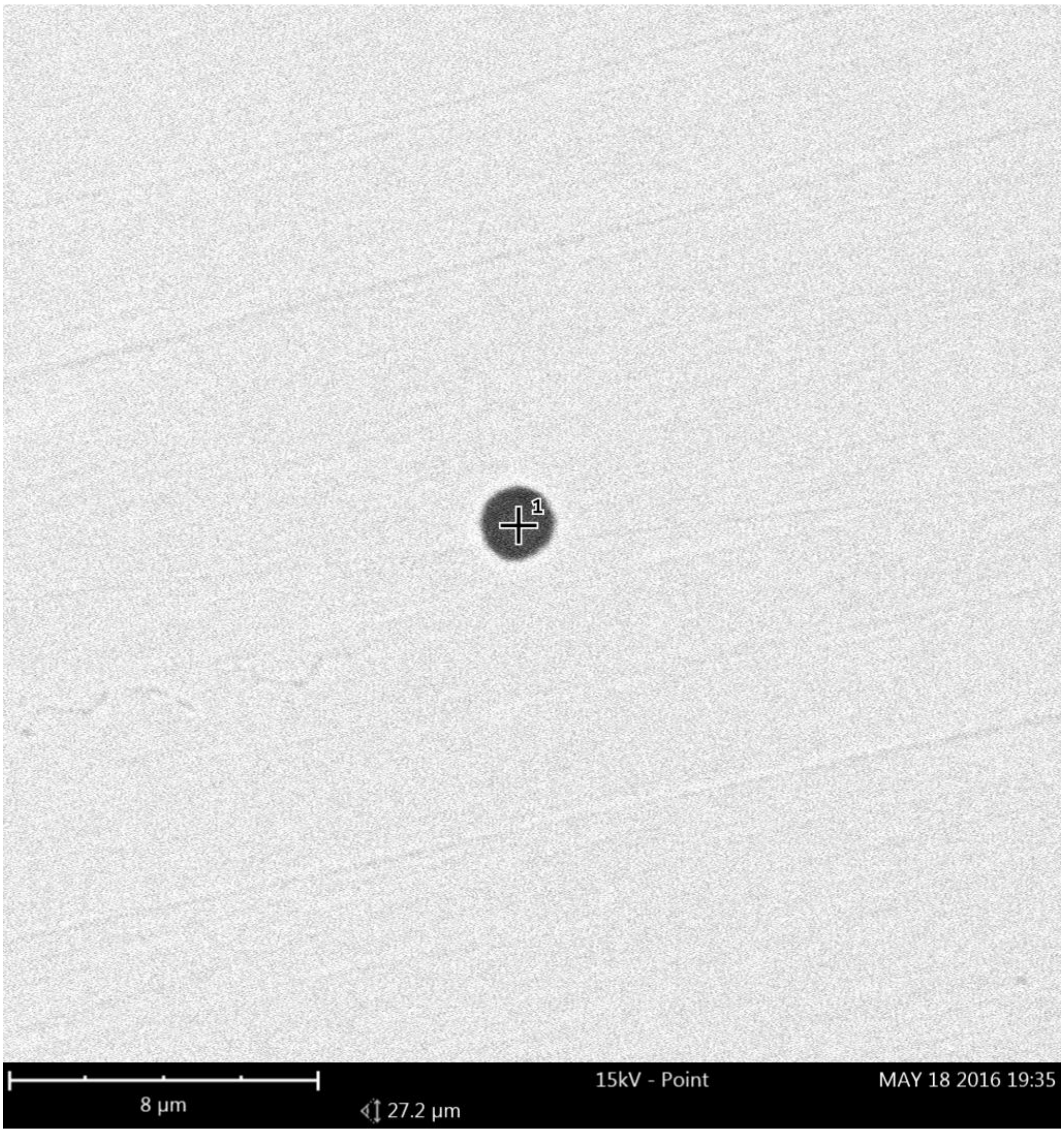

1. spot

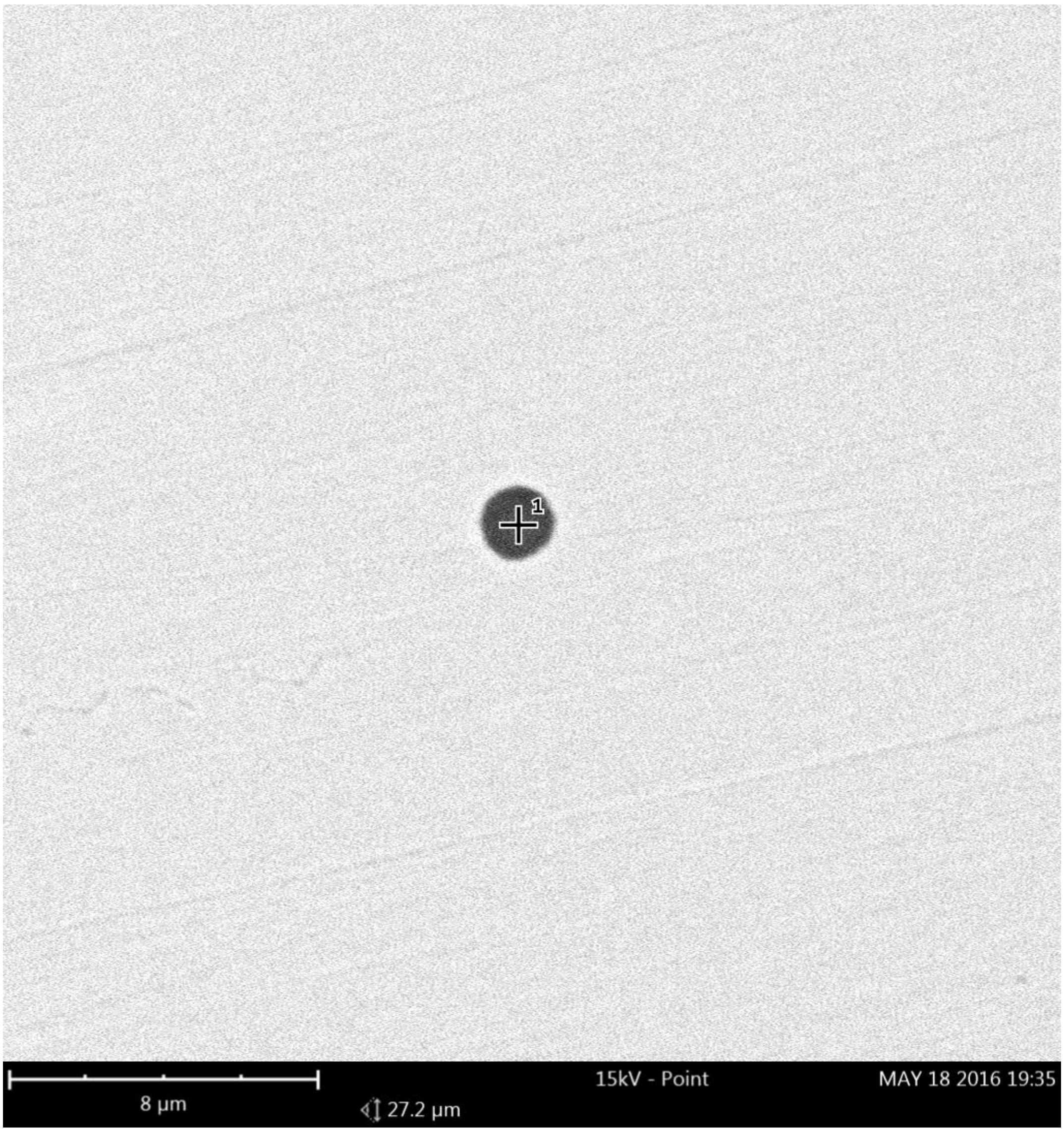

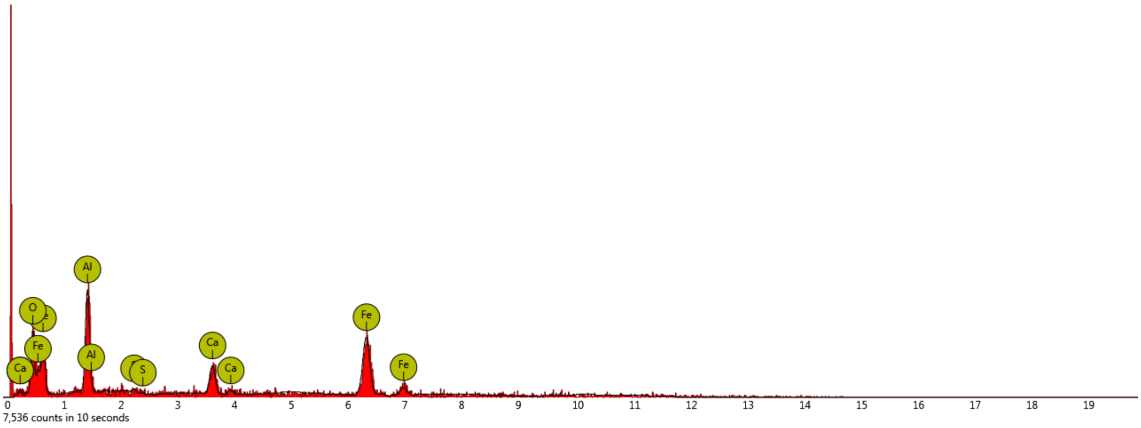

Disabled elements: As, B, Ba, Br, Cs, Dy, Er, F, Ga, Hg, Lu, Pt, Sb, Te, Tm, Yb, Zr

Element Number	Element Symbol	Element Name	Weight Concentration	Error
13	Al	Aluminium	20.4	0.4
26	Fe	Iron	50.6	0.6
8	O	Oxygen	21.5	0.5
20	Ca	Calcium	7.1	1.0

16	S	Sulfur	0.5	1.7

Image 3

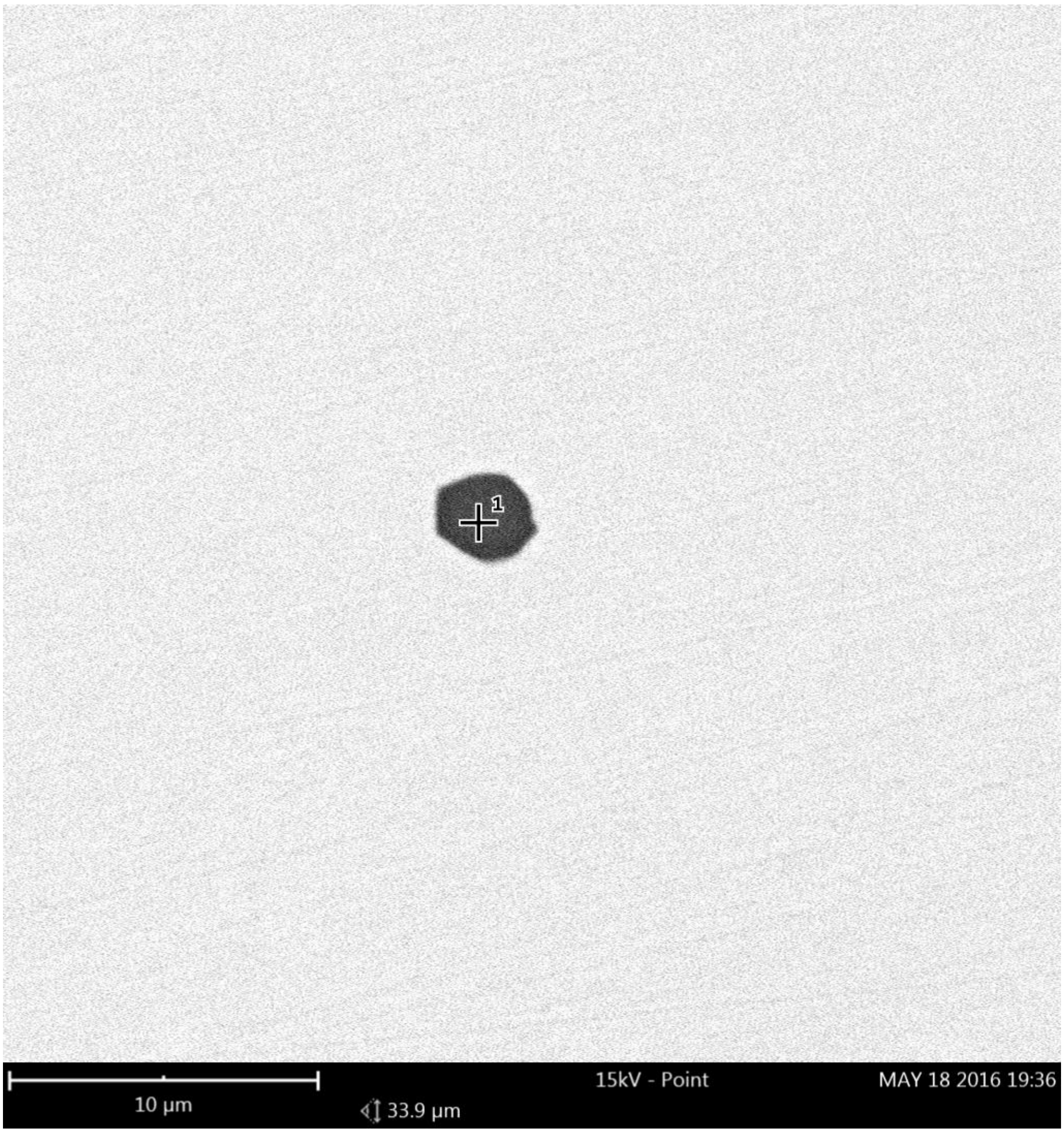

1. spot

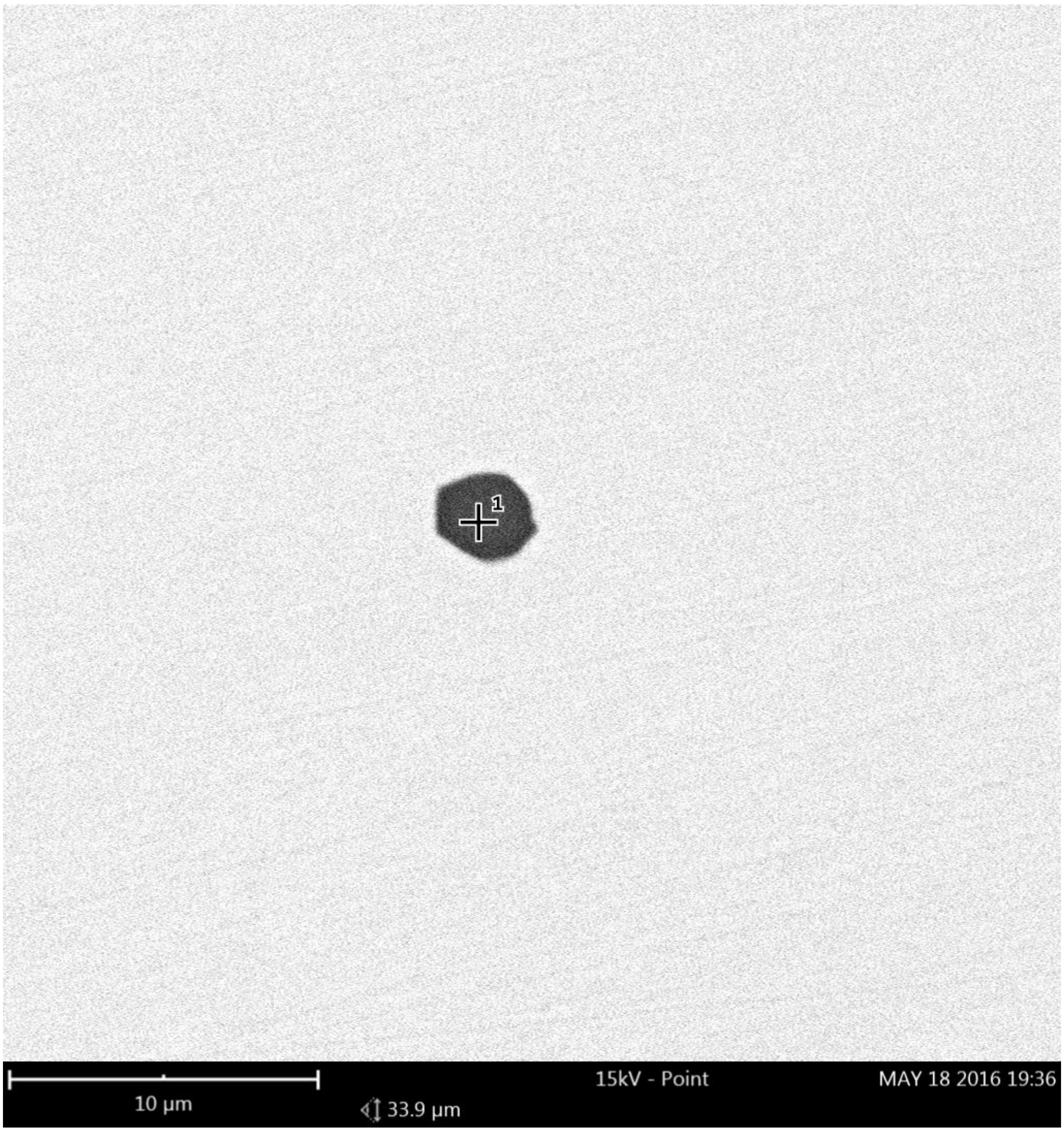

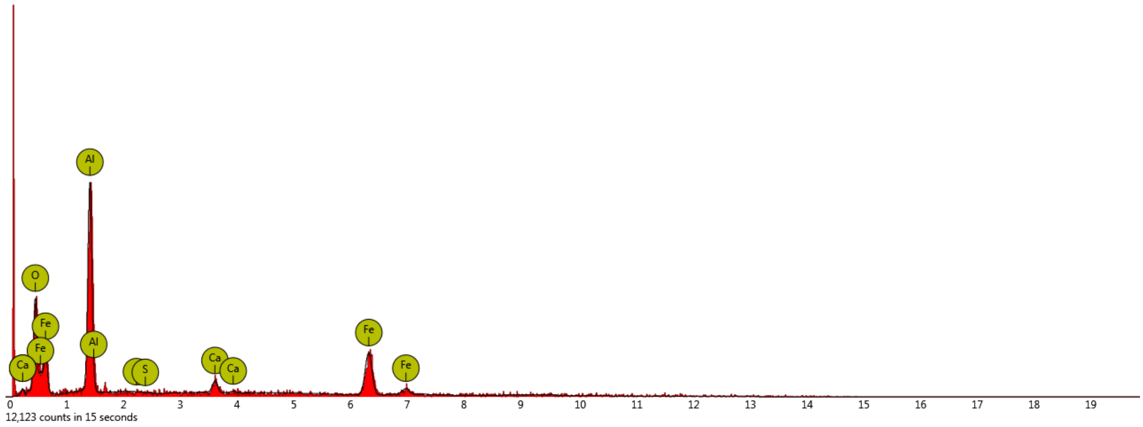

Disabled elements: As, B, Ba, Br, Cs, Dy, Er, F, Ga, Hg, Lu, Pt, Sb, Te, Tm, Yb, Zr

Element Number	Element Symbol	Element Name	Weight Concentration	Error
13	Al	Aluminium	34.3	0.4
8	O	Oxygen	27.2	1.0
26	Fe	Iron	35.7	0.7
20	Ca	Calcium	2.8	0.7

16	S	Sulfur	0.0	1.3

Image 4

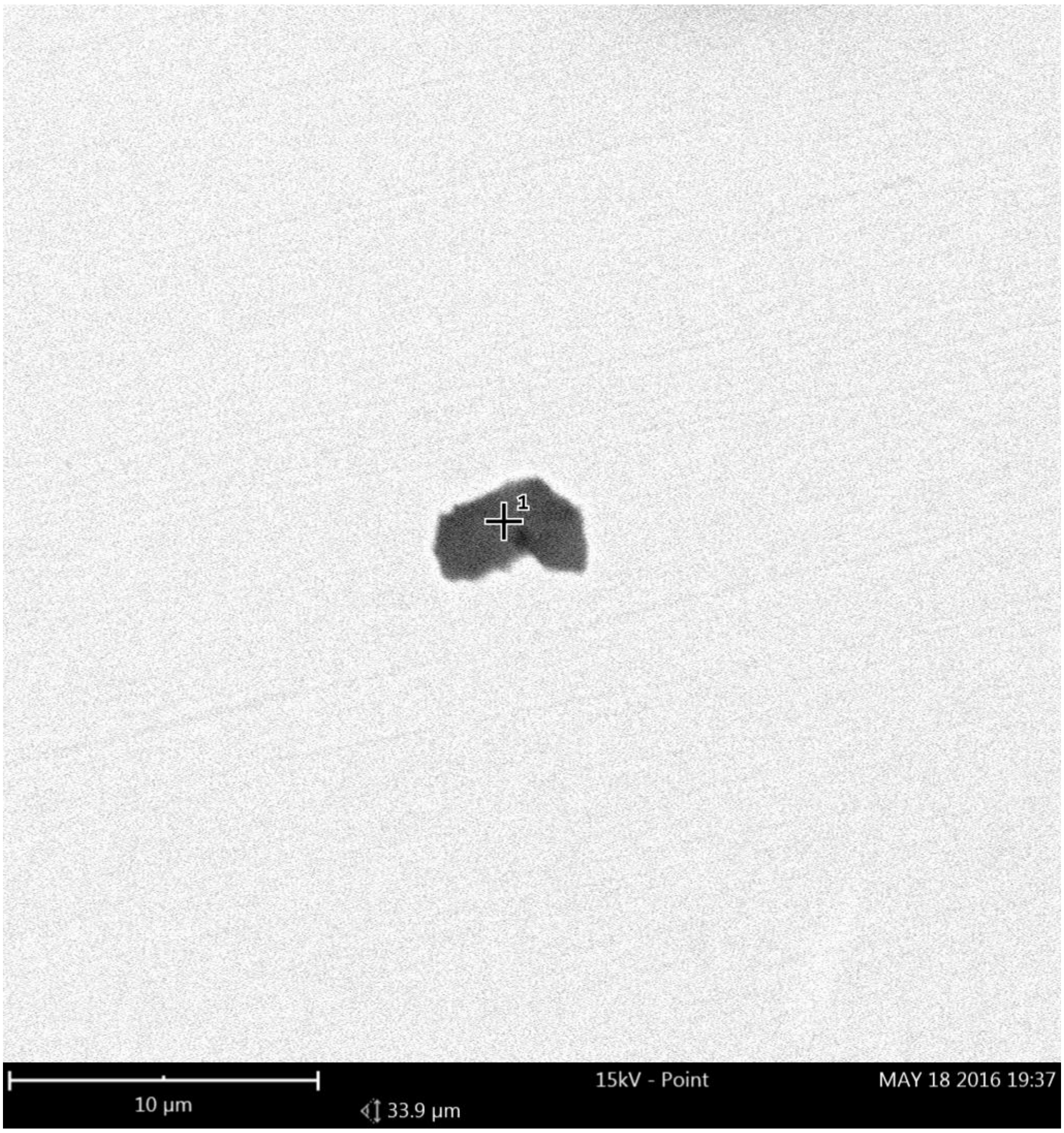

1. spot

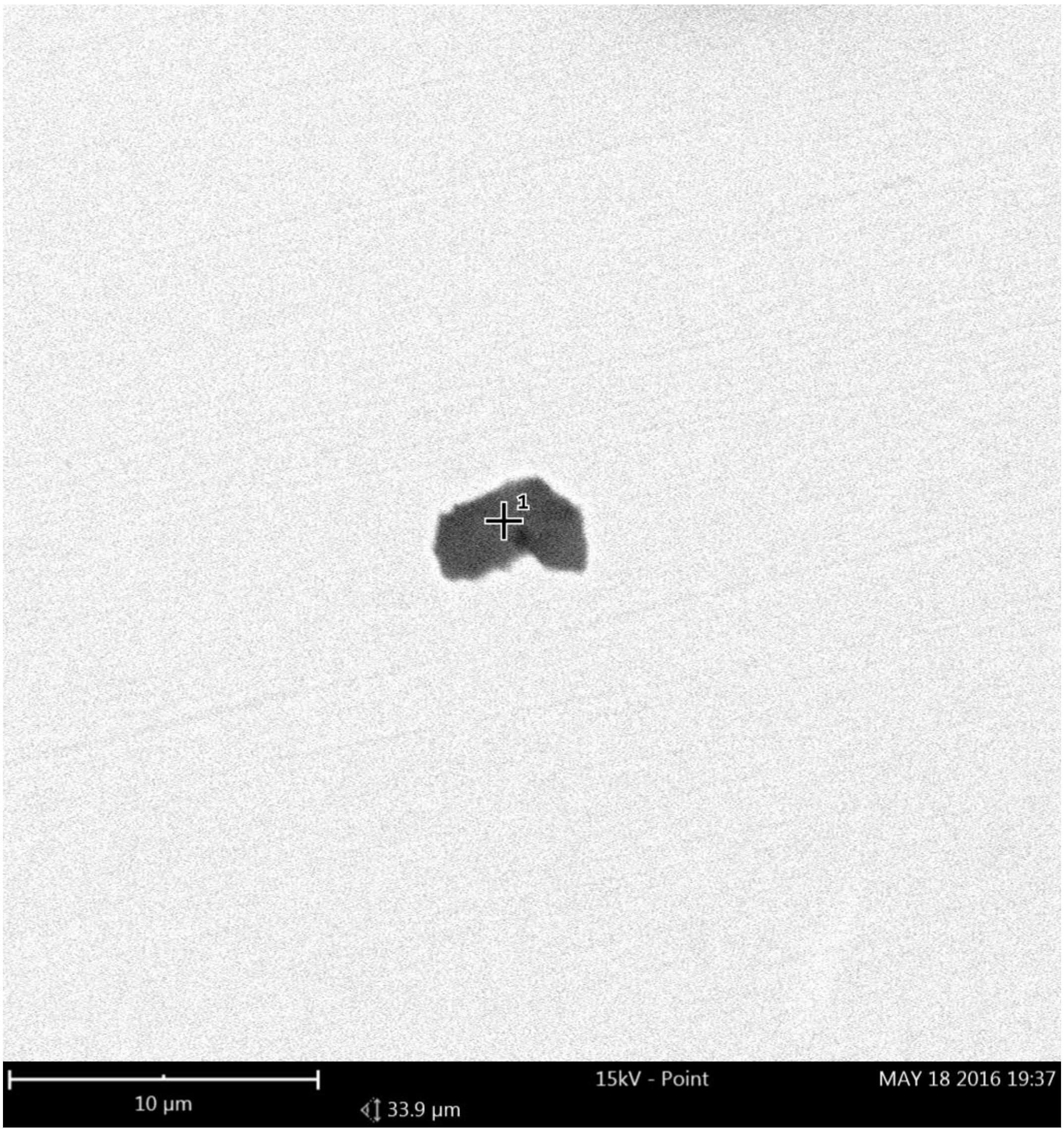

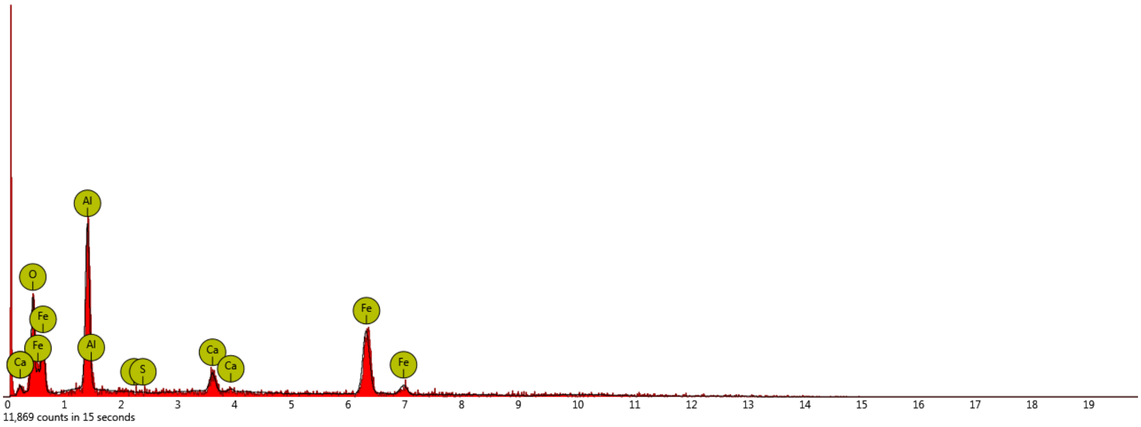

Disabled elements: As, B, Ba, Br, Cs, Dy, Er, F, Ga, Hg, Lu, Pt, Sb, Te, Tm, Yb, Zr

Element Number	Element Symbol	Element Name	Weight Concentration	Error
13	Al	Aluminium	26.2	0.3
26	Fe	Iron	46.1	0.6
8	O	Oxygen	24.0	0.6
20	Ca	Calcium	3.7	1.0

16	S	Sulfur	0.0	1.0

Image 5

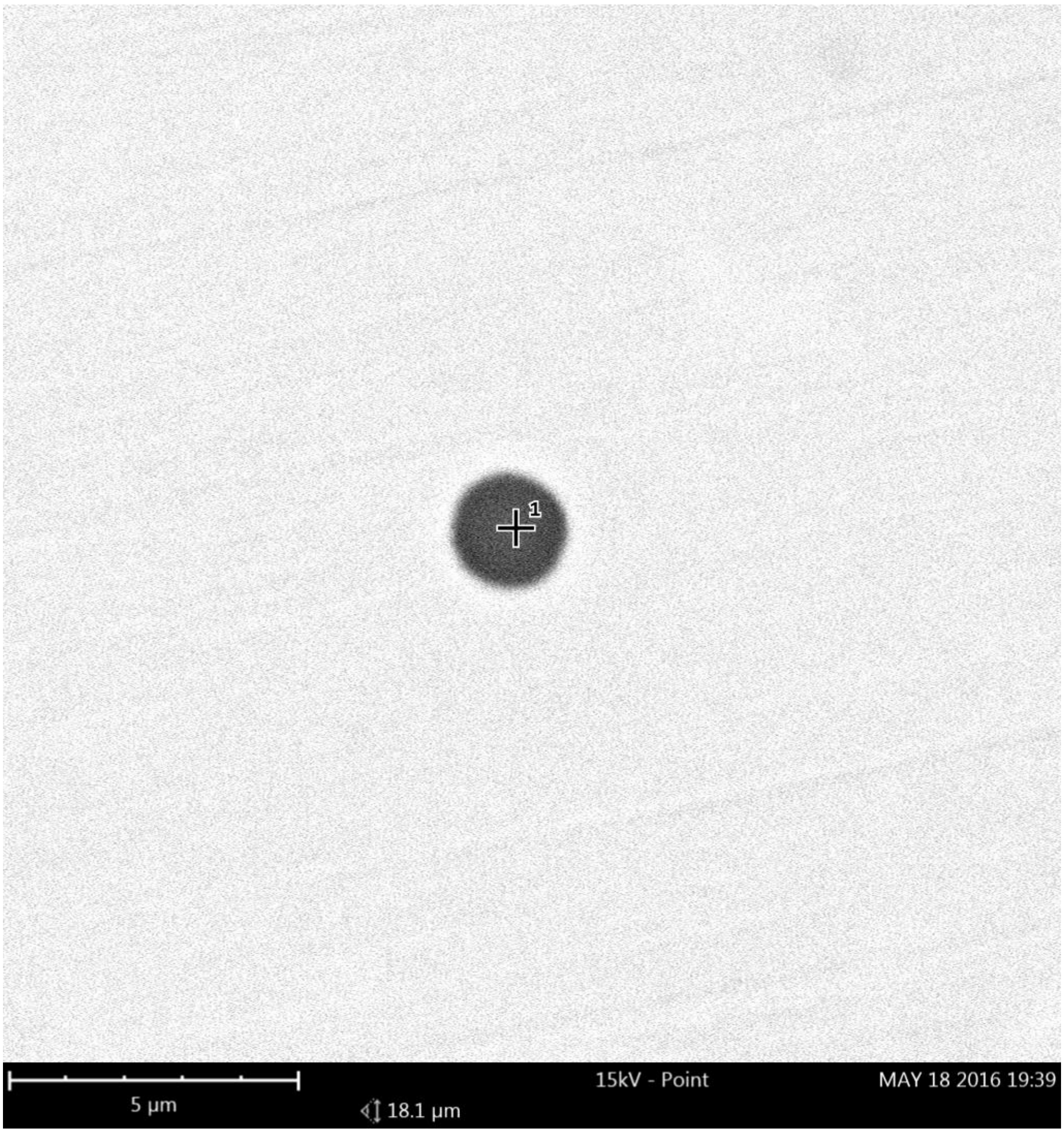

1. spot

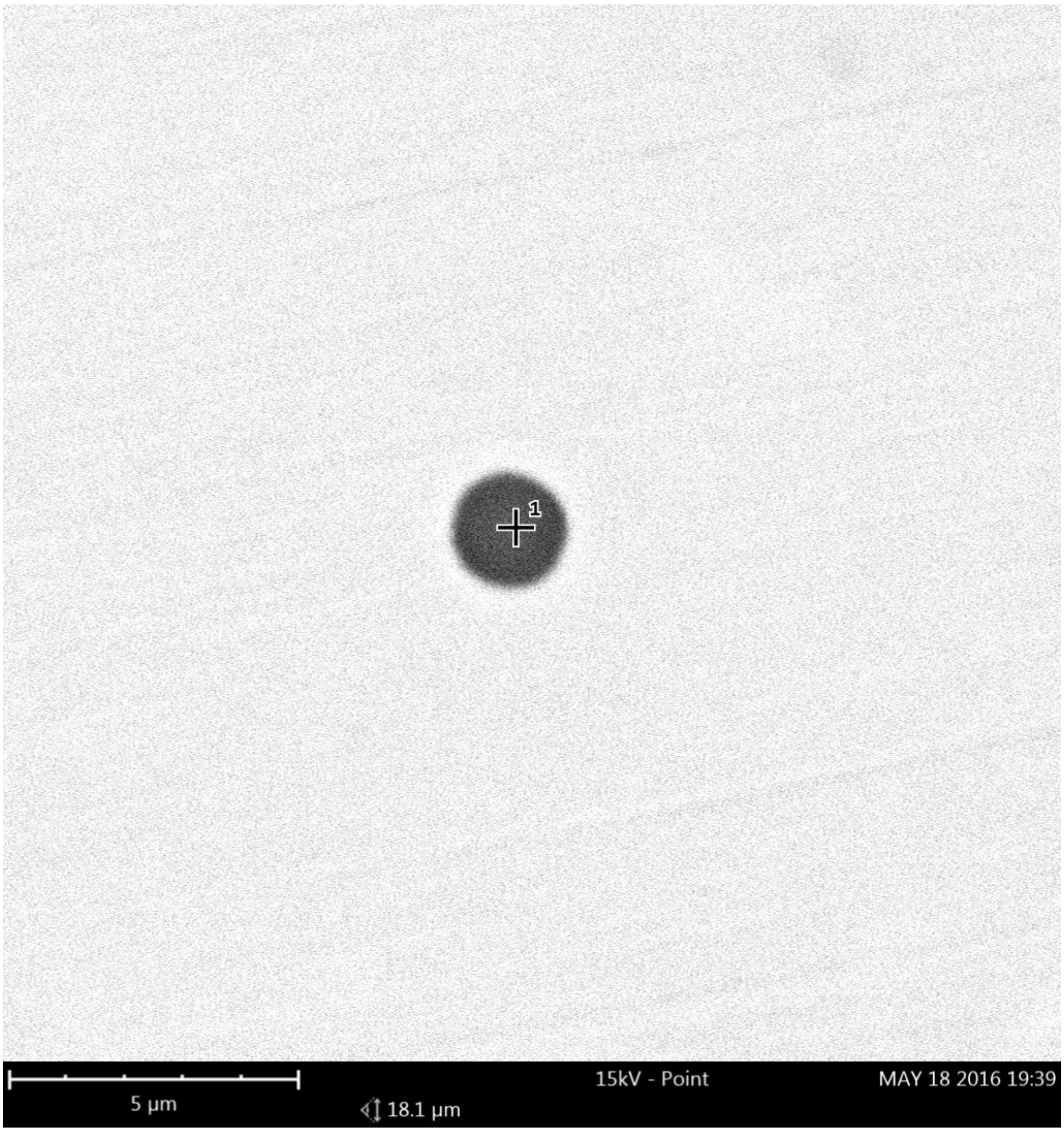

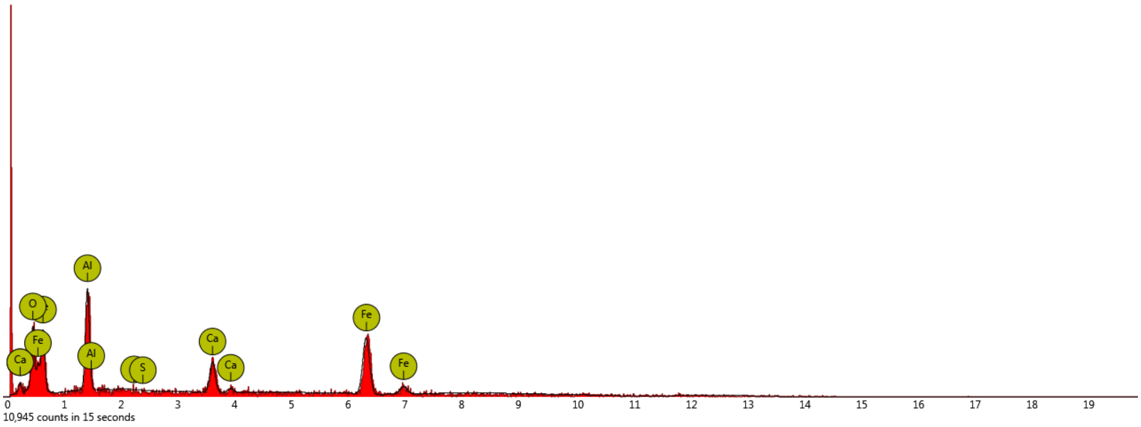

Disabled elements: As, B, Ba, Br, Cs, Dy, Er, F, Ga, Hg, Lu, Pt, Sb, Te, Tm, Yb, Zr

Element Number	Element Symbol	Element Name	Weight Concentration	Error
13	Al	Aluminium	20.4	0.3
26	Fe	Iron	50.3	0.6
8	O	Oxygen	21.7	0.9
20	Ca	Calcium	7.6	0.5

16	S	Sulfur	0.0	1.4

SEM-EDS results of sample A2C3-360s

Image 1

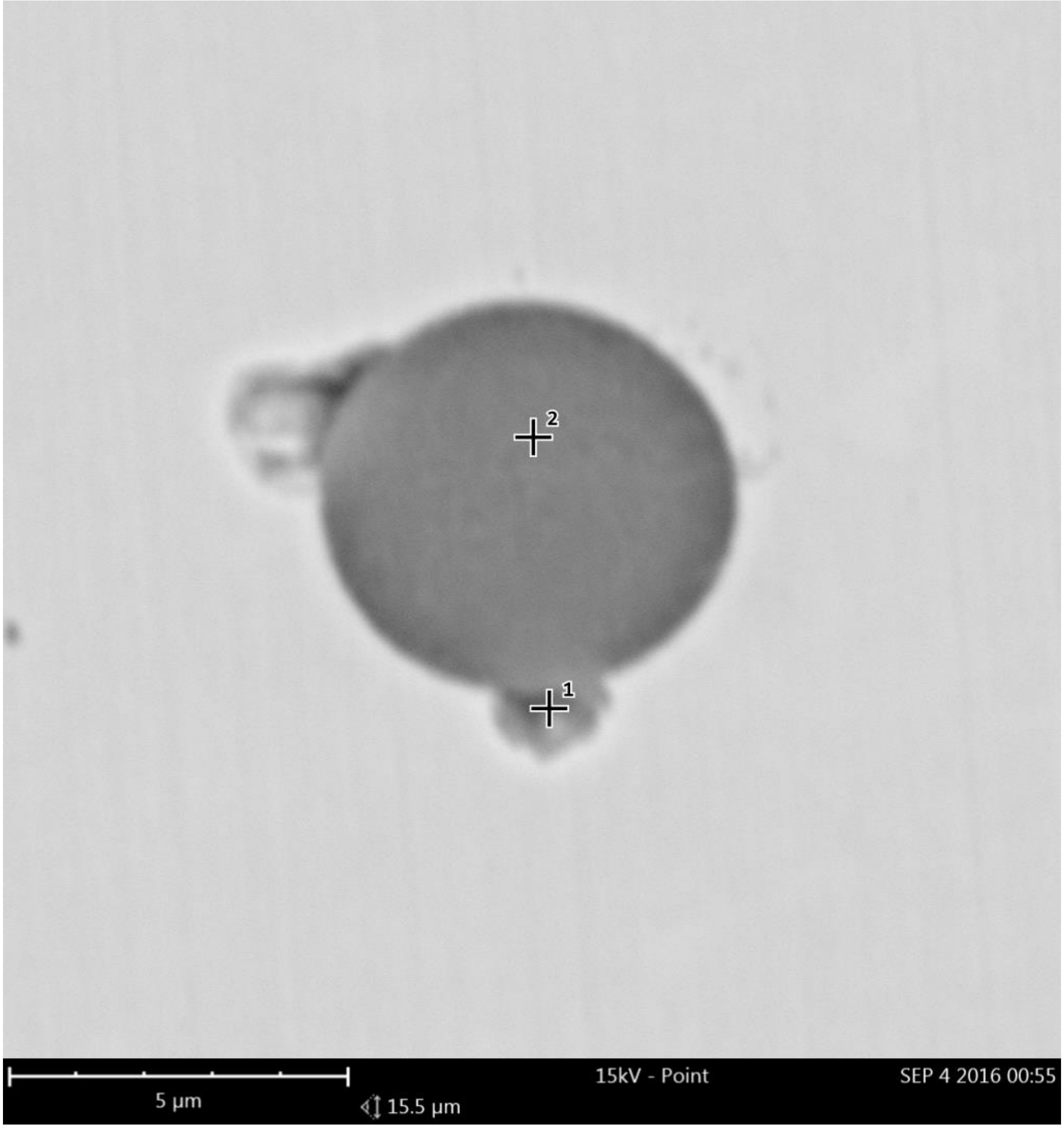

1. spot

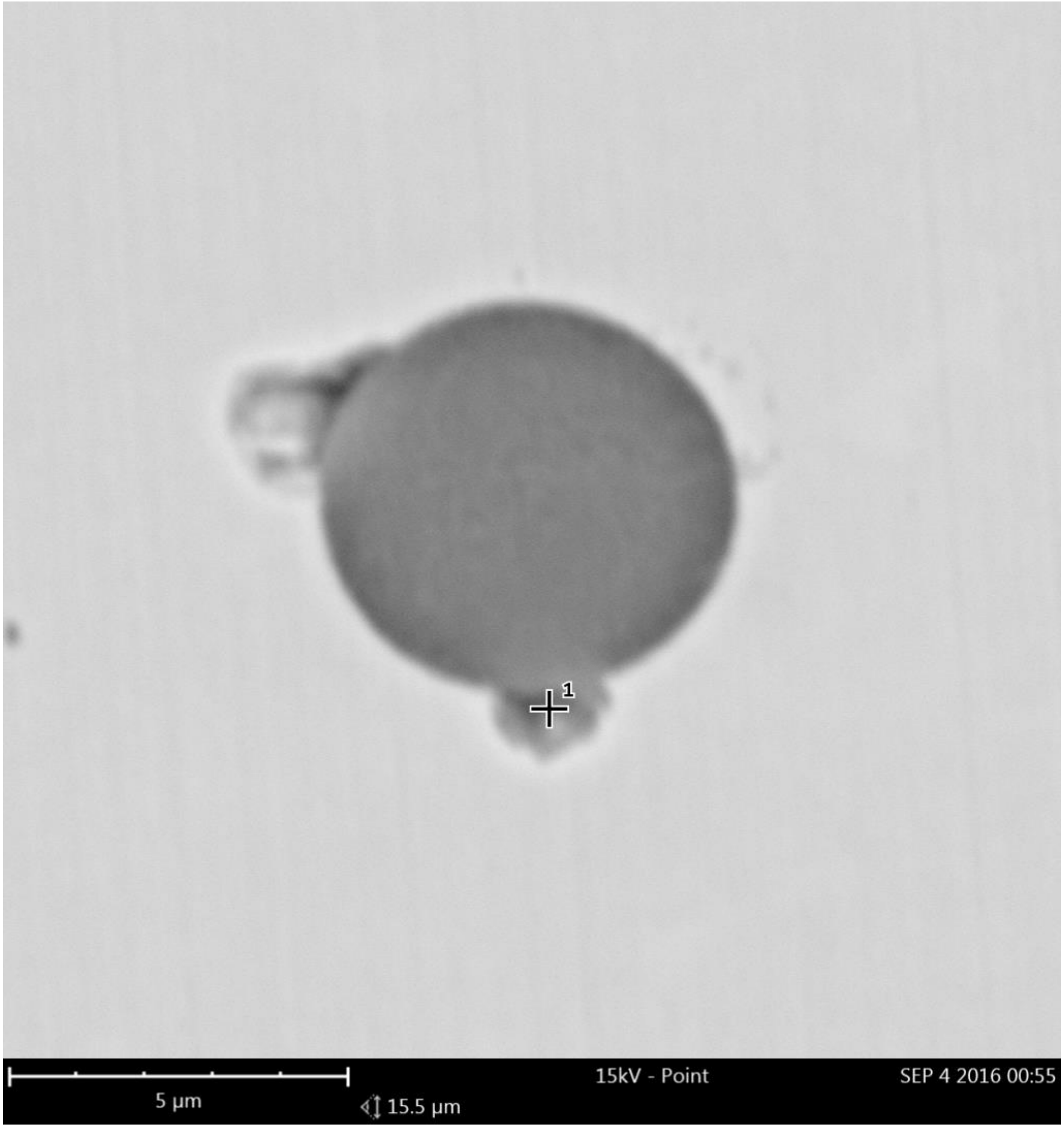

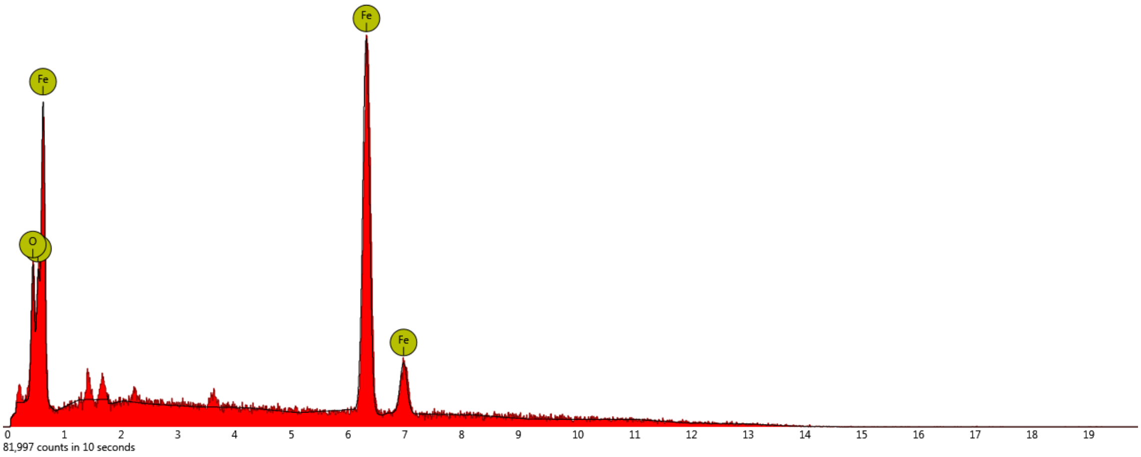

Disabled elements: As, B, Ba, Br, C, Cs, Dy, Er, F, Ga, Hg, La, Lu, Pb, Pm, Pt, Rb, Sb, Sr, Te, Tm, Yb, Zr

Element Number	Element Symbol	Element Name	Weight Concentration	Error
26	Fe	Iron	91.1	0.4
8	O	Oxygen	8.9	0.6

2. spot

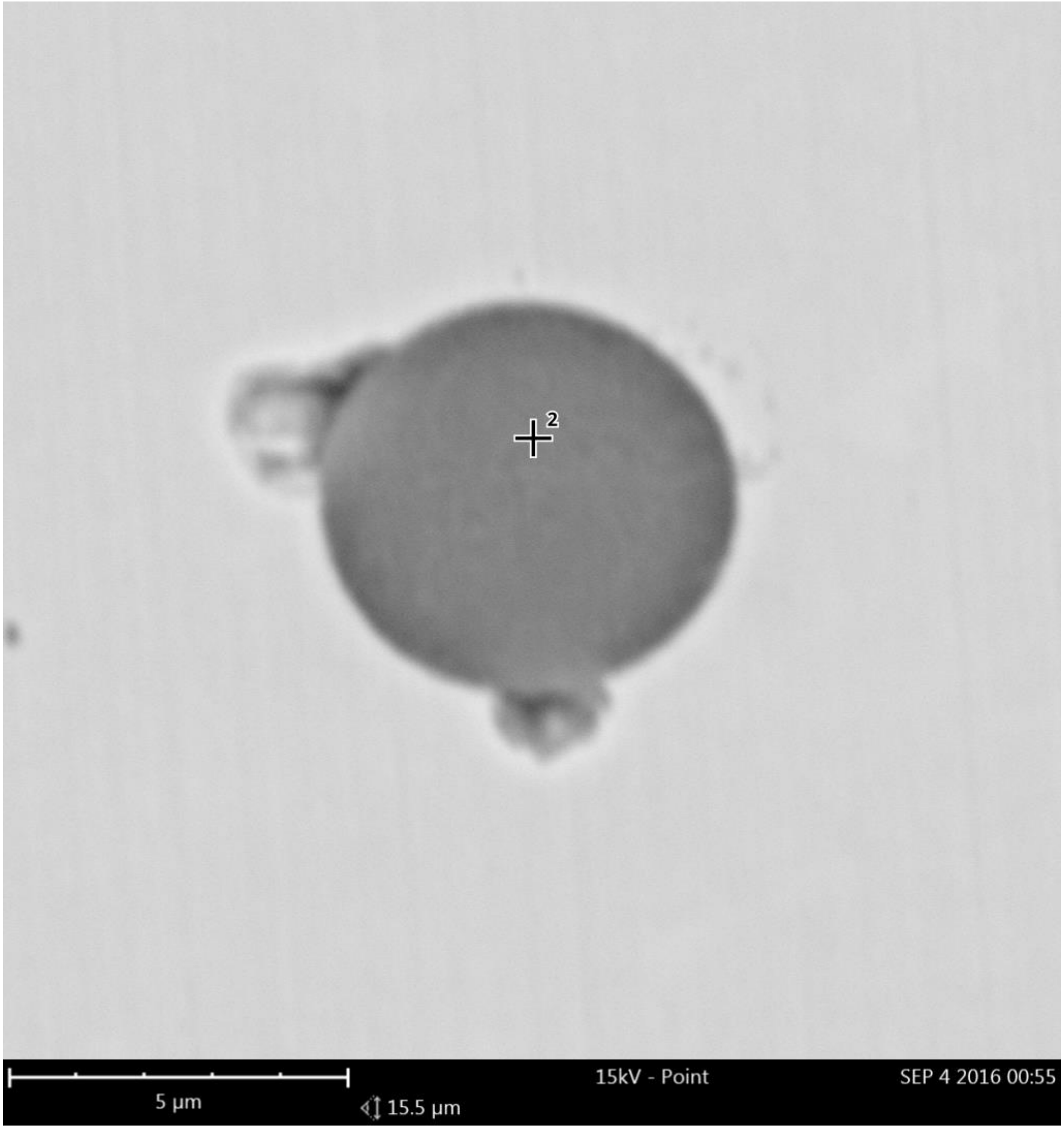

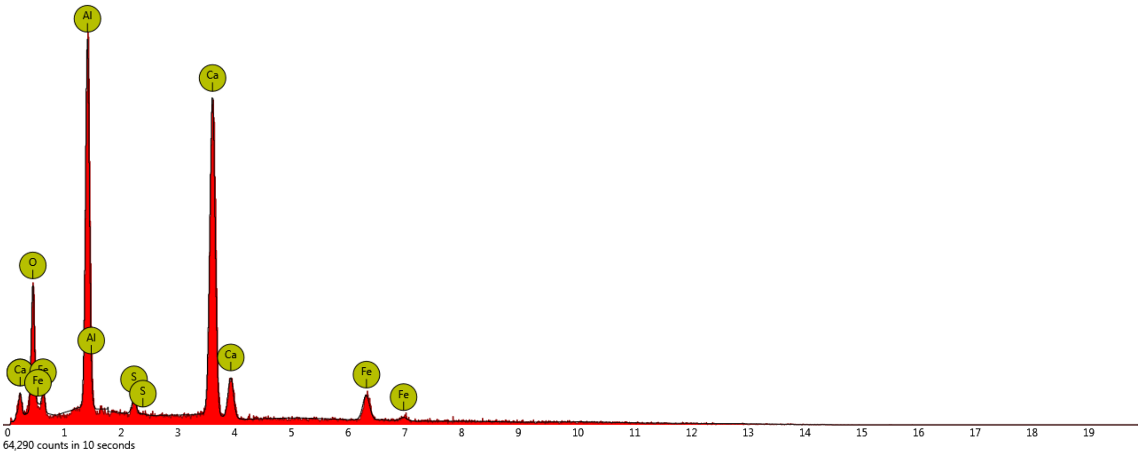

Disabled elements: As, B, Ba, Br, C, Cs, Dy, Er, F, Ga, Hg, La, Lu, Pb, Pm, Pt, Rb, Sb, Sr, Te, Tm, Yb, Zr

Element Number	Element Symbol	Element Name	Weight Concentration	Error
20	Ca	Calcium	31.4	0.4
13	Al	Aluminium	22.7	0.2

8	O	Oxygen	35.8	0.5
26	Fe	Iron	9.1	0.5
16	S	Sulfur	1.0	0.7

Image 2

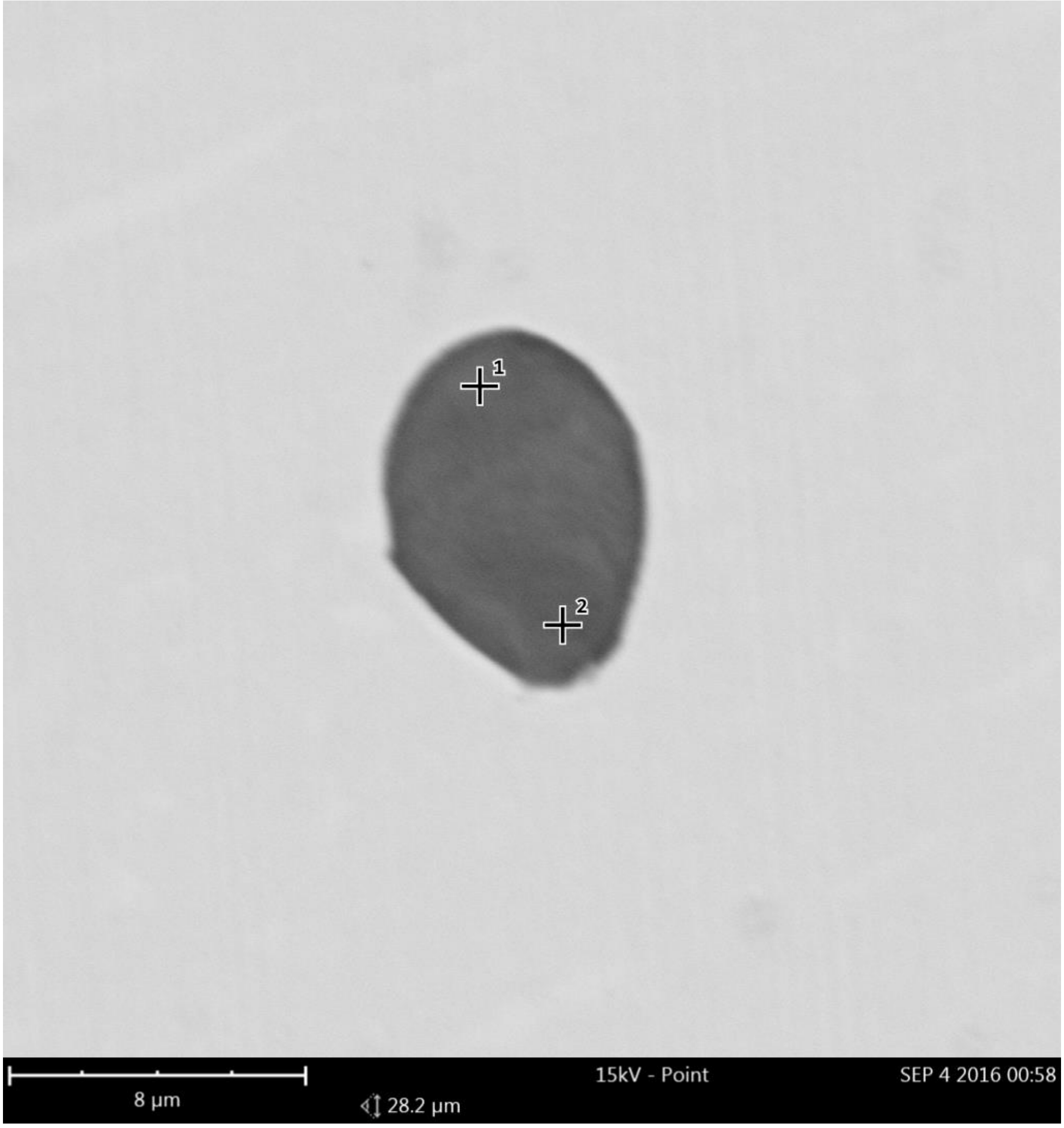

1. spot

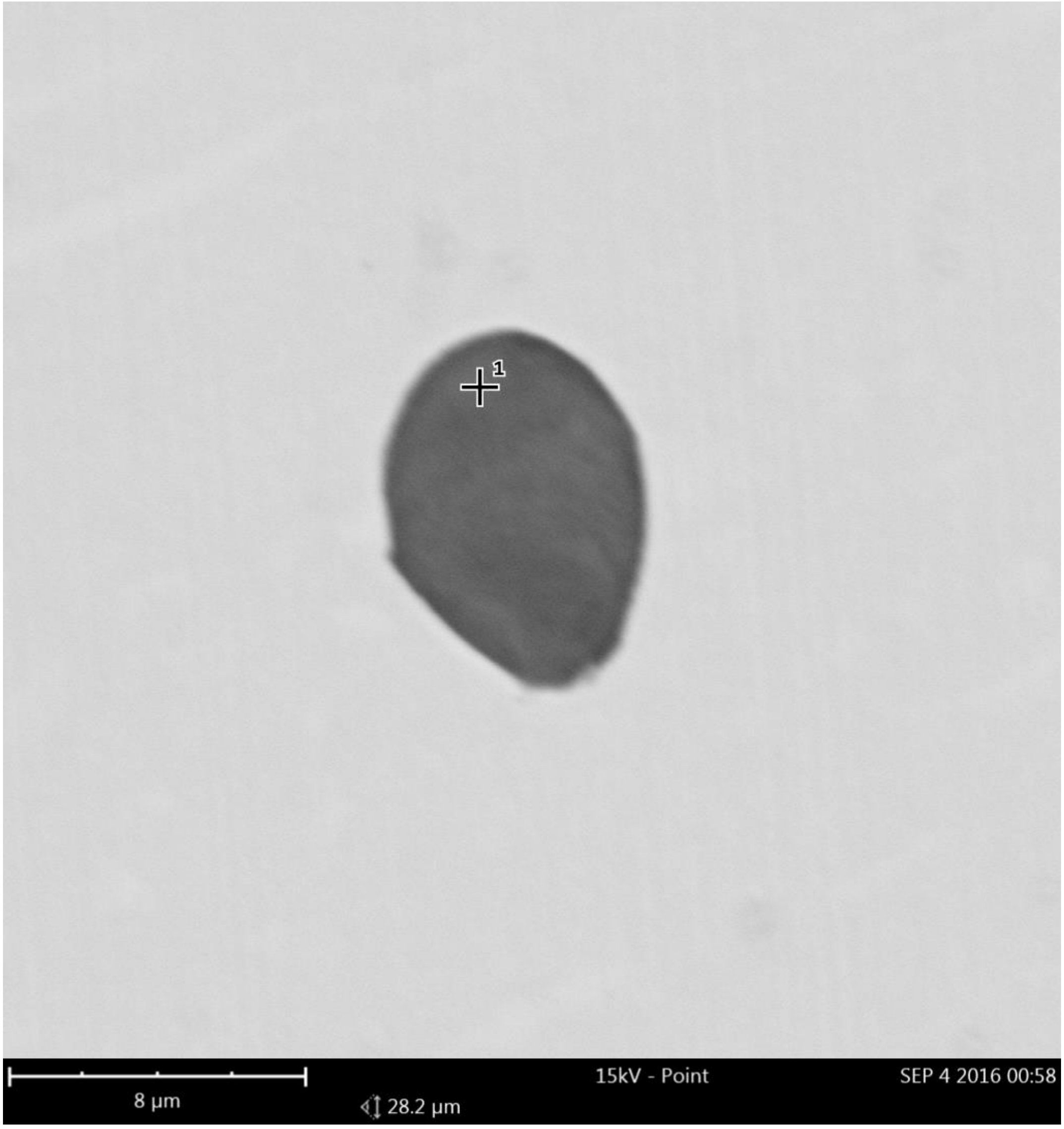

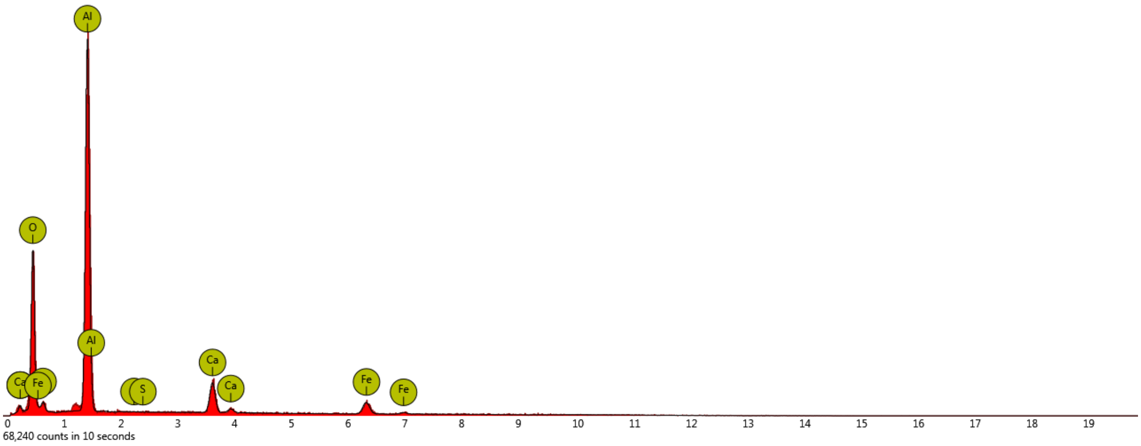

Disabled elements: As, B, Ba, Br, C, Cs, Dy, Er, F, Ga, Hg, La, Lu, Pb, Pm, Pt, Rb, Sb, Sr, Te, Tm, Yb, Zr

**Element Number**	**Element Symbol**	**Element Name**	**Weight Concentration**	**Error**
13	Al	Aluminium	40.8	0.2
8	O	Oxygen	44.9	0.5
20	Ca	Calcium	6.0	0.4
26	Fe	Iron	8.3	0.5

16	S	Sulfur	0.0	1.0

2. spot

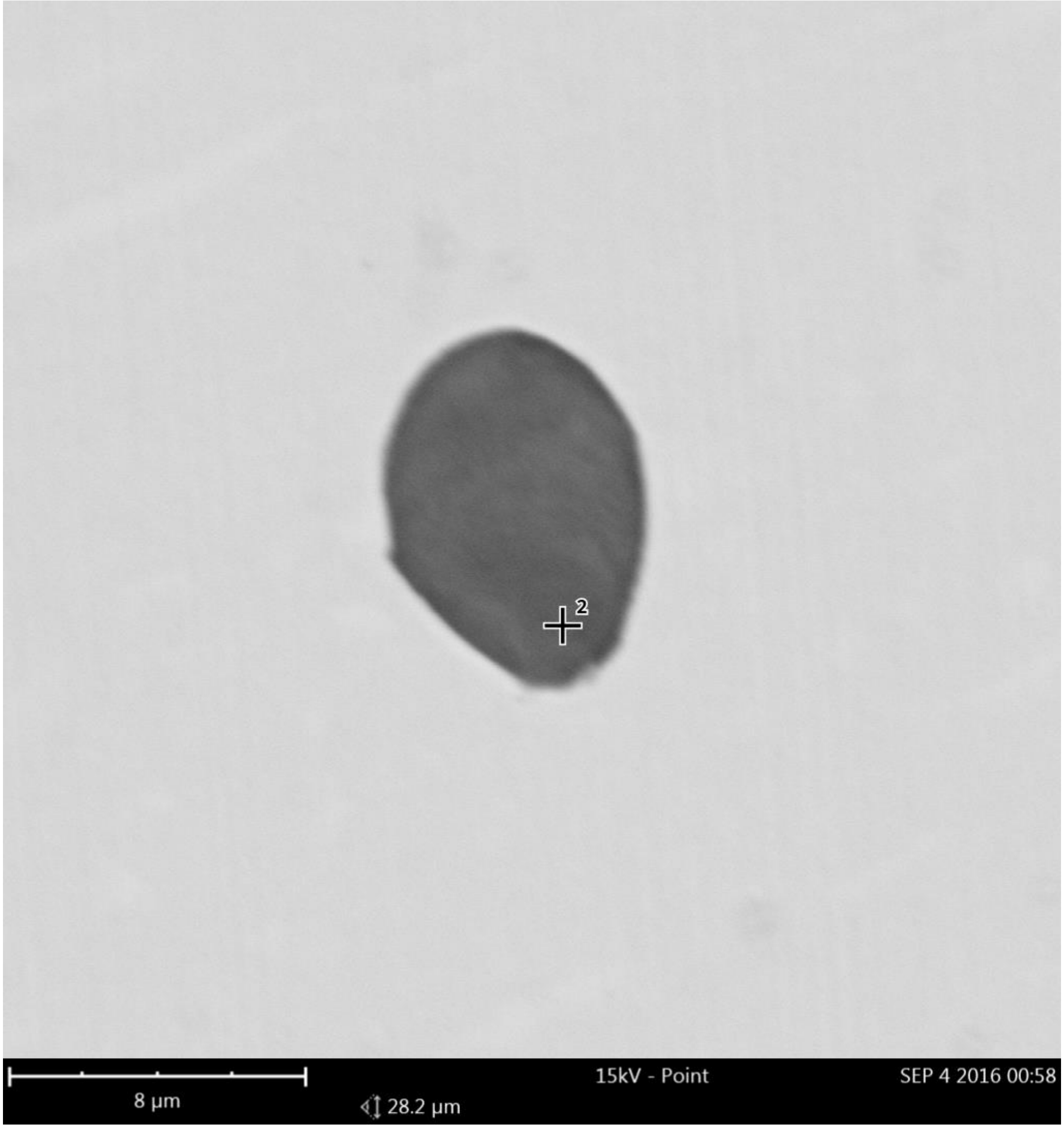

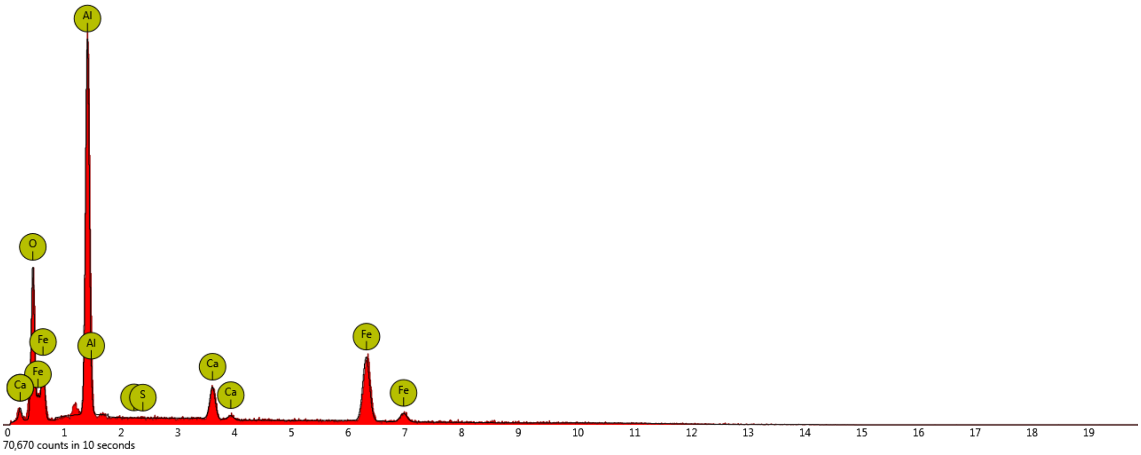

Disabled elements: As, B, Ba, Br, C, Cs, Dy, Er, F, Ga, Hg, La, Lu, Pb, Pm, Pt, Rb, Sb, Sr, Te, Tm, Yb, Zr

Element Number	Element Symbol	Element Name	Weight Concentration	Error

13	Al	Aluminium	35.1	0.1
8	O	Oxygen	28.5	0.7
26	Fe	Iron	31.9	0.5
20	Ca	Calcium	4.5	0.4
16	S	Sulfur	0.0	1.4

Image 3

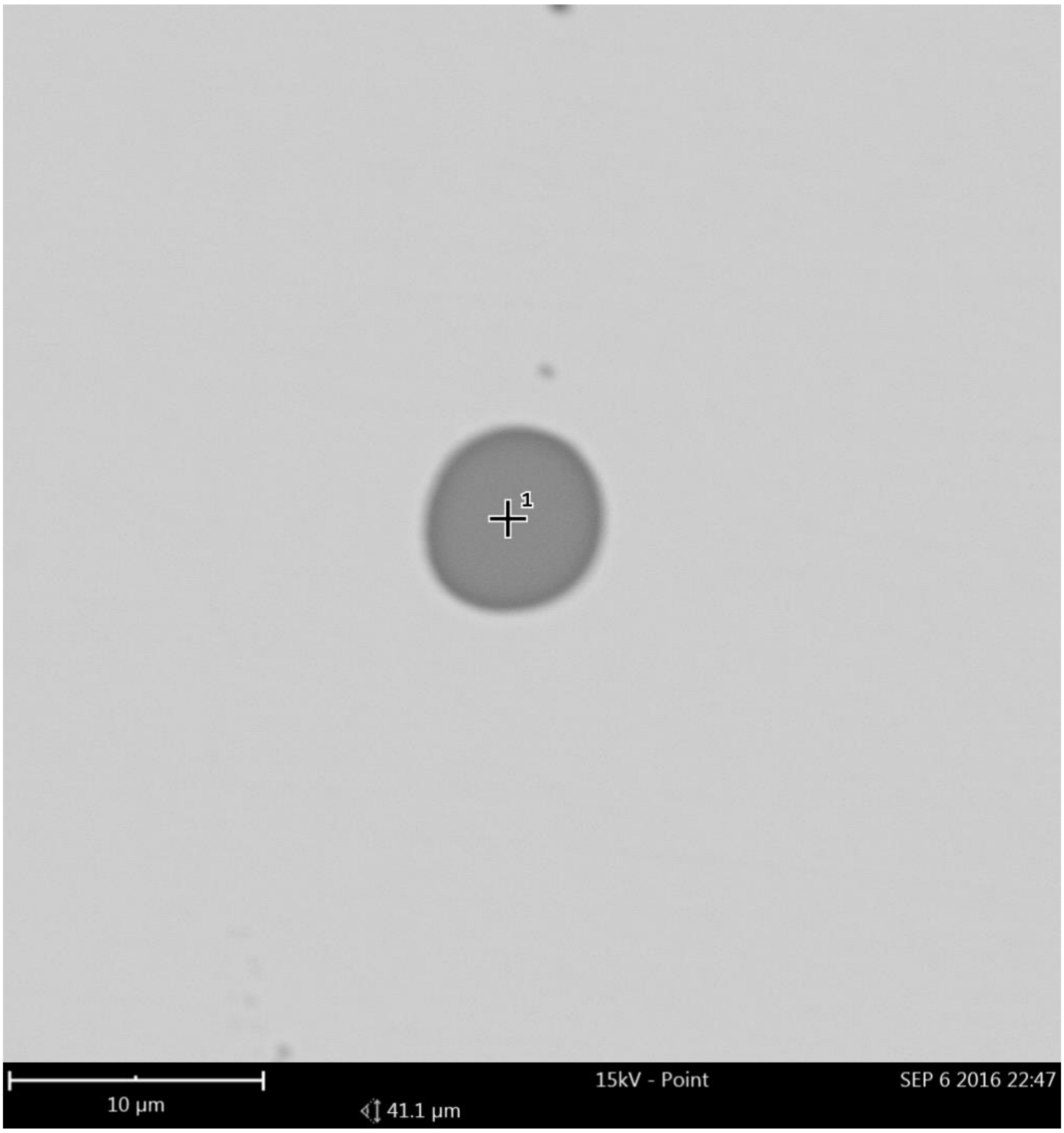

1. spot

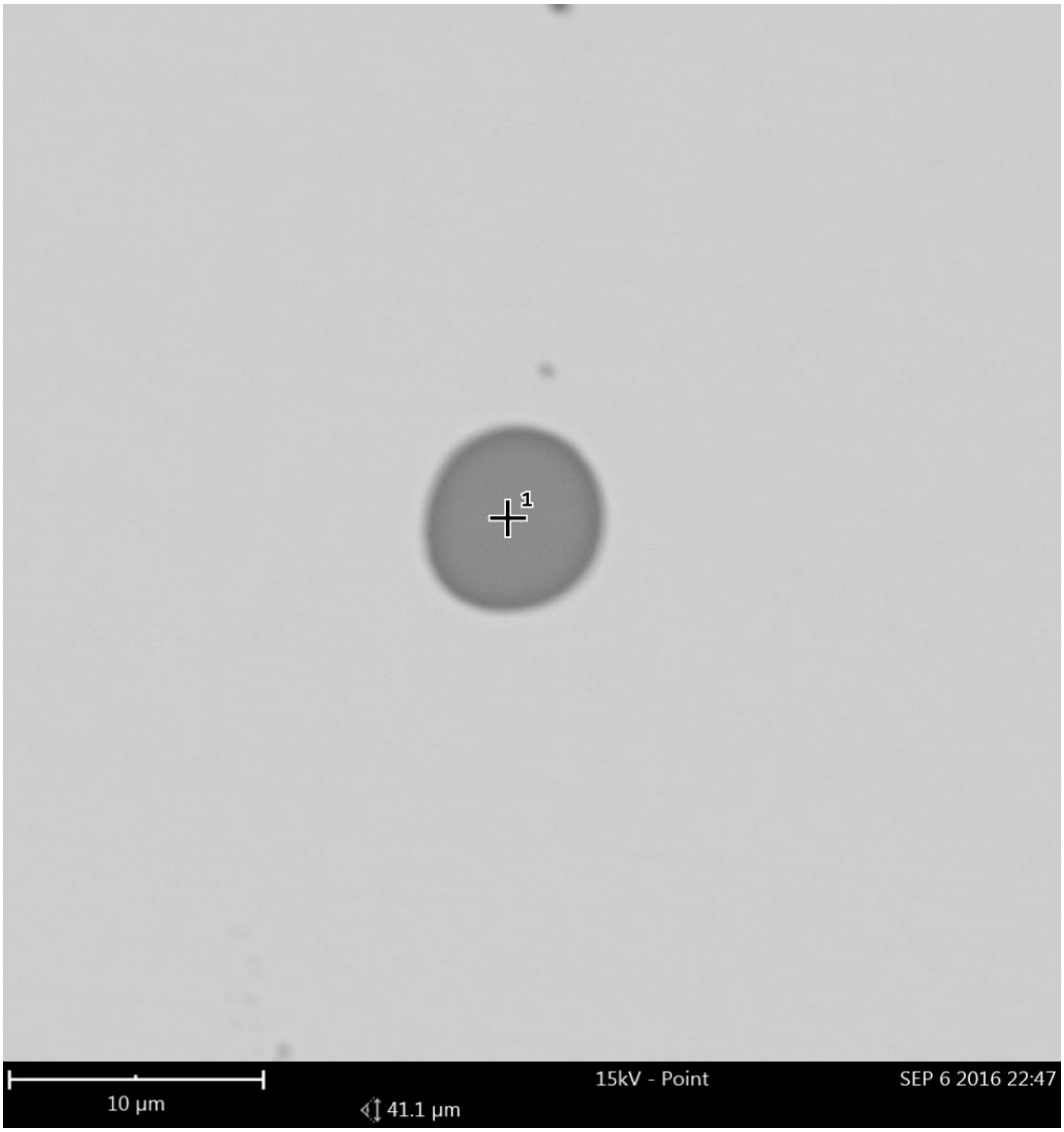

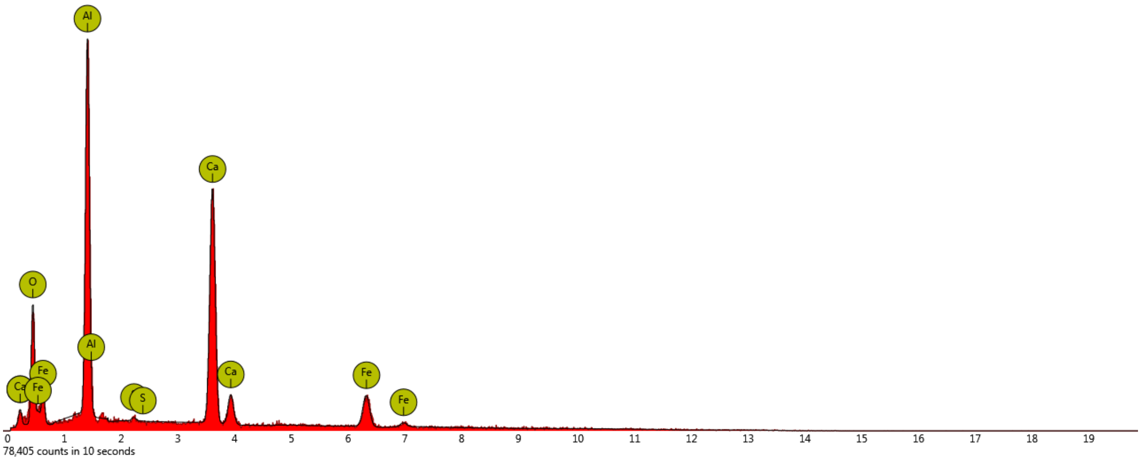

Disabled elements: As, B, Ba, Br, C, Cs, Dy, Er, F, Ga, Hg, La, Lu, Pb, Pm, Pt, Rb, Sb, Si, Sr, Te, Tm, Yb, Zr

Element Number	Element Symbol	Element Name	Weight Concentration	Error
13	Al	Aluminium	26.4	0.1
20	Ca	Calcium	26.3	0.1
8	O	Oxygen	34.0	0.4

26	Fe	Iron	13.0	0.4
16	S	Sulfur	0.3	0.7

Image 4

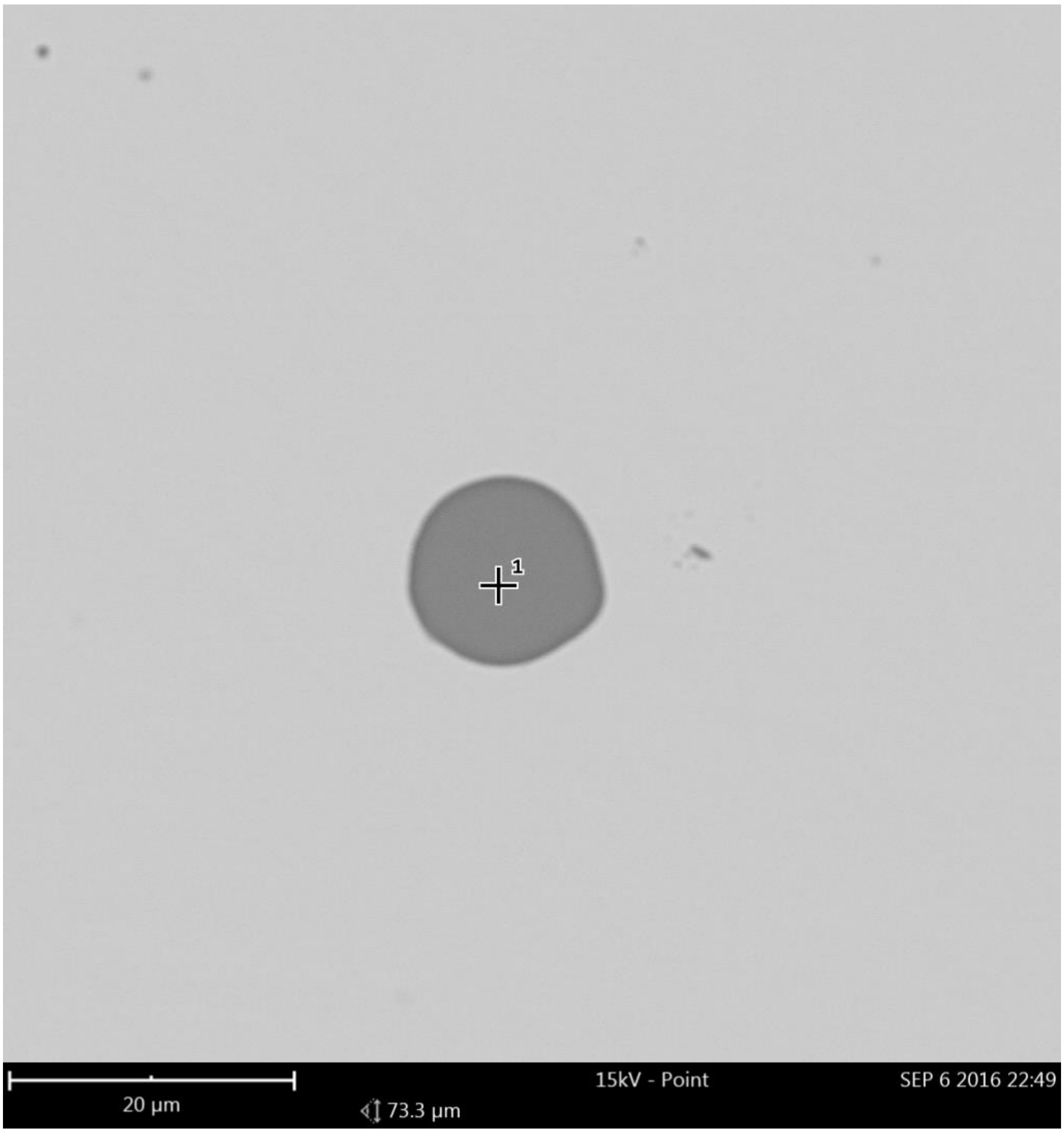

1. spot

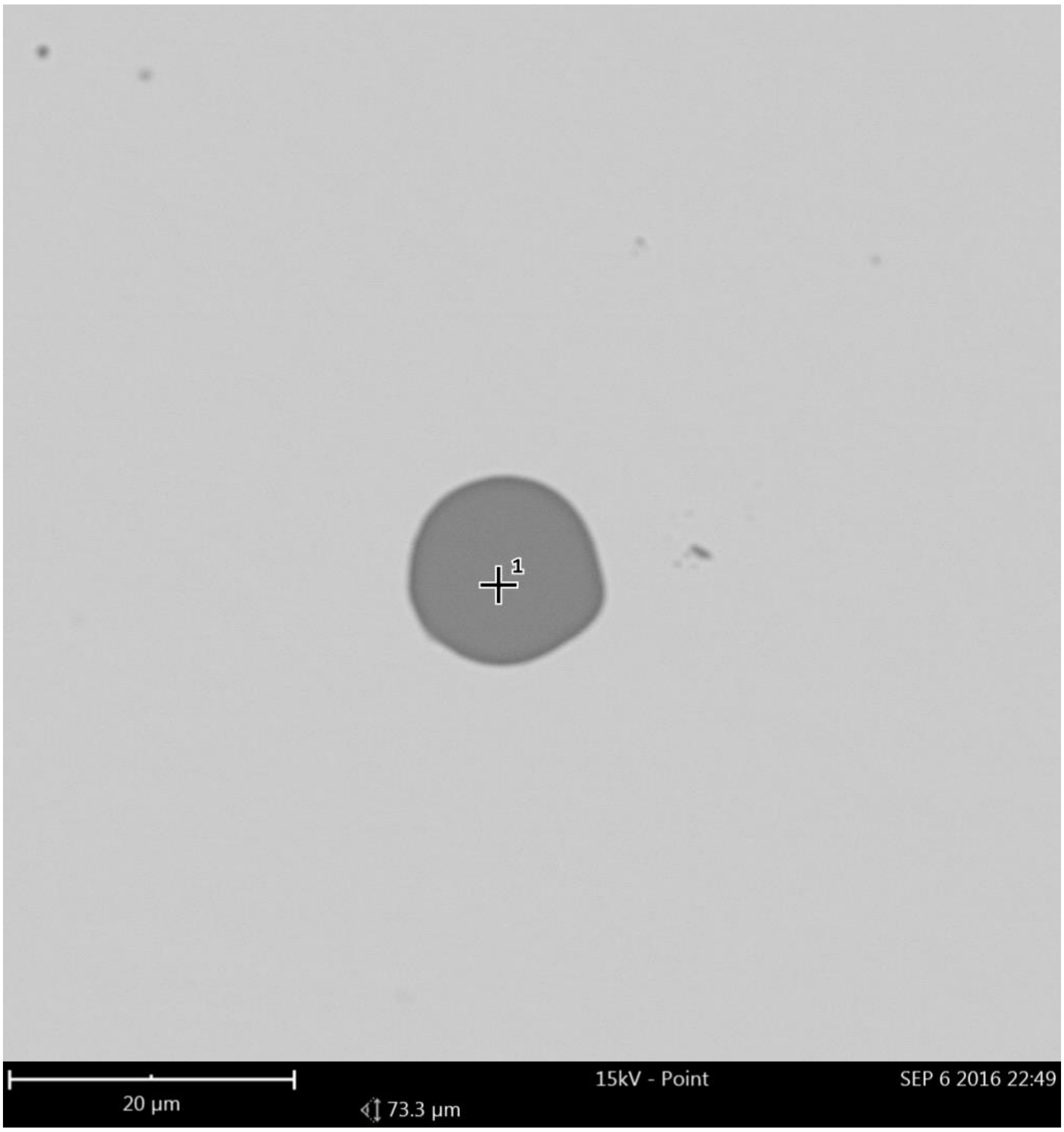

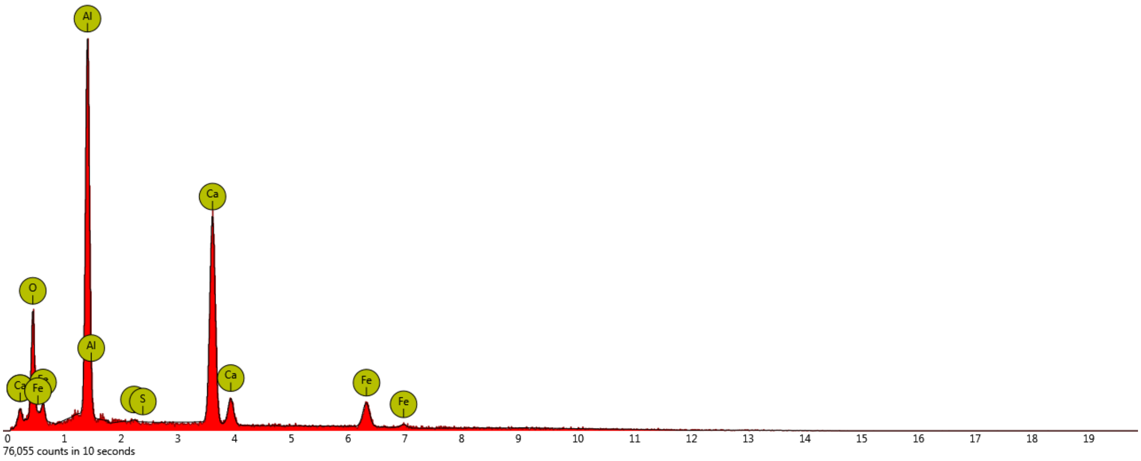

Disabled elements: As, B, Ba, Br, C, Cs, Dy, Er, F, Ga, Hg, La, Lu, Pb, Pm, Pt, Rb, Sb, Si, Sr, Te, Tm, Yb, Zr

Element Number	Element Symbol	Element Name	Weight Concentration	Error
13	Al	Aluminium	28.8	0.1
20	Ca	Calcium	25.9	0.1
8	O	Oxygen	34.3	0.3

26	Fe	Iron	10.9	0.3
16	S	Sulfur	0.2	2.1

Image 5

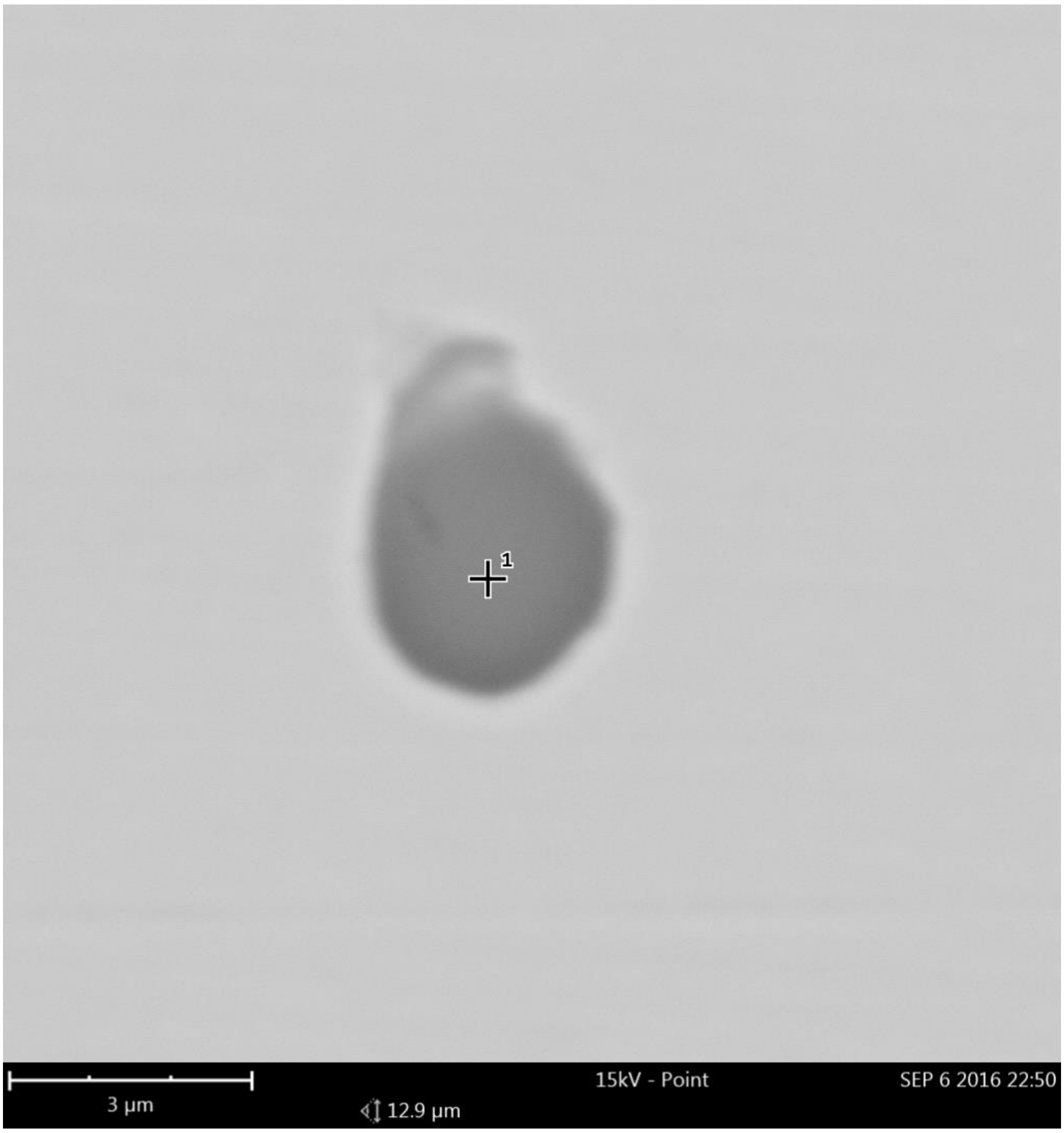

1. spot

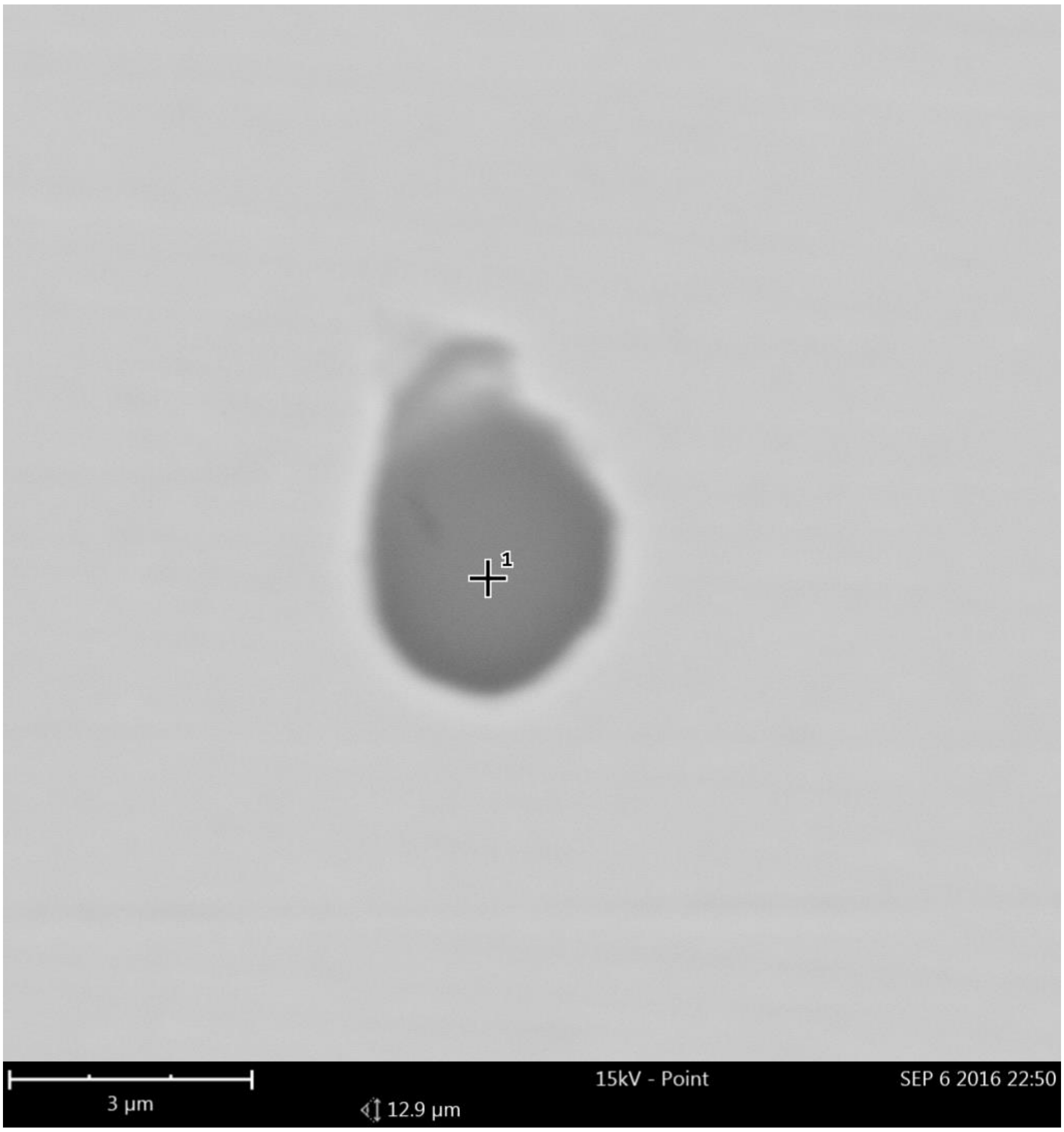

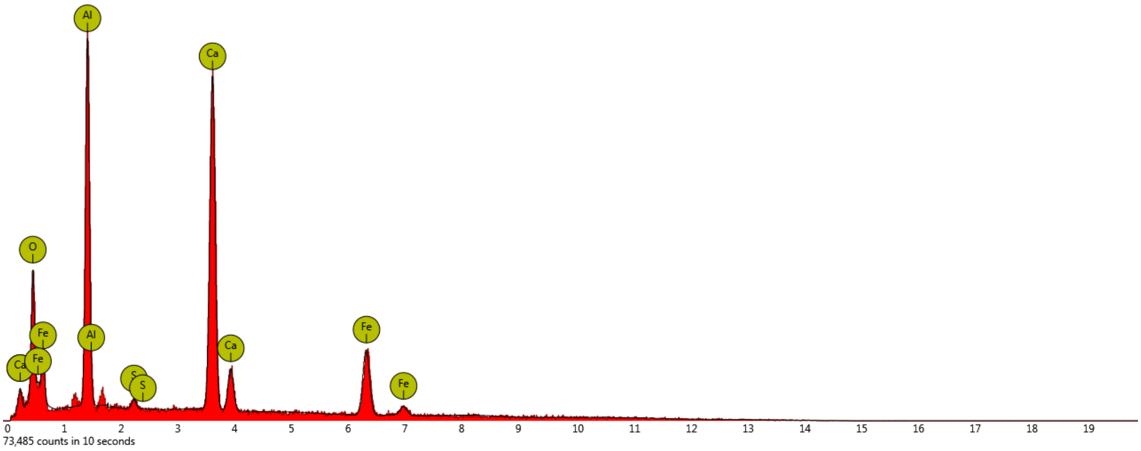

Disabled elements: As, B, Ba, Br, C, Cs, Dy, Er, F, Ga, Hg, La, Lu, Pb, Pm, Pt, Rb, Sb, Si, Sr, Te, Tm, Yb, Zr

Element Number	Element Symbol	Element Name	Weight Concentration	Error
20	Ca	Calcium	28.3	0.2
13	Al	Aluminium	21.1	0.1
8	O	Oxygen	29.4	0.6

26	Fe	Iron	20.5	0.5
16	S	Sulfur	0.6	1.0

SEM-EDS results of sample A2C3-1800s

Image 1

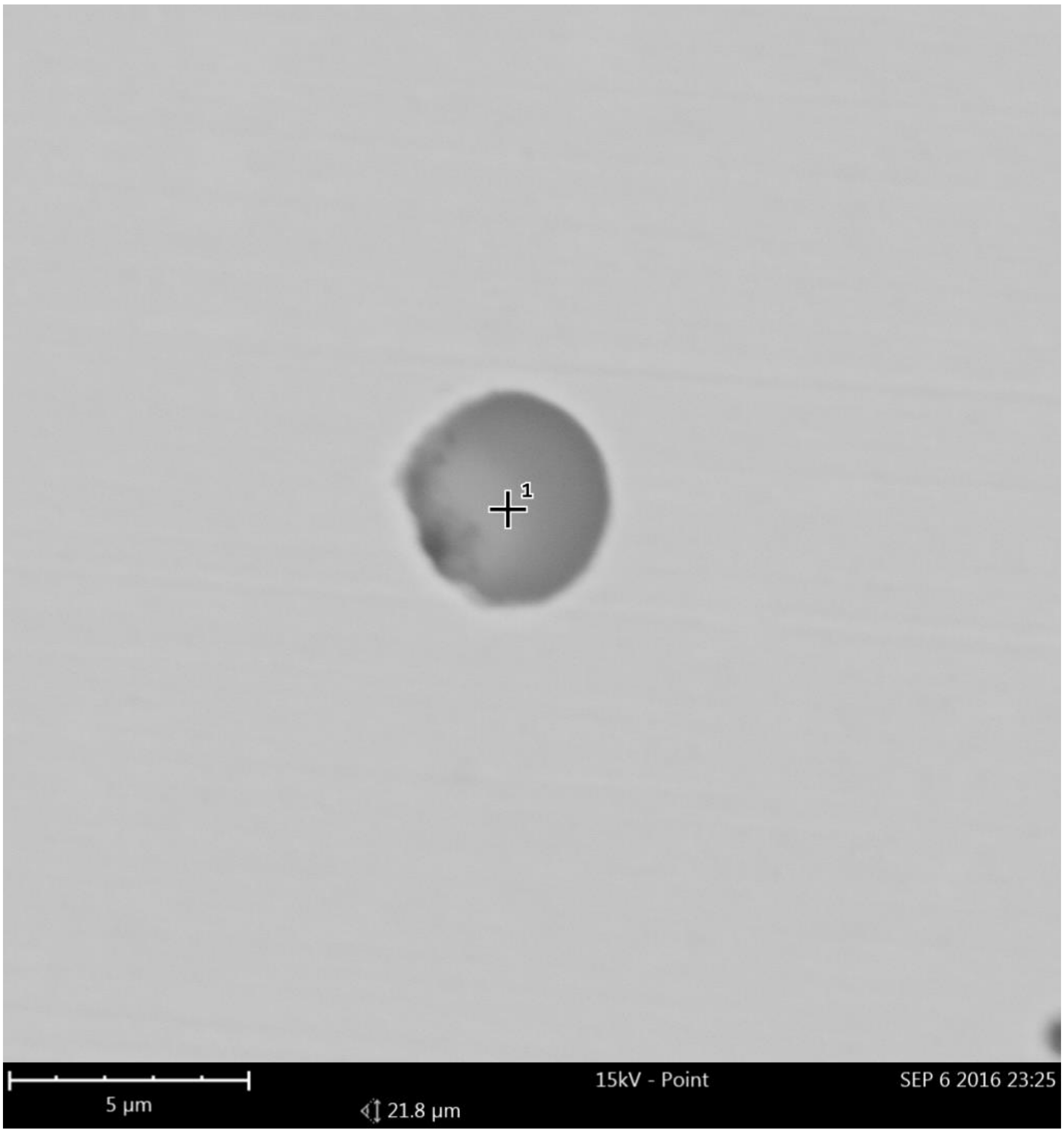

1. spot

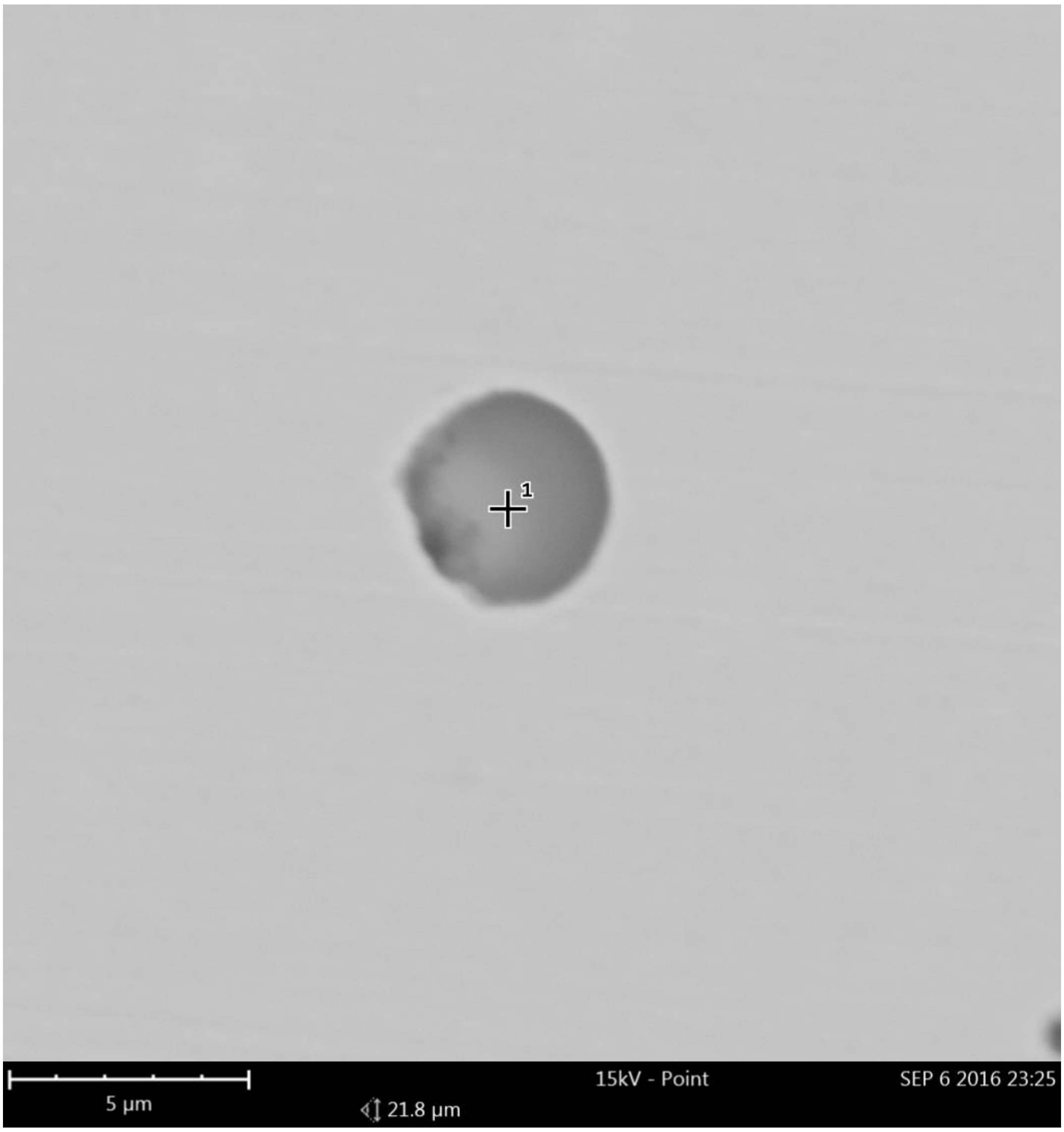

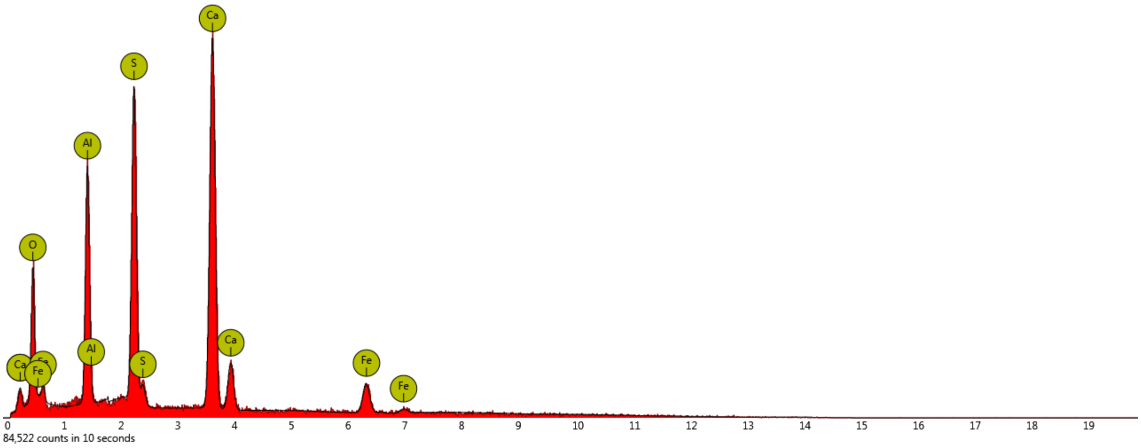

Disabled elements: As, B, Ba, Br, C, Cs, Dy, Er, F, Ga, Hg, La, Lu, Pb, Pm, Pt, Rb, Sb, Si, Sr, Te, Tm, Yb, Zr

Element Number	Element Symbol	Element Name	Weight Concentration	Error
20	Ca	Calcium	30.0	0.1
16	S	Sulfur	16.0	0.3
13	Al	Aluminium	11.6	0.1

8	O	Oxygen	33.8	0.6
26	Fe	Iron	8.6	0.4

Image 2

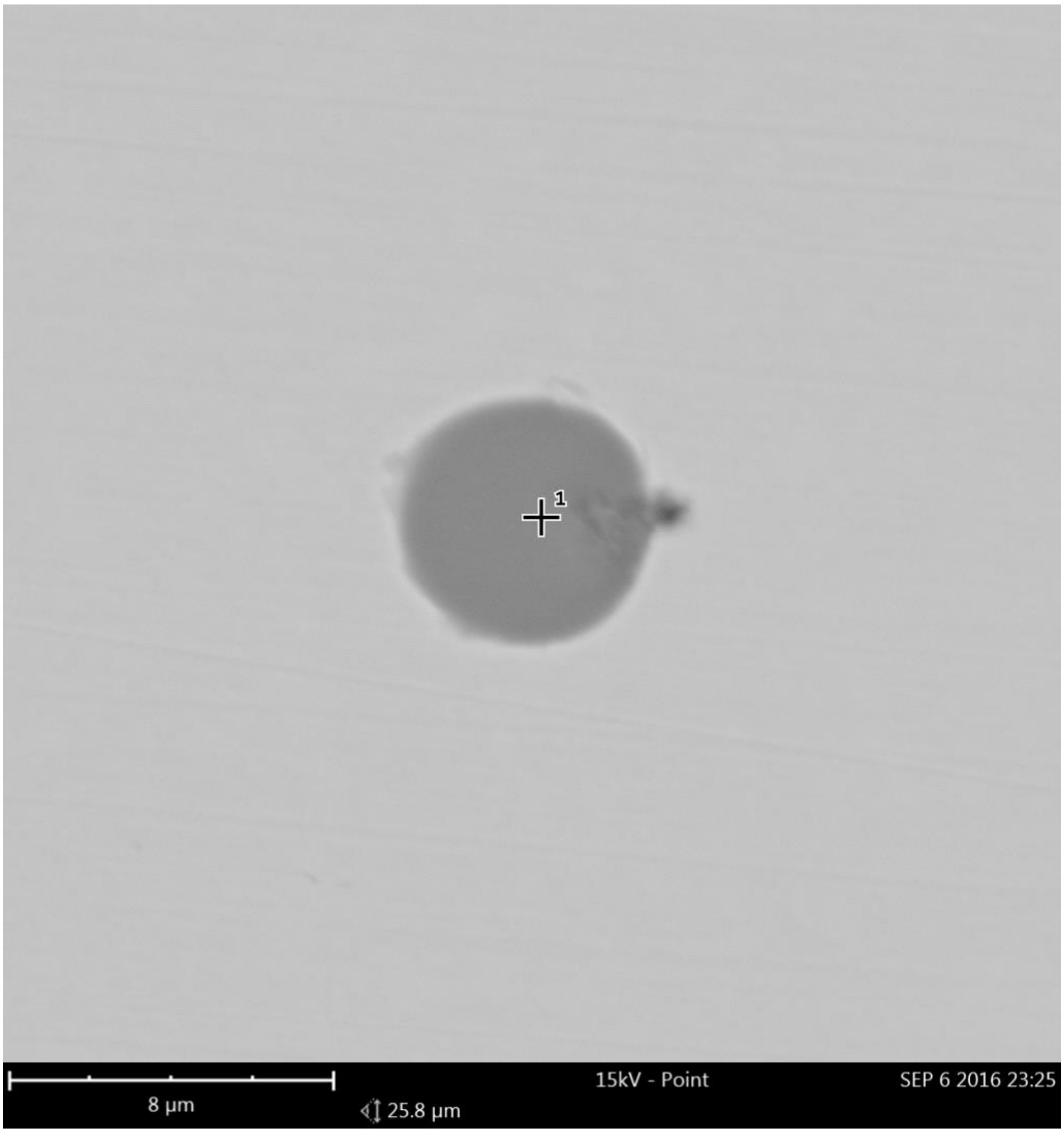

1. spot

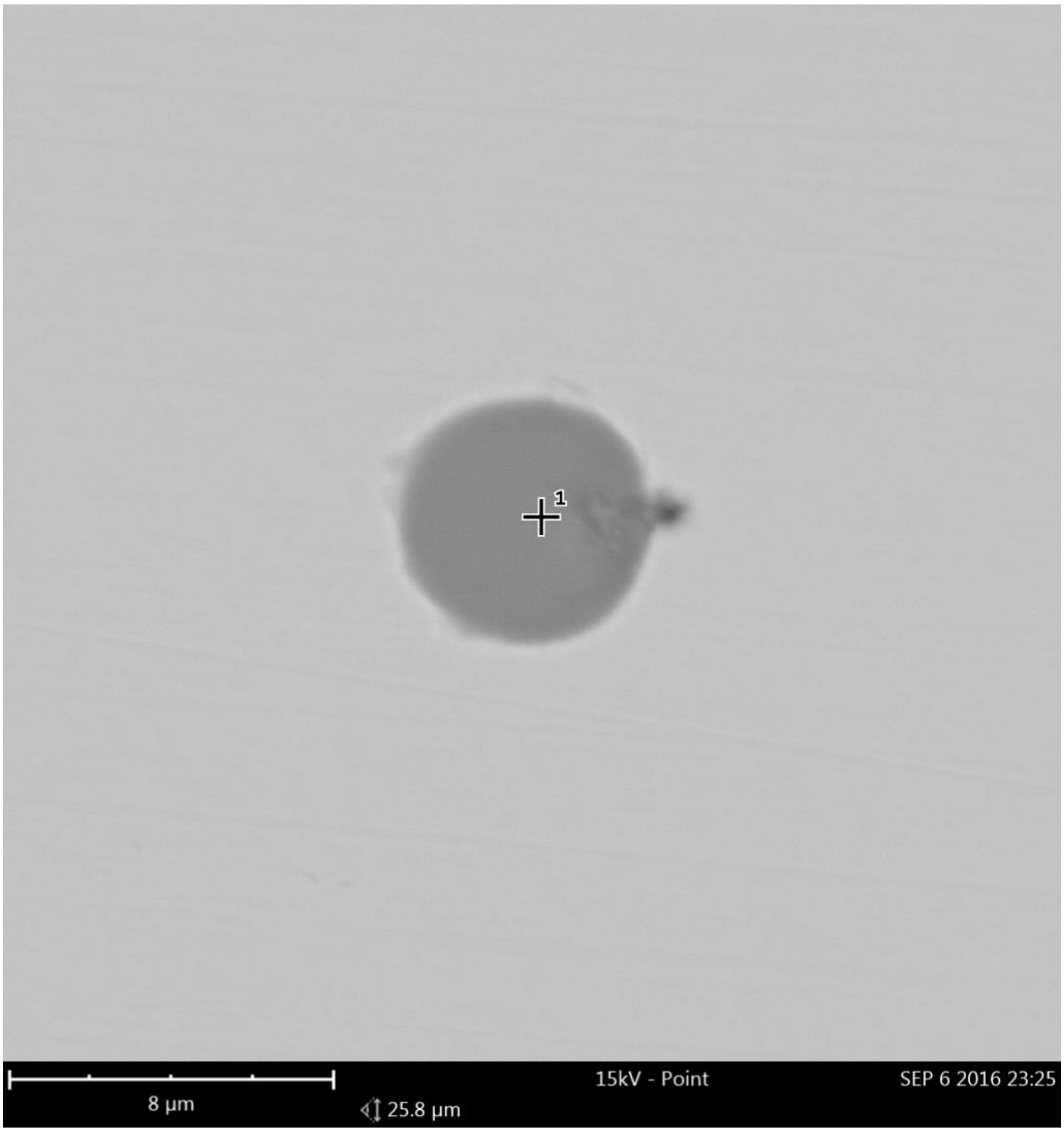

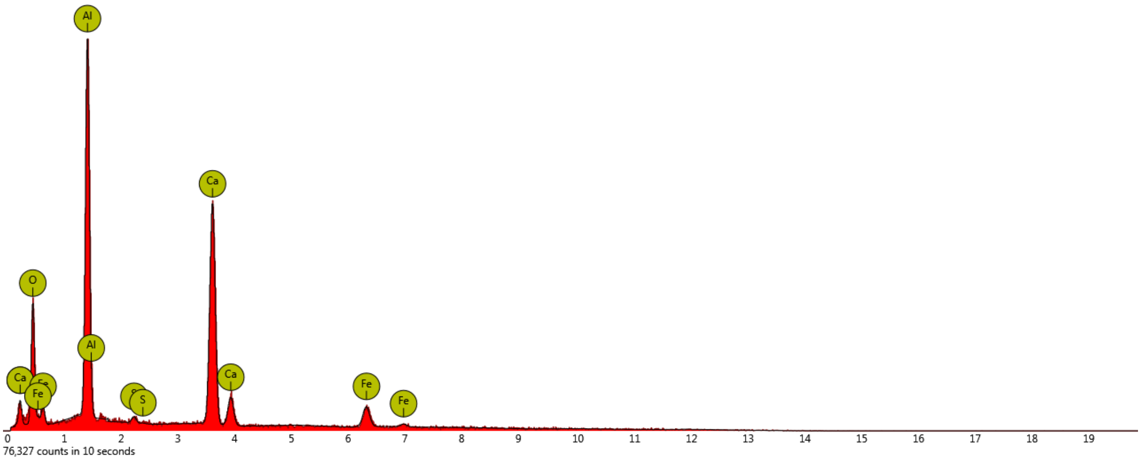

Disabled elements: As, B, Ba, Br, C, Cs, Dy, Er, F, Ga, Hg, La, Lu, Pb, Pm, Pt, Rb, Sb, Si, Sr, Te, Tm, Yb, Zr

**Element Number**	**Element Symbol**	**Element Name**	**Weight Concentration**	**Error**
13	Al	Aluminium	27.3	0.1
20	Ca	Calcium	26.3	0.2
8	O	Oxygen	37.0	0.3

26	Fe	Iron	8.8	0.4
16	S	Sulfur	0.5	1.9

Image 3

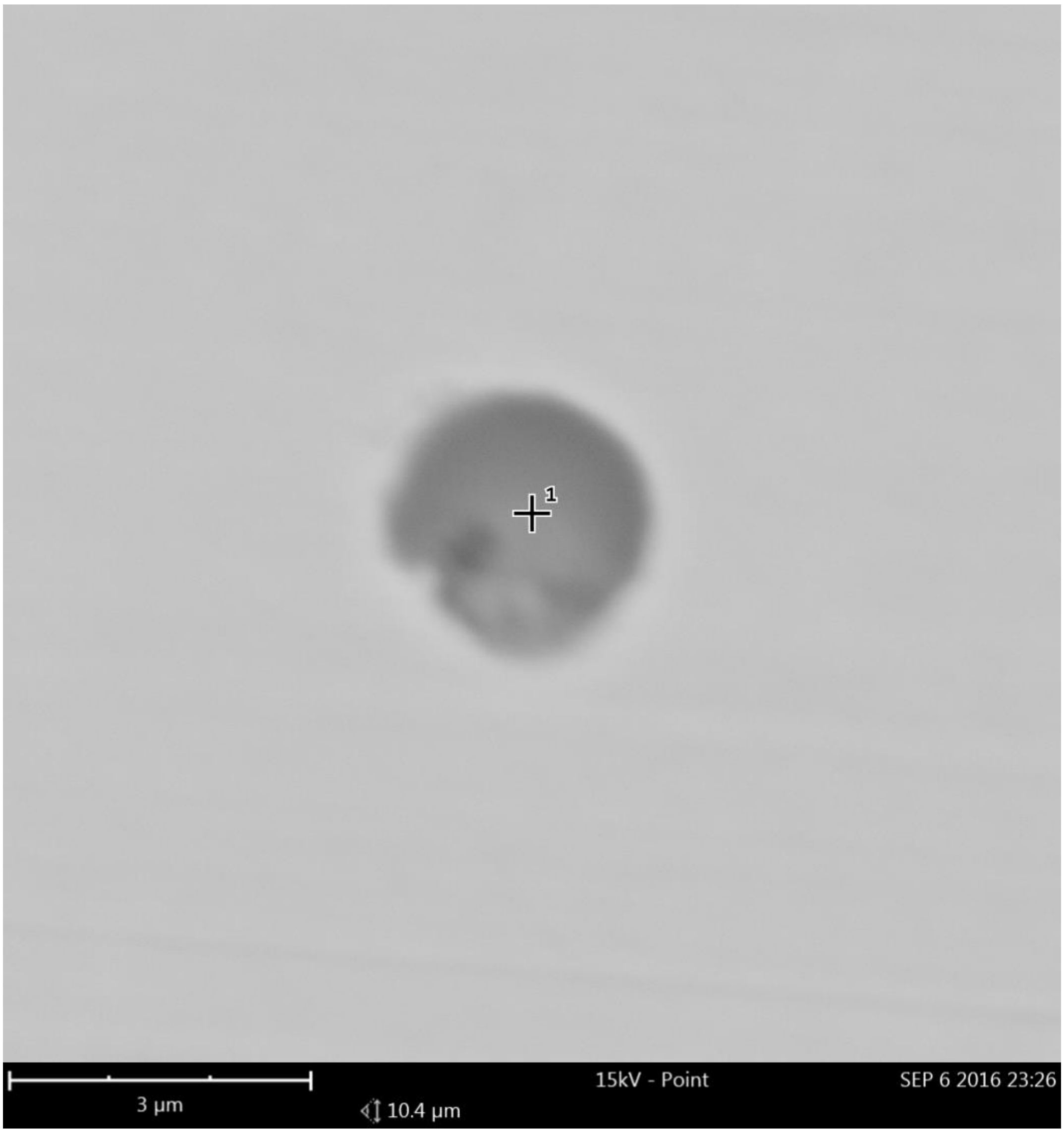

1. spot

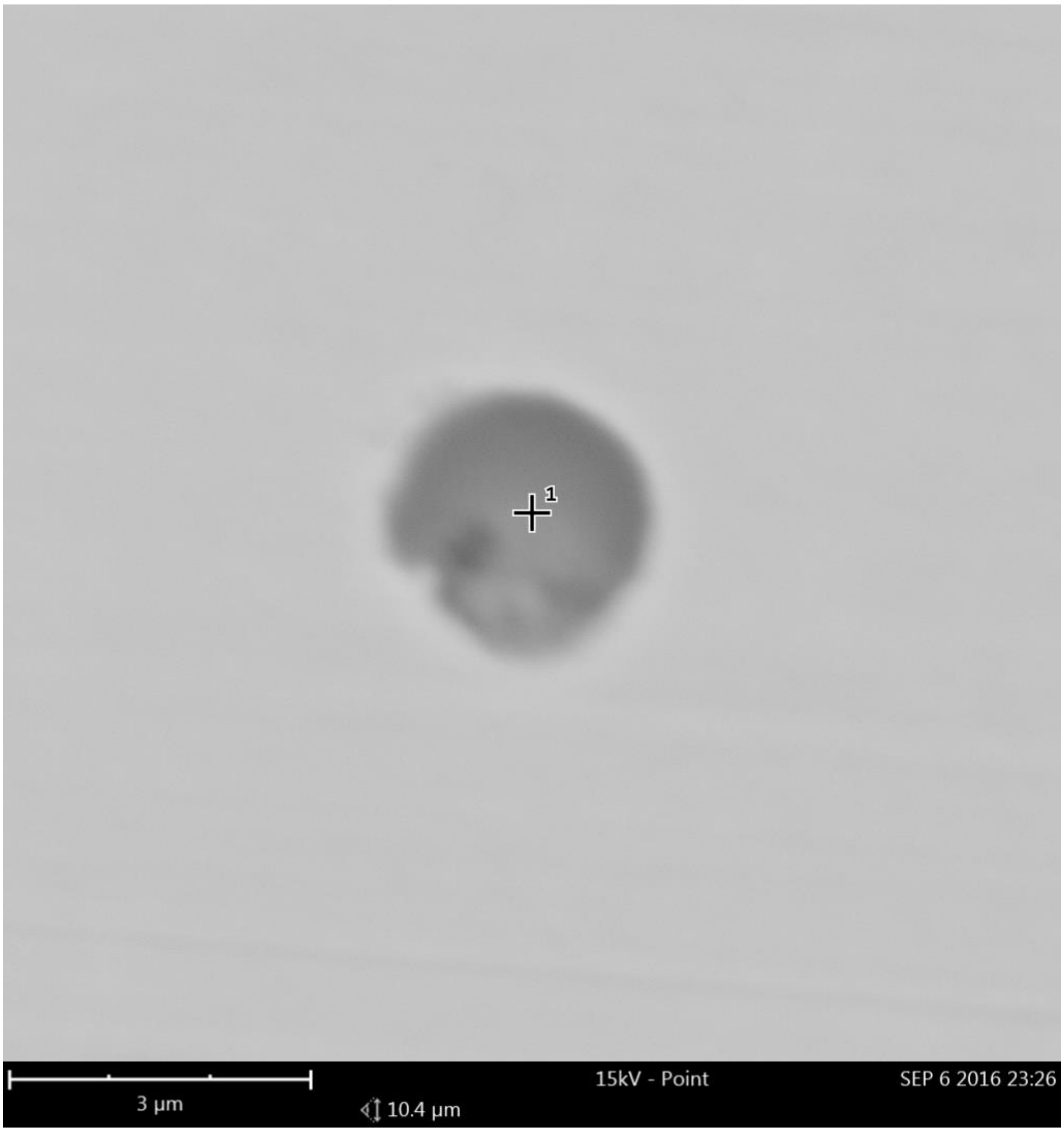

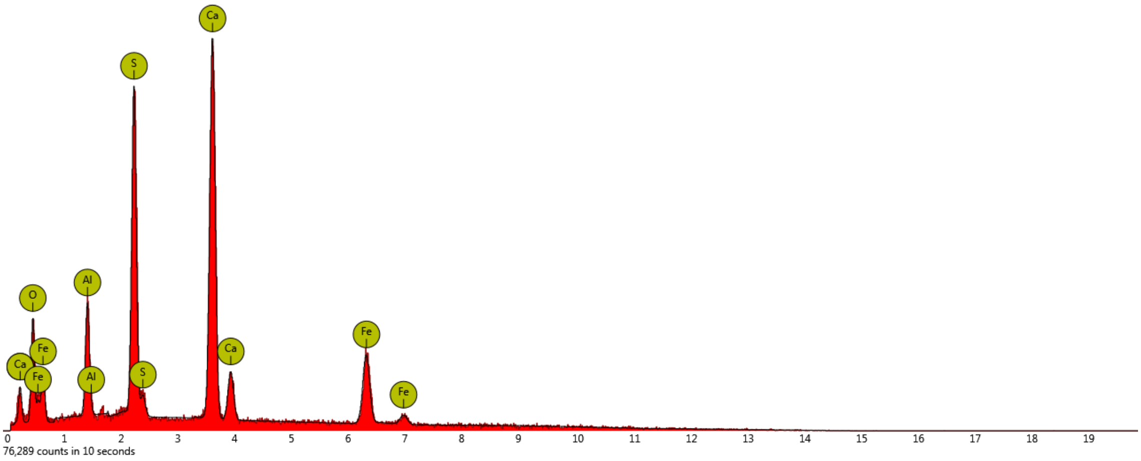

Disabled elements: As, B, Ba, Bi, Br, C, Cs, Dy, Er, F, Ga, Hg, La, Lu, Pb, Pm, Pt, Rb, Sb, Si, Sr, Te, Tm, Yb, Zr

Element Number	Element Symbol	Element Name	Weight Concentration	Error
20	Ca	Calcium	30.9	0.2
16	S	Sulfur	16.8	0.3
26	Fe	Iron	20.1	0.3

13	Al	Aluminium	6.1	0.1
8	O	Oxygen	26.1	0.5

Image 4

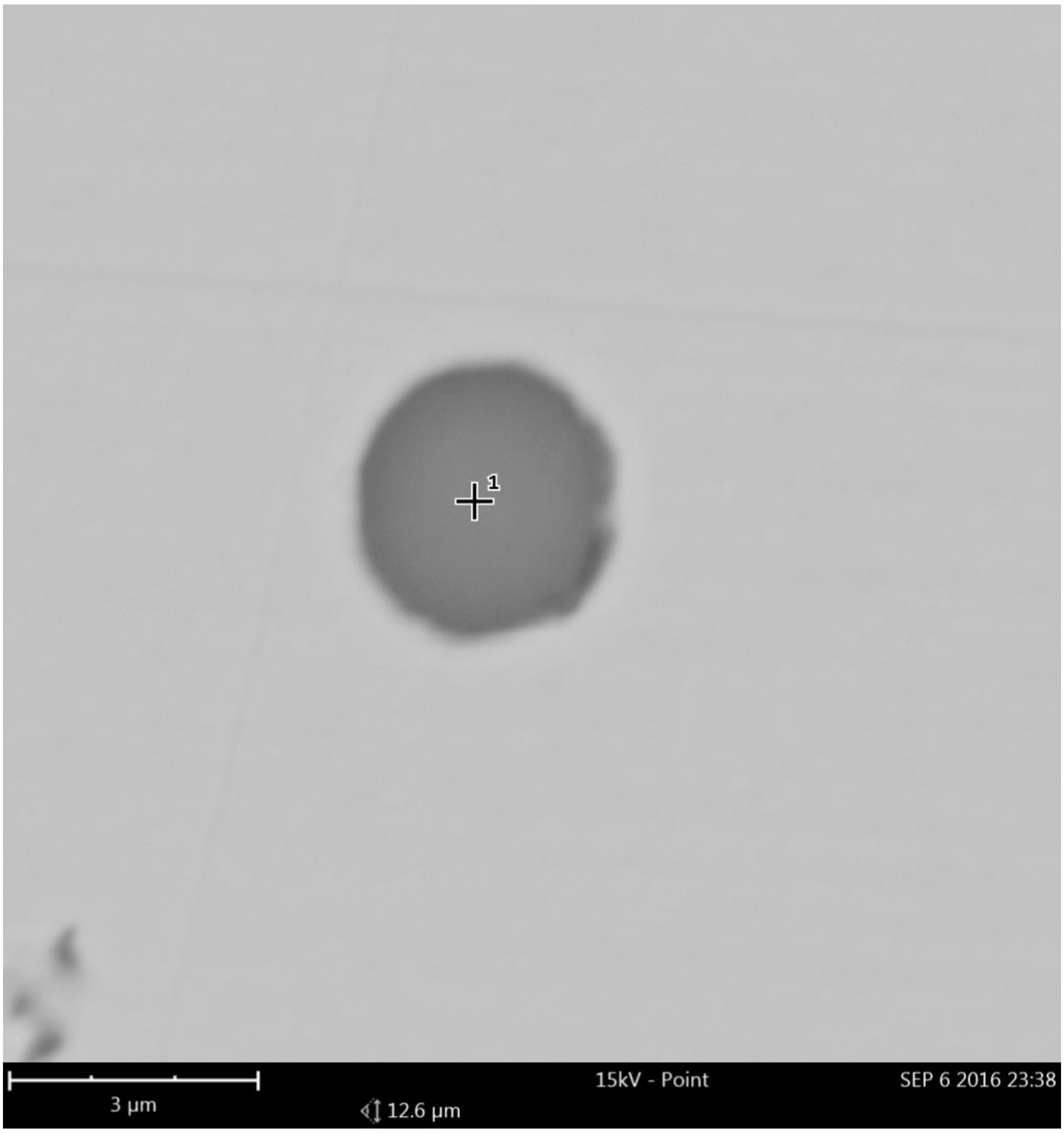

1. spot

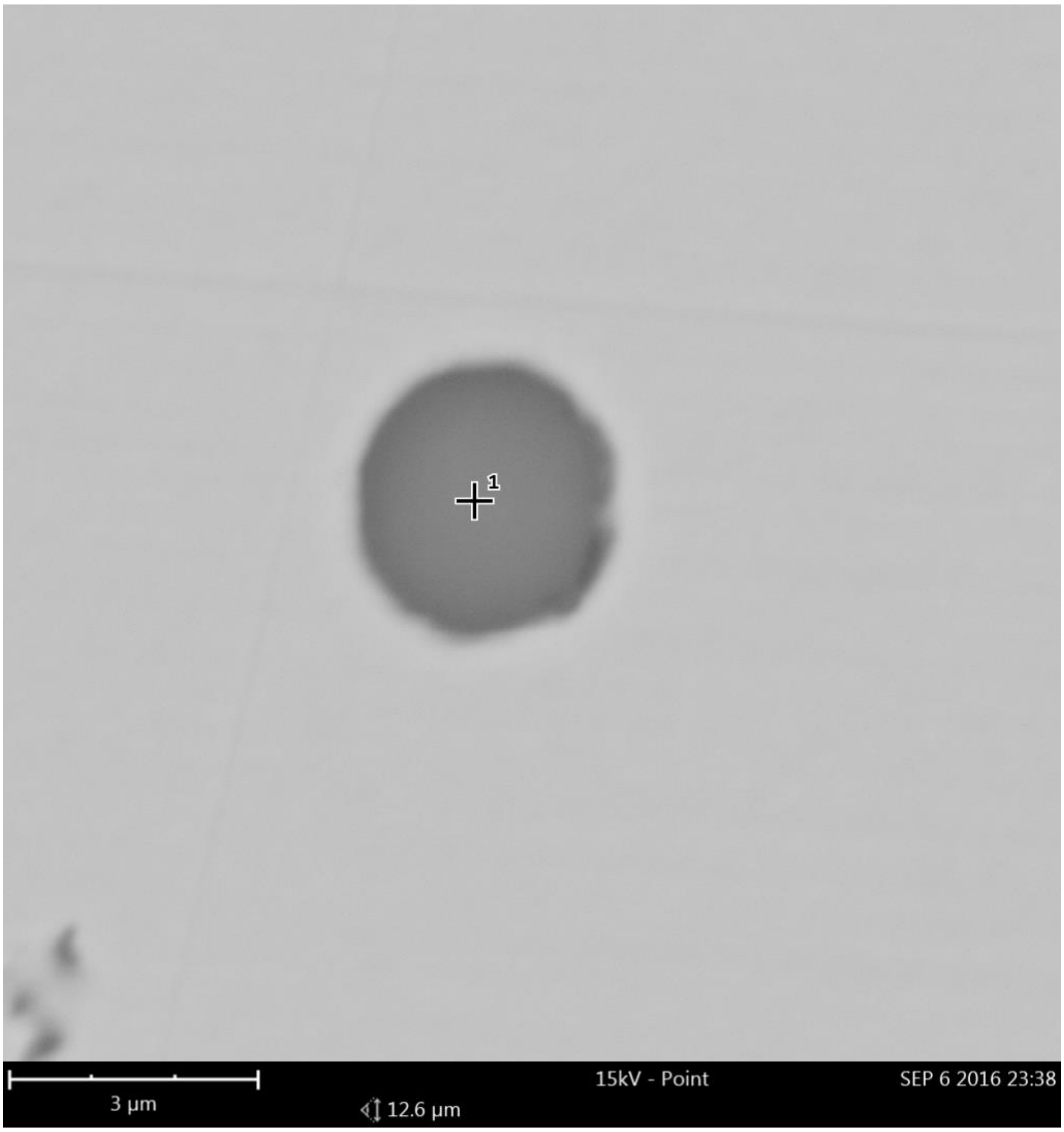

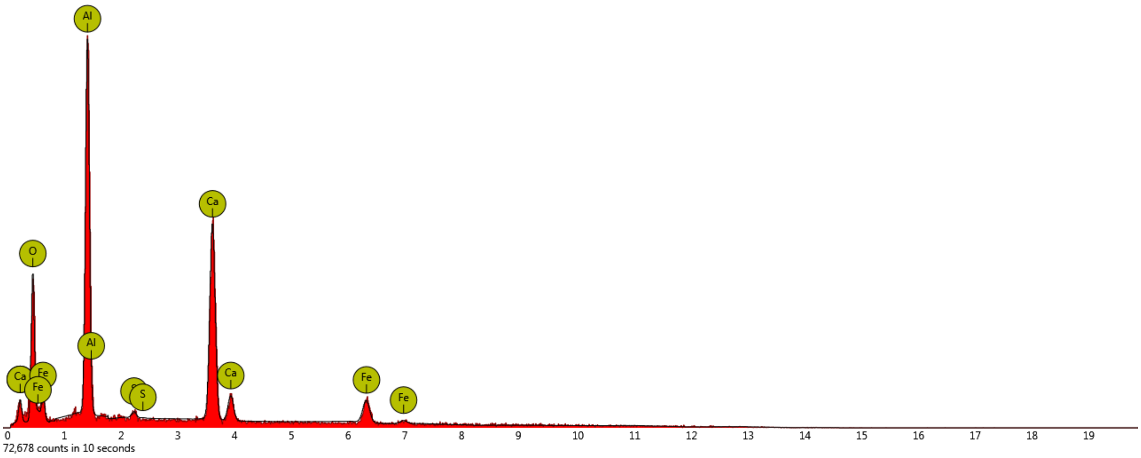

Disabled elements: As, B, Ba, Br, C, Cs, Dy, Er, F, Ga, Hg, La, Lu, Pb, Pm, Pt, Rb, Sb, Si, Sr, Te, Tm, Yb, Zr

Element Number	Element Symbol	Element Name	Weight Concentration	Error
13	Al	Aluminium	26.8	0.1
20	Ca	Calcium	22.9	0.2
8	O	Oxygen	40.2	0.6

26	Fe	Iron	9.5	0.3
16	S	Sulfur	0.5	1.9

Image 5

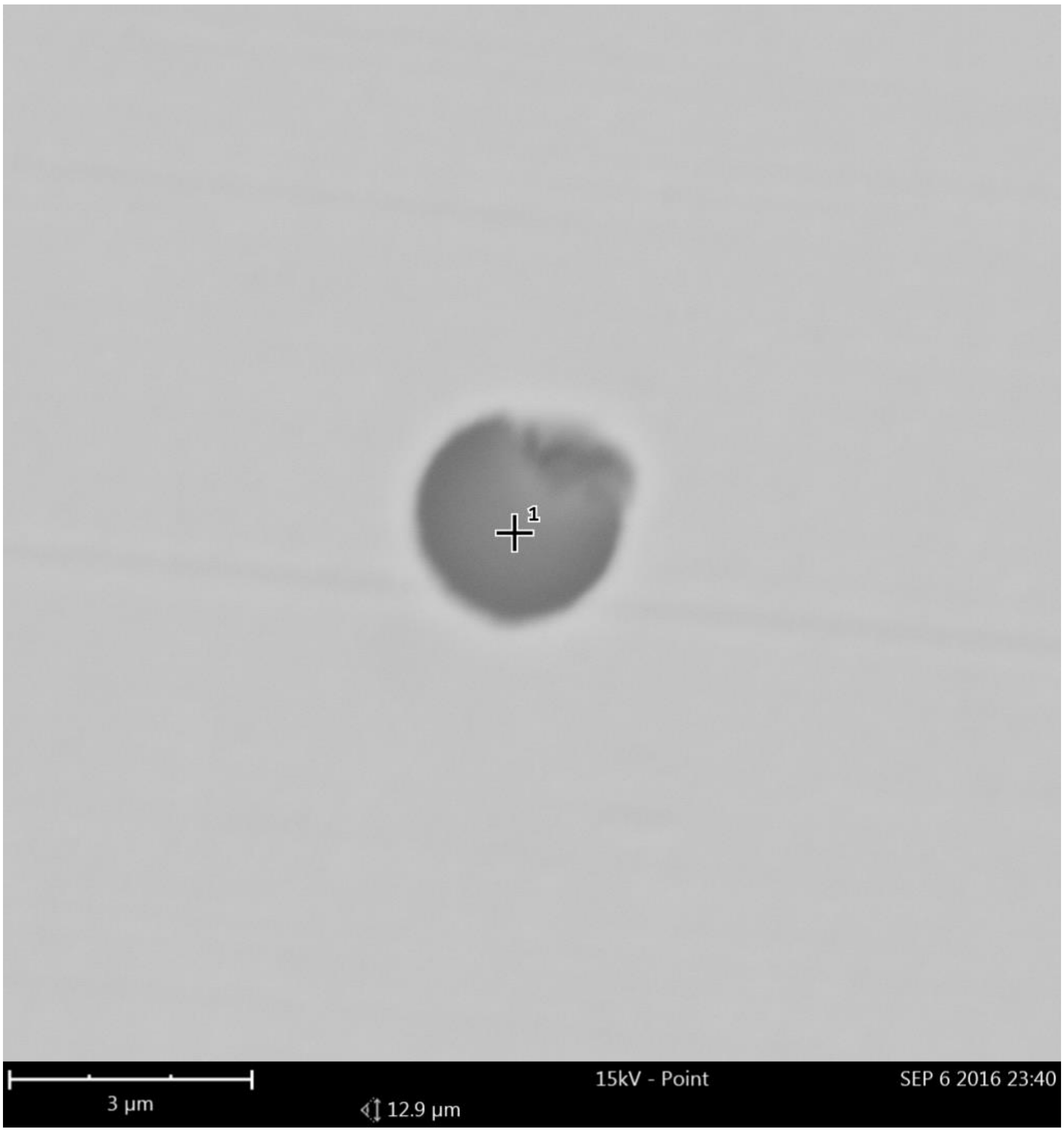

1. spot

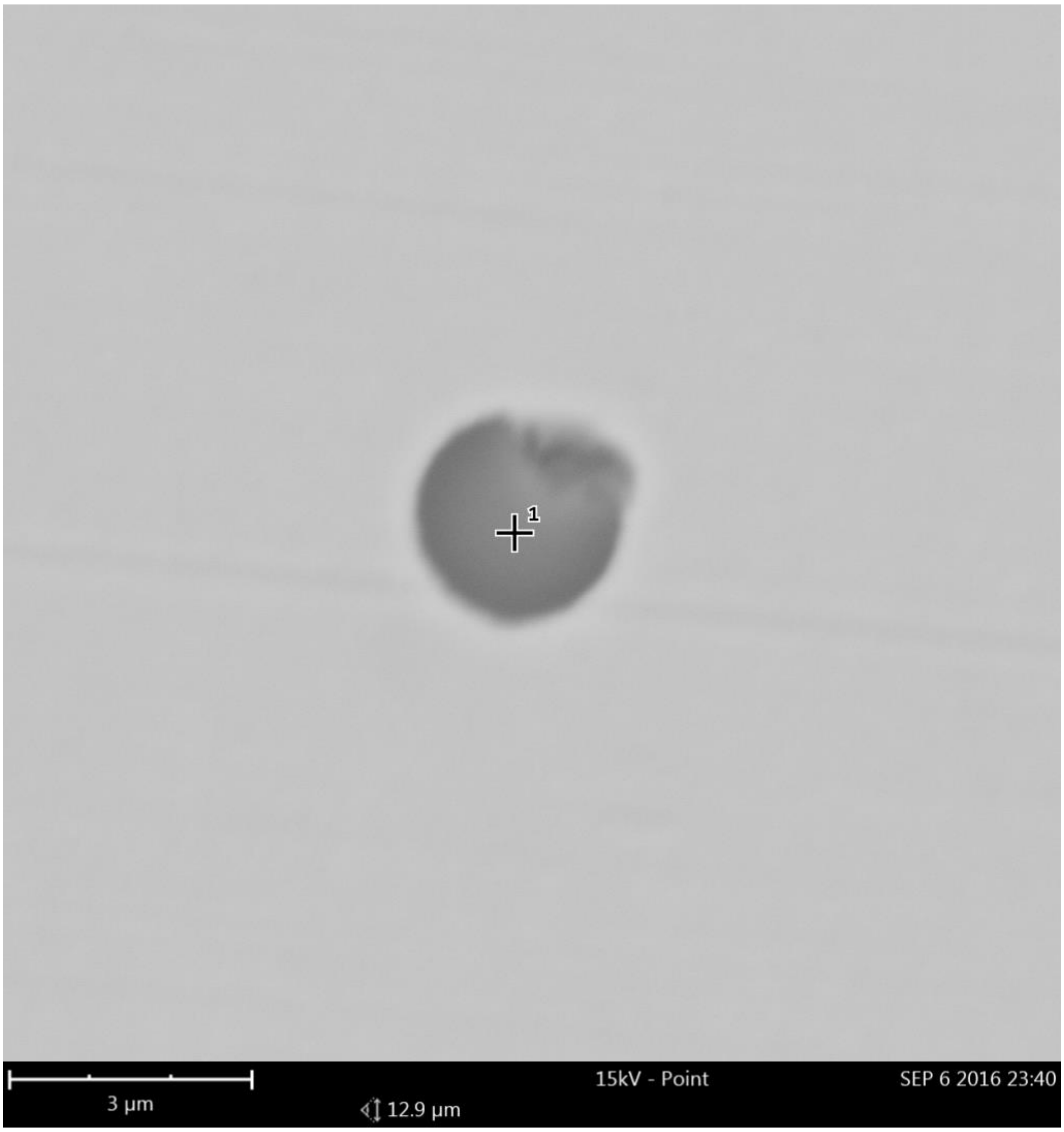

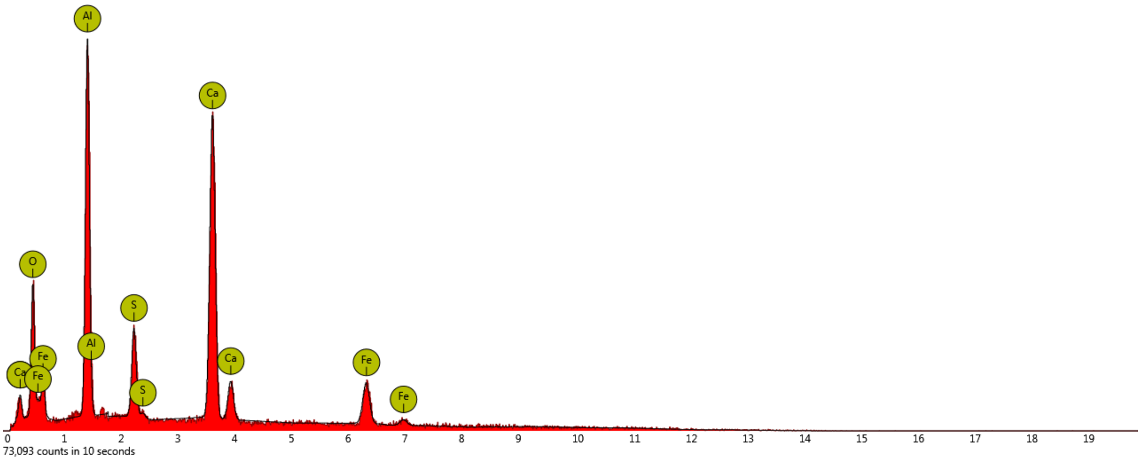

Disabled elements: As, B, Ba, Br, C, Cs, Dy, Er, F, Ga, Hg, La, Lu, Pb, Pm, Pt, Rb, Sb, Si, Sr, Te, Tm, Yb, Zr

Element Number	Element Symbol	Element Name	Weight Concentration	Error
20	Ca	Calcium	27.4	0.2
13	Al	Aluminium	21.4	0.1
8	O	Oxygen	32.1	0.5

16	S	Sulfur	5.6	0.3
26	Fe	Iron	13.6	0.4

SEM-EDS results of sample A2C3-3900s

Image 1

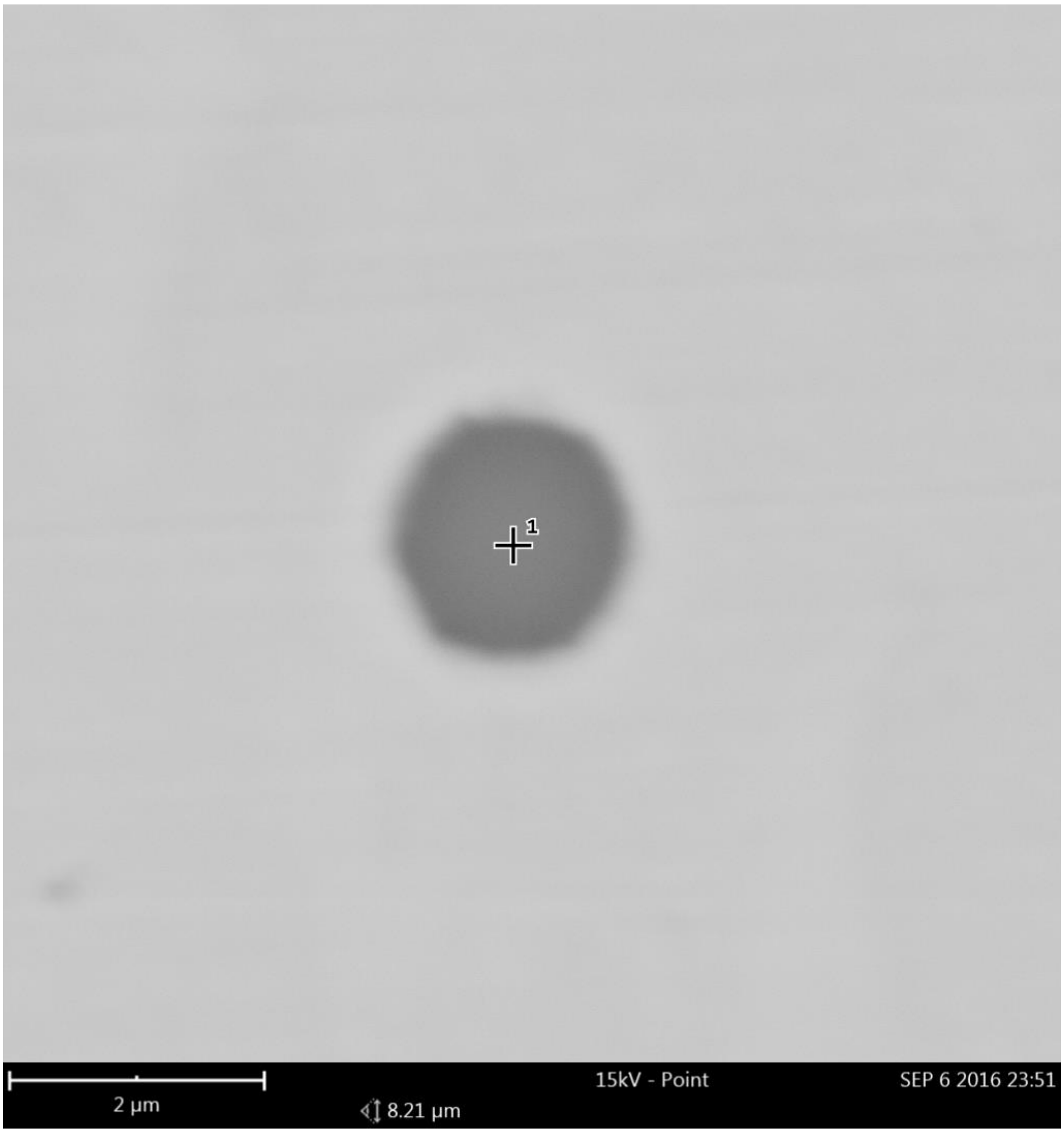

1. spot

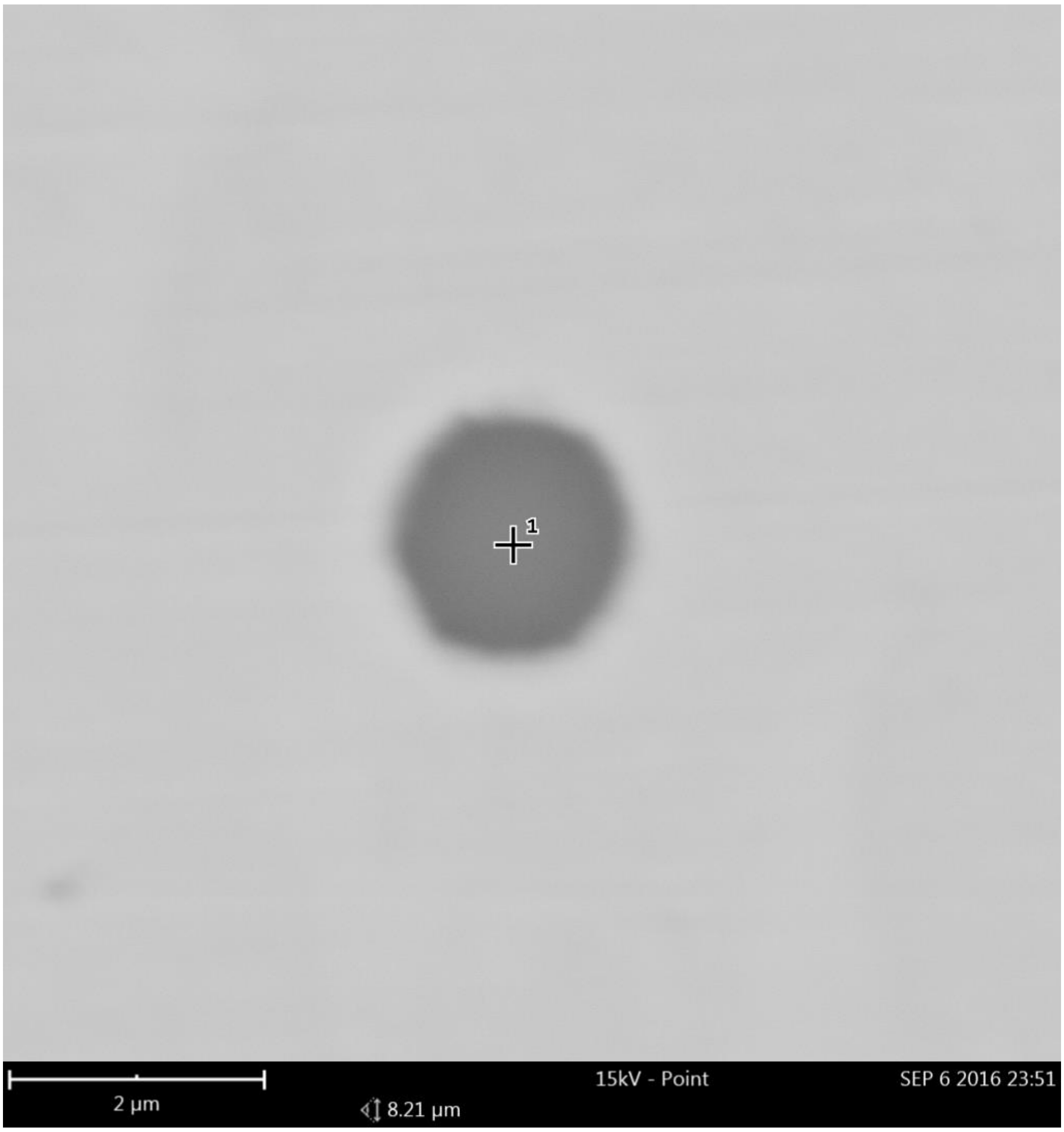

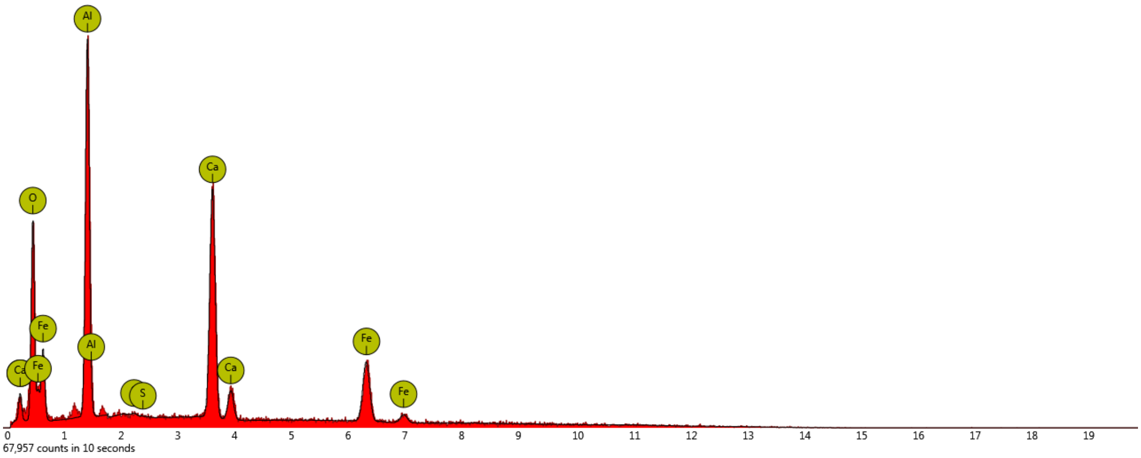

Disabled elements: As, B, Ba, Br, C, Cs, Dy, Er, F, Ga, Hg, La, Lu, Pb, Pm, Pt, Rb, Sb, Si, Sr, Te, Tm, Yb, Zr

Element Number	Element Symbol	Element Name	Weight Concentration	Error
13	Al	Aluminium	22.6	0.1
20	Ca	Calcium	20.5	0.2
8	O	Oxygen	37.3	0.5

26	Fe	Iron	19.6	0.3
16	S	Sulfur	0.1	1.0

Image 2

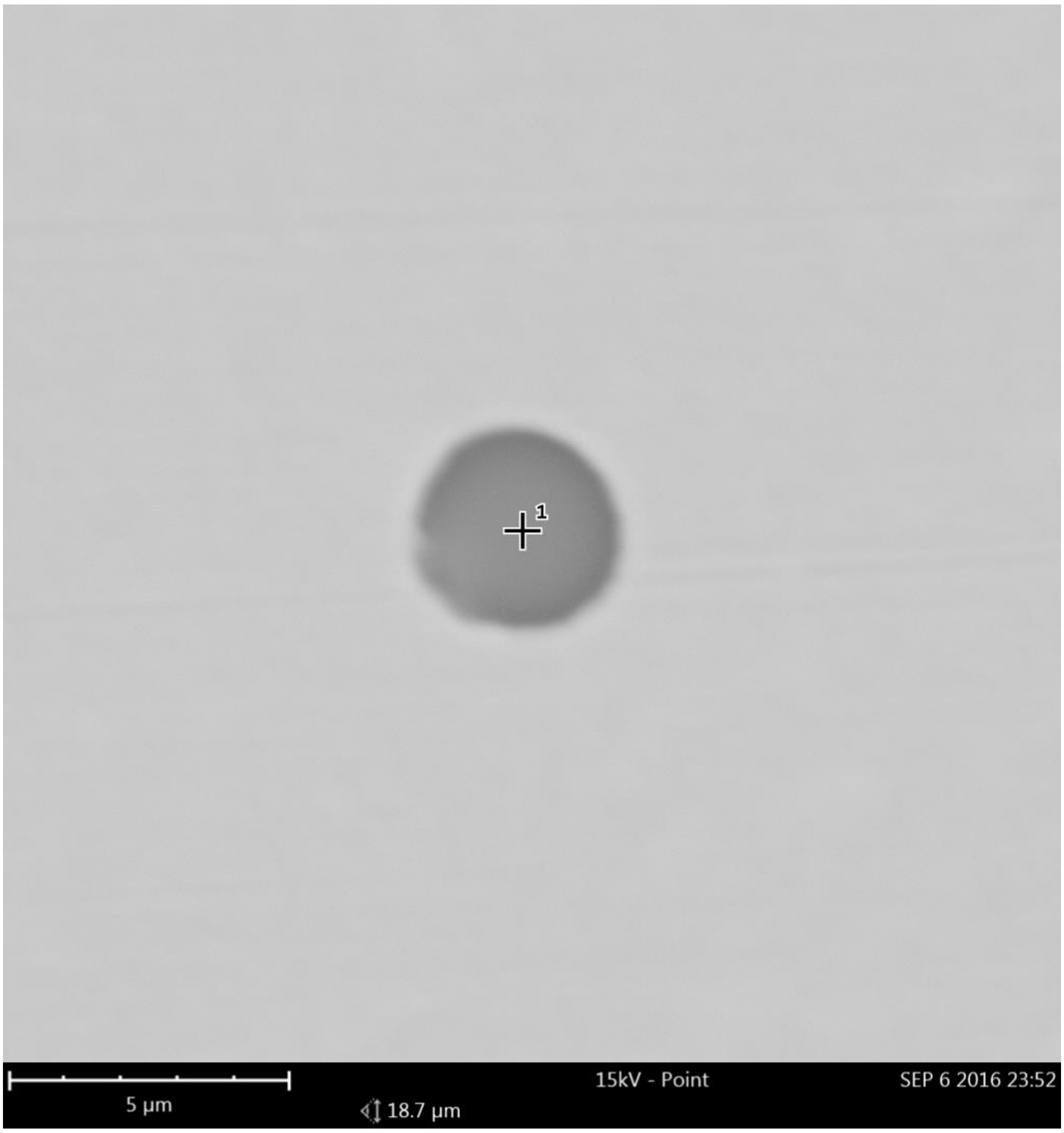

1. spot

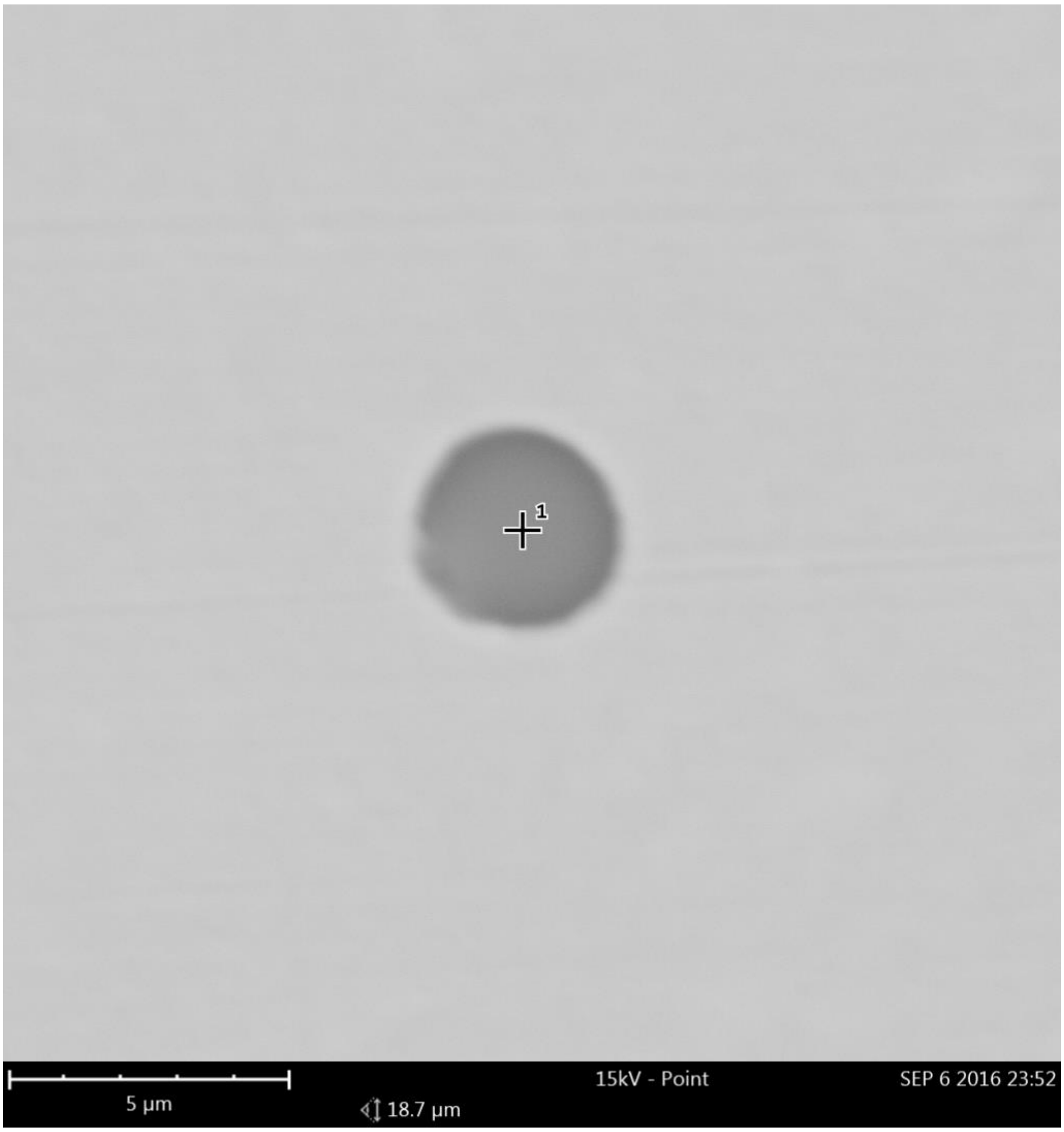

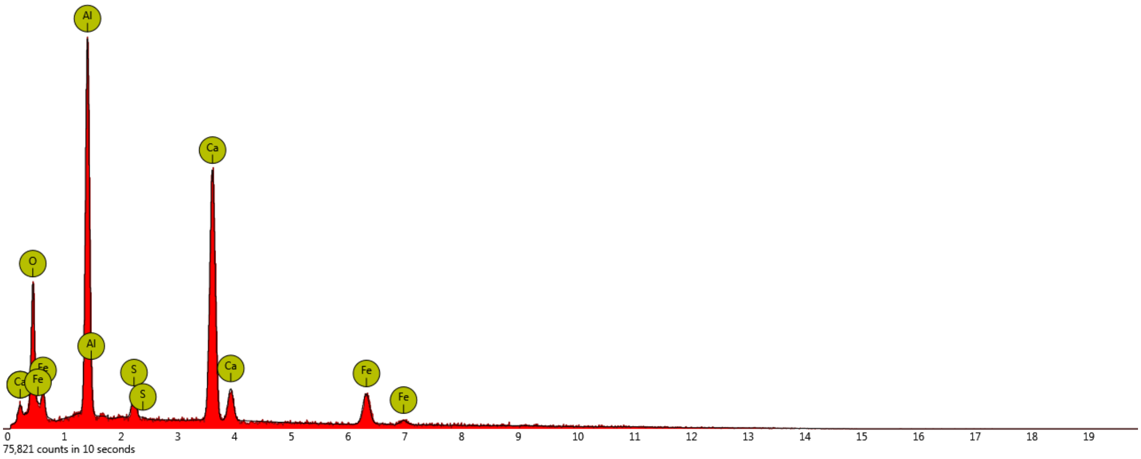

Disabled elements: As, B, Ba, Br, C, Cs, Dy, Er, F, Ga, Hg, La, Lu, Pb, Pm, Pt, Rb, Sb, Si, Sr, Te, Tm, Yb, Zr

Element Number	Element Symbol	Element Name	Weight Concentration	Error
13	Al	Aluminium	24.7	0.1
20	Ca	Calcium	26.5	0.2
8	O	Oxygen	35.1	0.5

26	Fe	Iron	11.8	0.3
16	S	Sulfur	1.9	0.3

Image 3

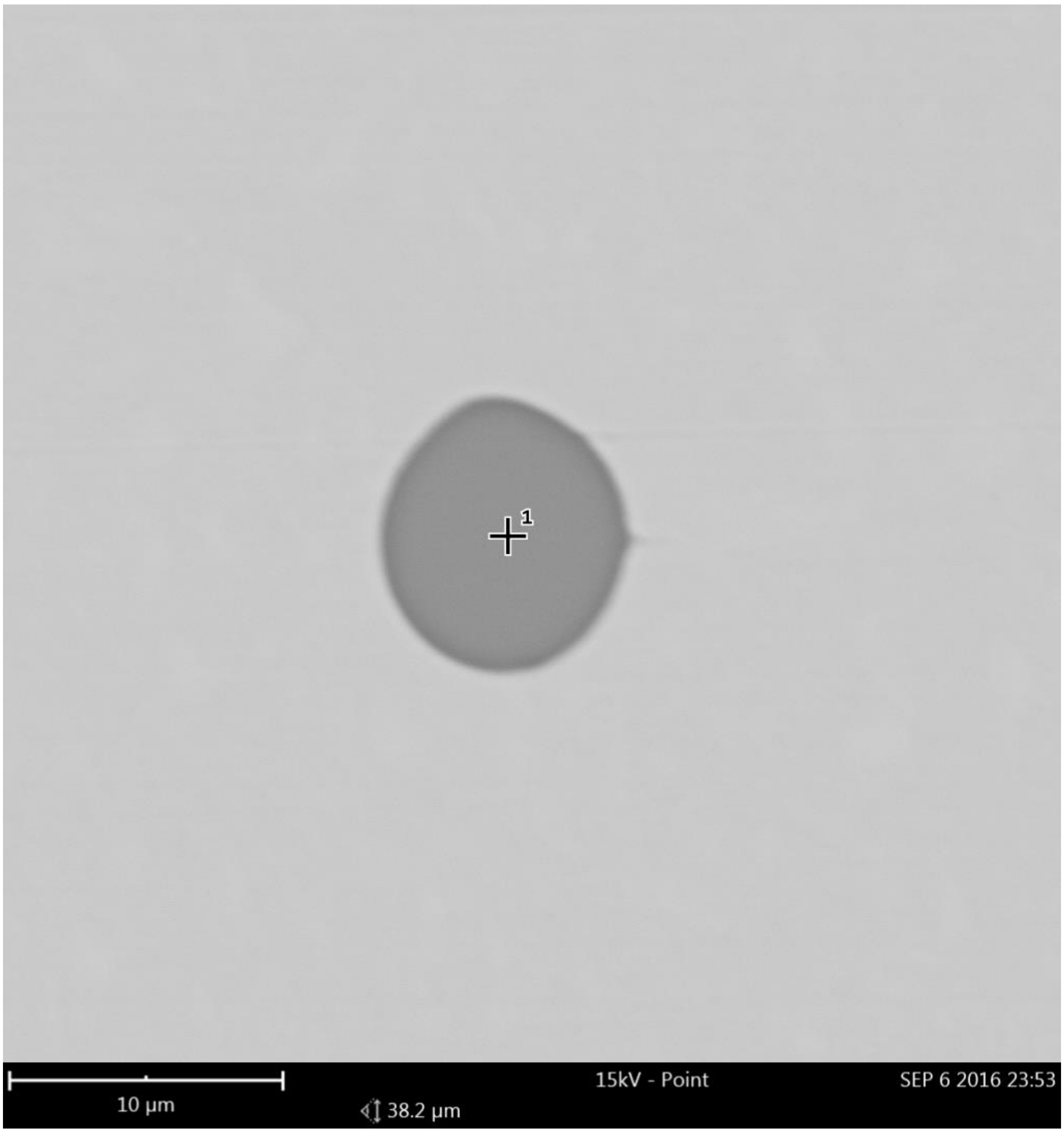

1. spot

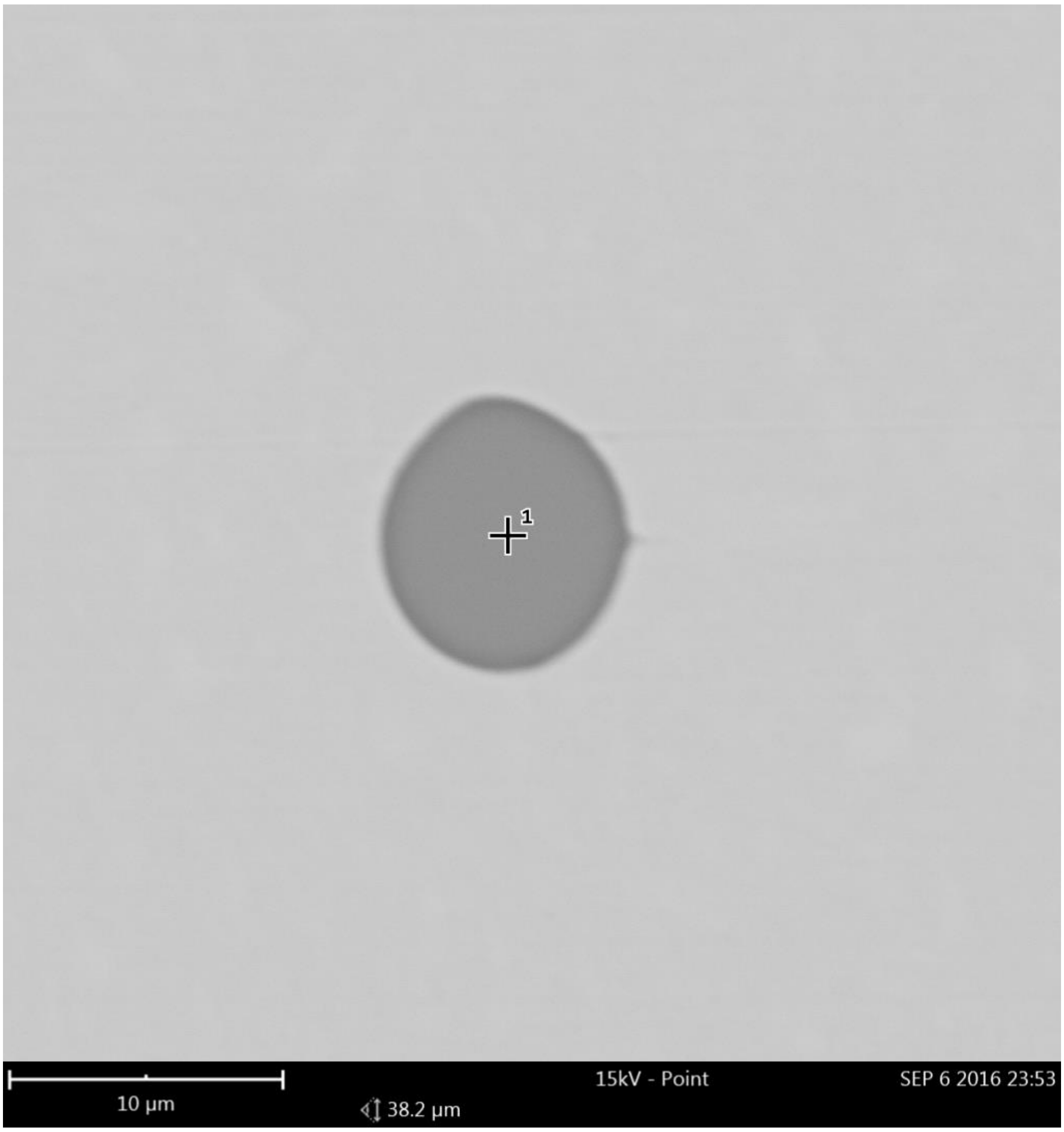

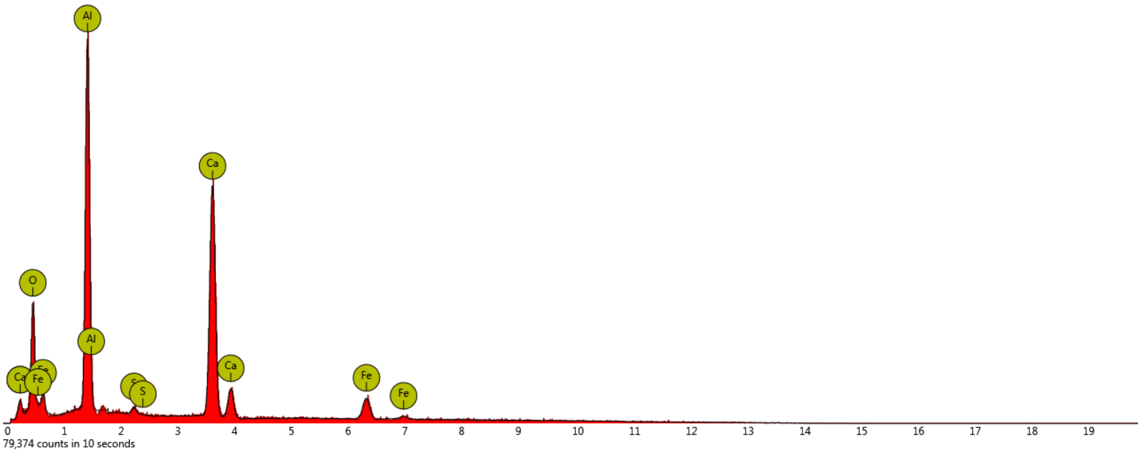

Disabled elements: As, B, Ba, Br, C, Cs, Dy, Er, F, Ga, Hg, La, Lu, Pb, Pm, Pt, Rb, Sb, Si, Sr, Te, Tm, Yb, Zr

Element Number	Element Symbol	Element Name	Weight Concentration	Error
13	Al	Aluminium	27.3	0.1
20	Ca	Calcium	28.0	0.2
8	O	Oxygen	34.8	0.5

26	Fe	Iron	9.3	0.4
16	S	Sulfur	0.6	0.8

Image 4

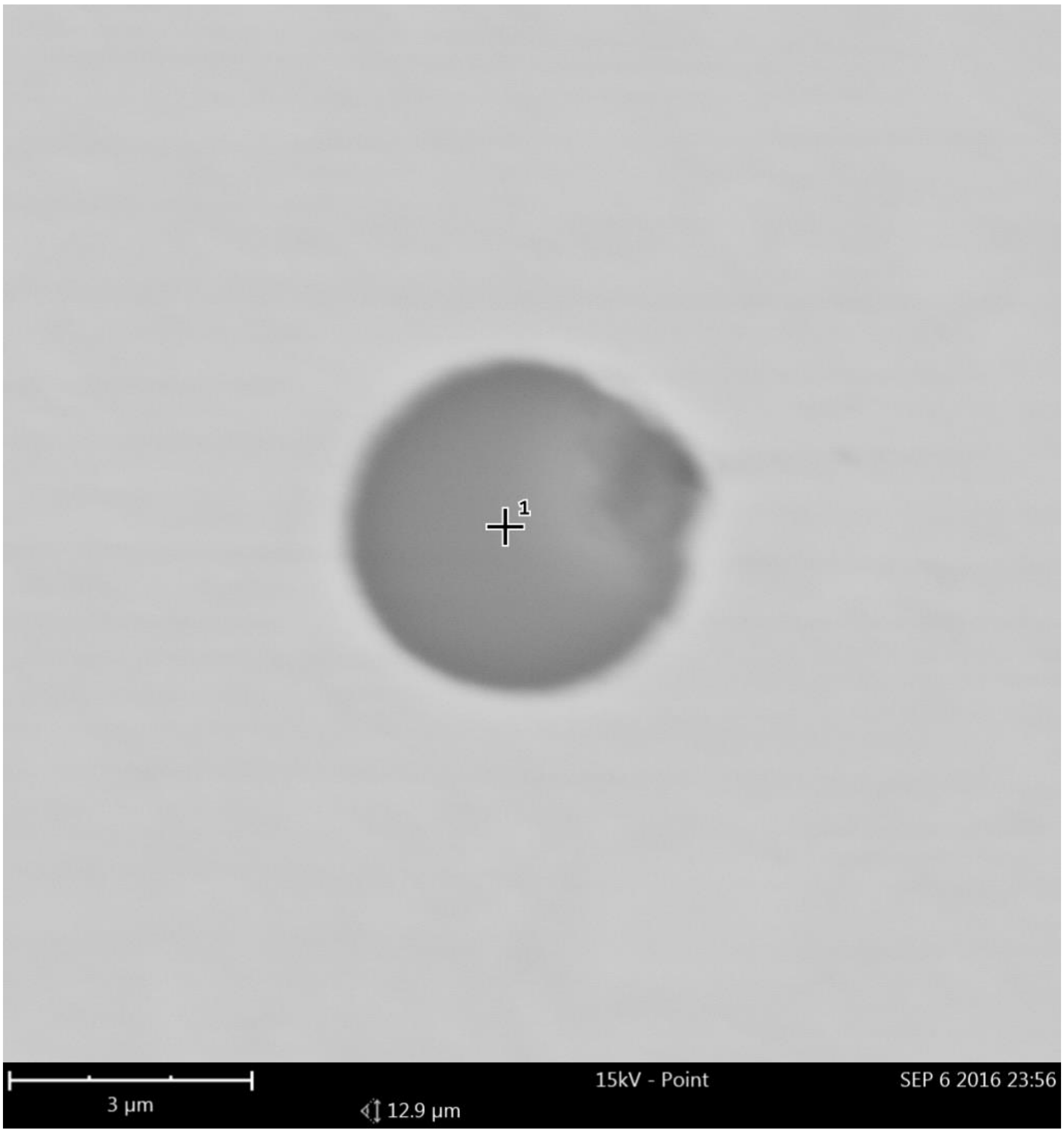

1. spot

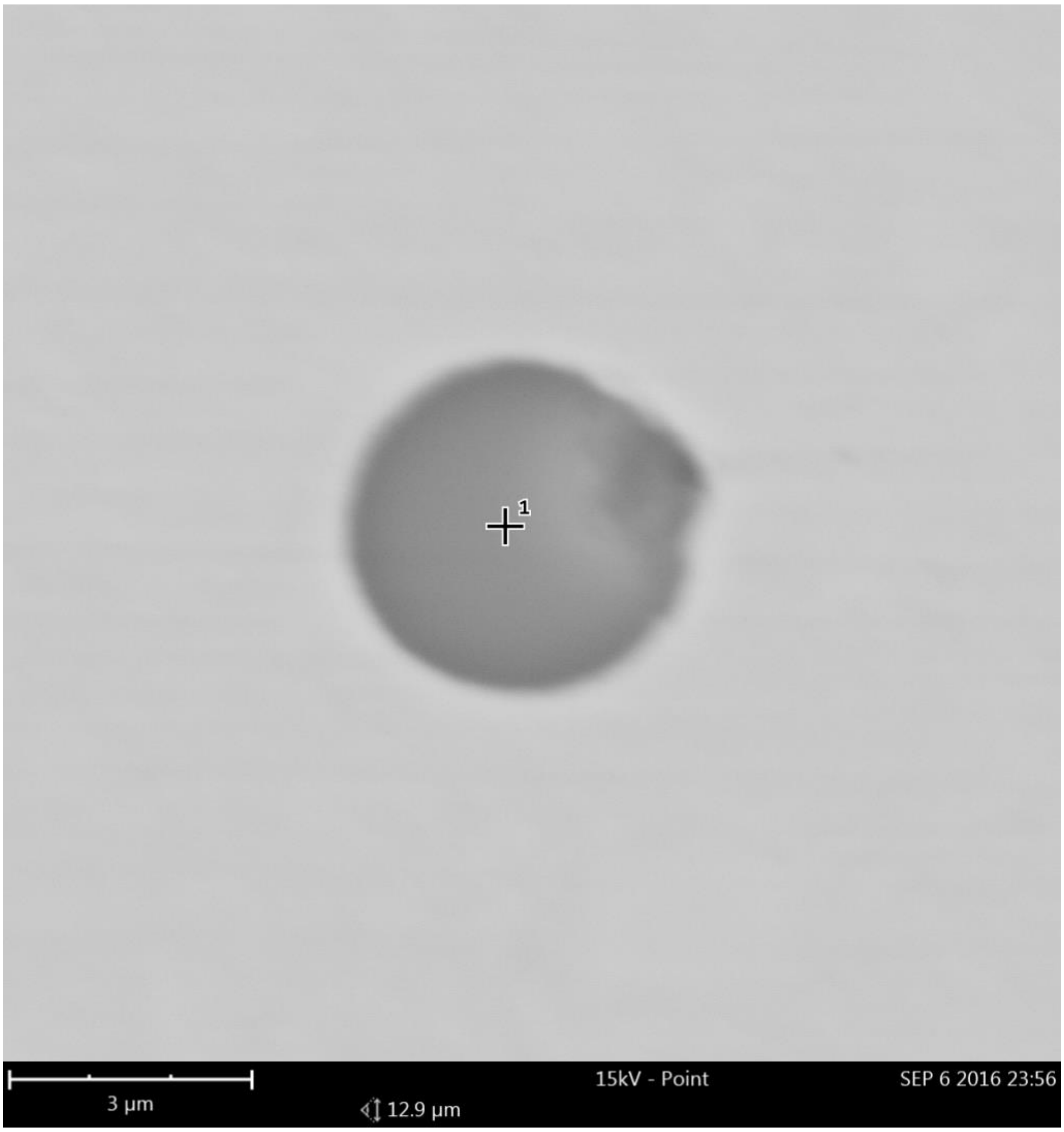

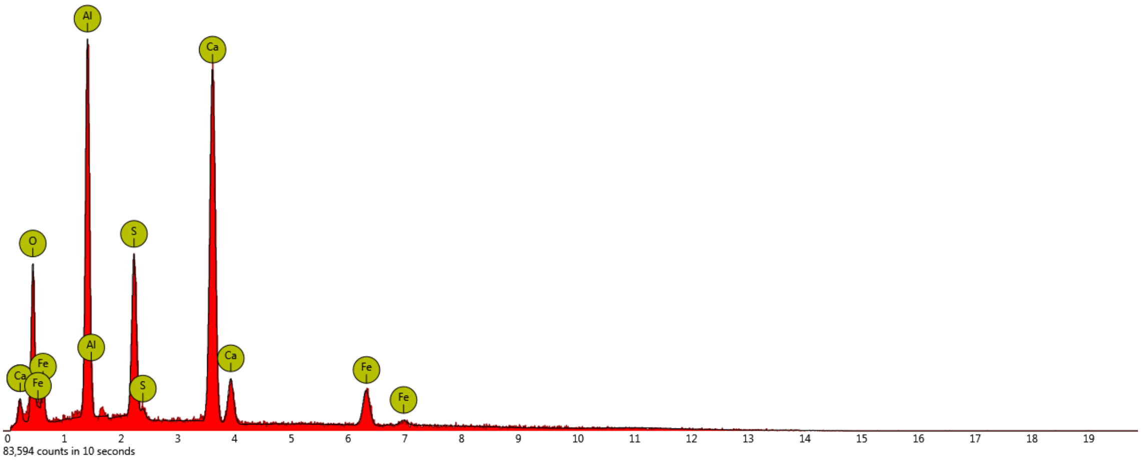

Disabled elements: As, B, Ba, Br, C, Cs, Dy, Er, F, Ga, Hg, La, Lu, Pb, Pm, Pt, Rb, Sb, Si, Sr, Te, Tm, Yb, Zr

Element Number	Element Symbol	Element Name	Weight Concentration	Error
20	Ca	Calcium	27.9	0.2
13	Al	Aluminium	18.3	0.1
16	S	Sulfur	8.6	0.3

8	O	Oxygen	35.0	0.5
26	Fe	Iron	10.2	0.3

Image 5

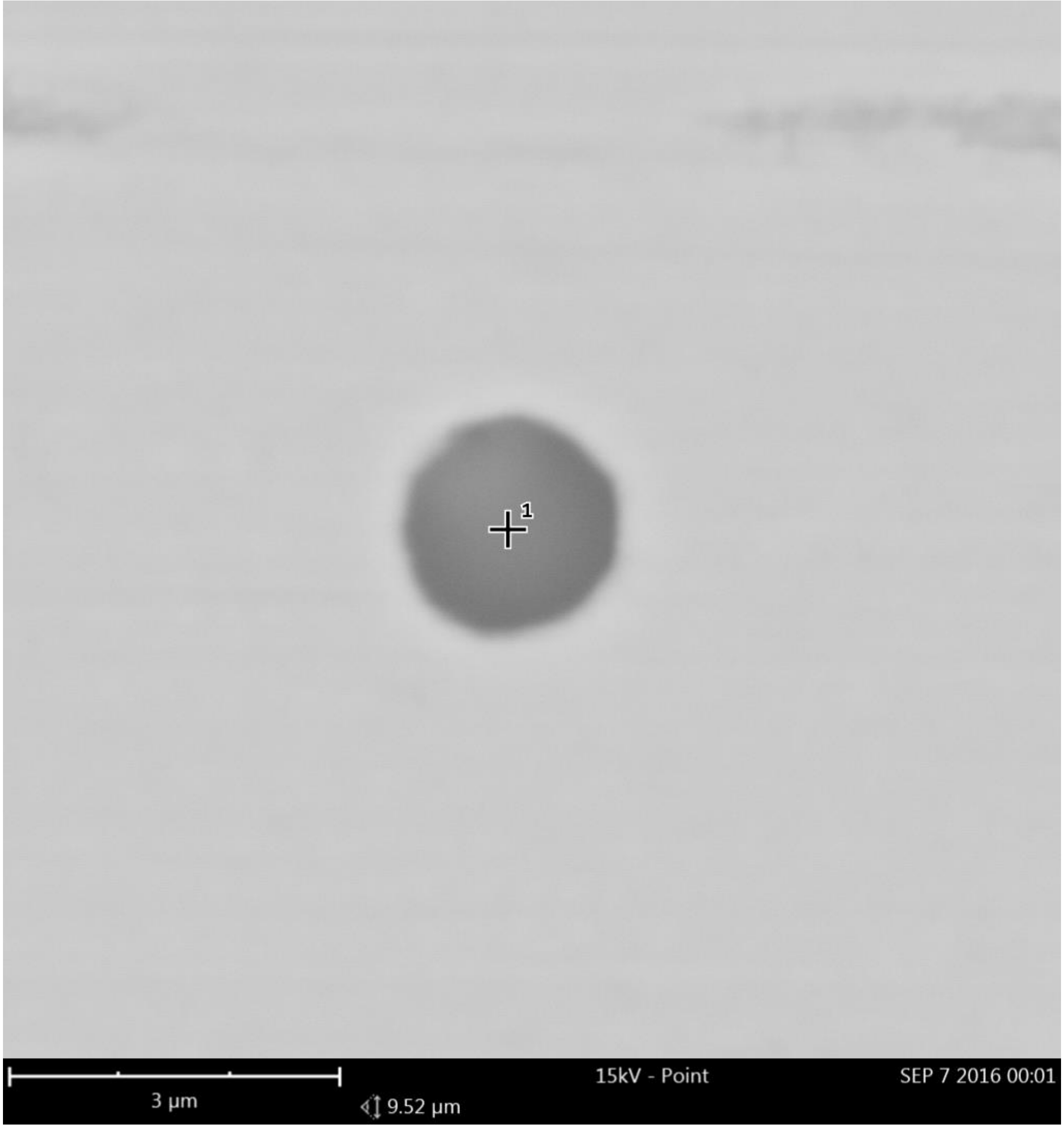

1. spot

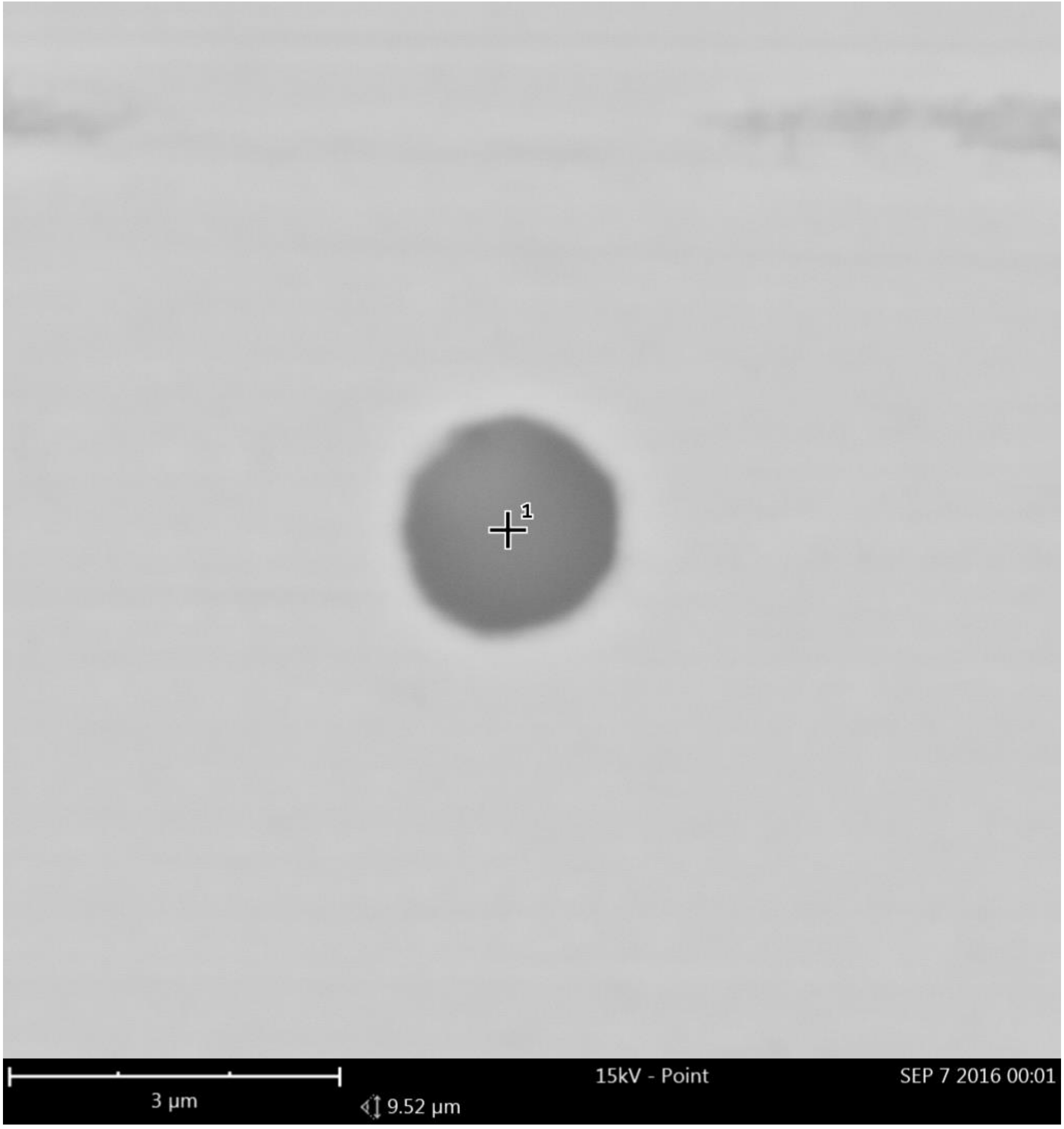

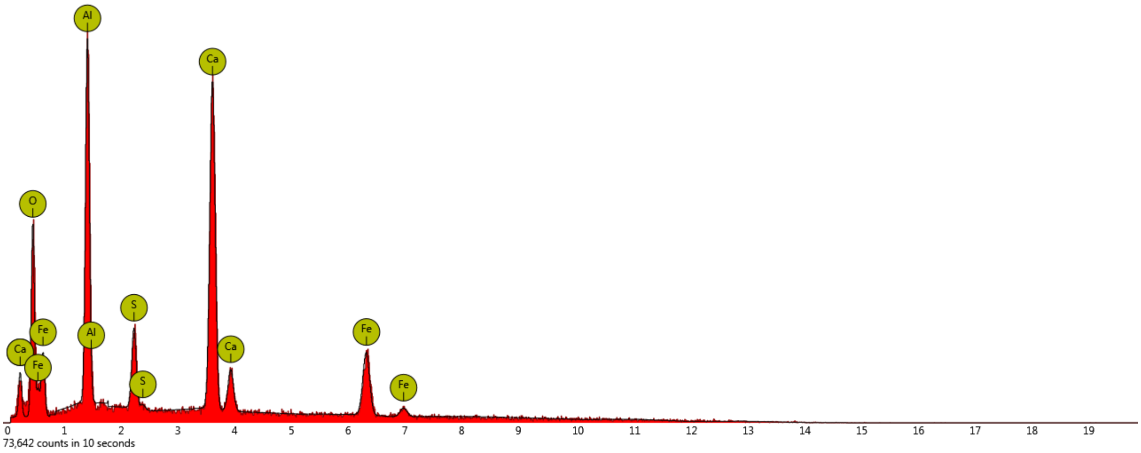

Disabled elements: As, B, Ba, Br, C, Cs, Dy, Er, F, Ga, Hg, La, Lu, Pb, Pm, Pt, Rb, Sb, Si, Sr, Te, Ti, Tm, Yb, Zr

**Element Number**	**Element Symbol**	**Element Name**	**Weight Concentration**	**Error**
20	Ca	Calcium	24.8	0.2
13	Al	Aluminium	18.2	0.1
8	O	Oxygen	35.4	0.6

26	Fe	Iron	17.4	0.3
16	S	Sulfur	4.2	0.4
